# Supplementary material for: Effect of weekend admission on process of care and clinical outcomes for the management of acute coronary syndromes: a retrospective analysis of three UK centres
Source: BMJ Open. 2017 Sep 29;7(9):e016866. doi: 10.1136/bmjopen-2017-016866 (PMC5640127; doi:10.1136/bmjopen-2017-016866)
Supplement: Supplementary file 1 [file bmjopen-2017-016866supp001.pdf]

Supplementary Material

Effect of Weekend Admission on Process of Care and Clinical Outcomes for the Management of Acute Coronary Syndromes

Glen P. Martin*; Tim Kinnaird*; Matthew Sperrin; Richard Anderson; Amr Gamal; Avais Jabbar; Chun Shing Kwok; Diane Barker; Grant Heatlie; Azfar G. Zaman; Mamas A. Mamas

* Joint first authors: these authors contributed equally to manuscript

Supplementary Figures

Supplementary Figure 1: Cumulative event rate for length of stay following weekend versus weekday admission across STEMI and NSTEMI/ UA subgroups.

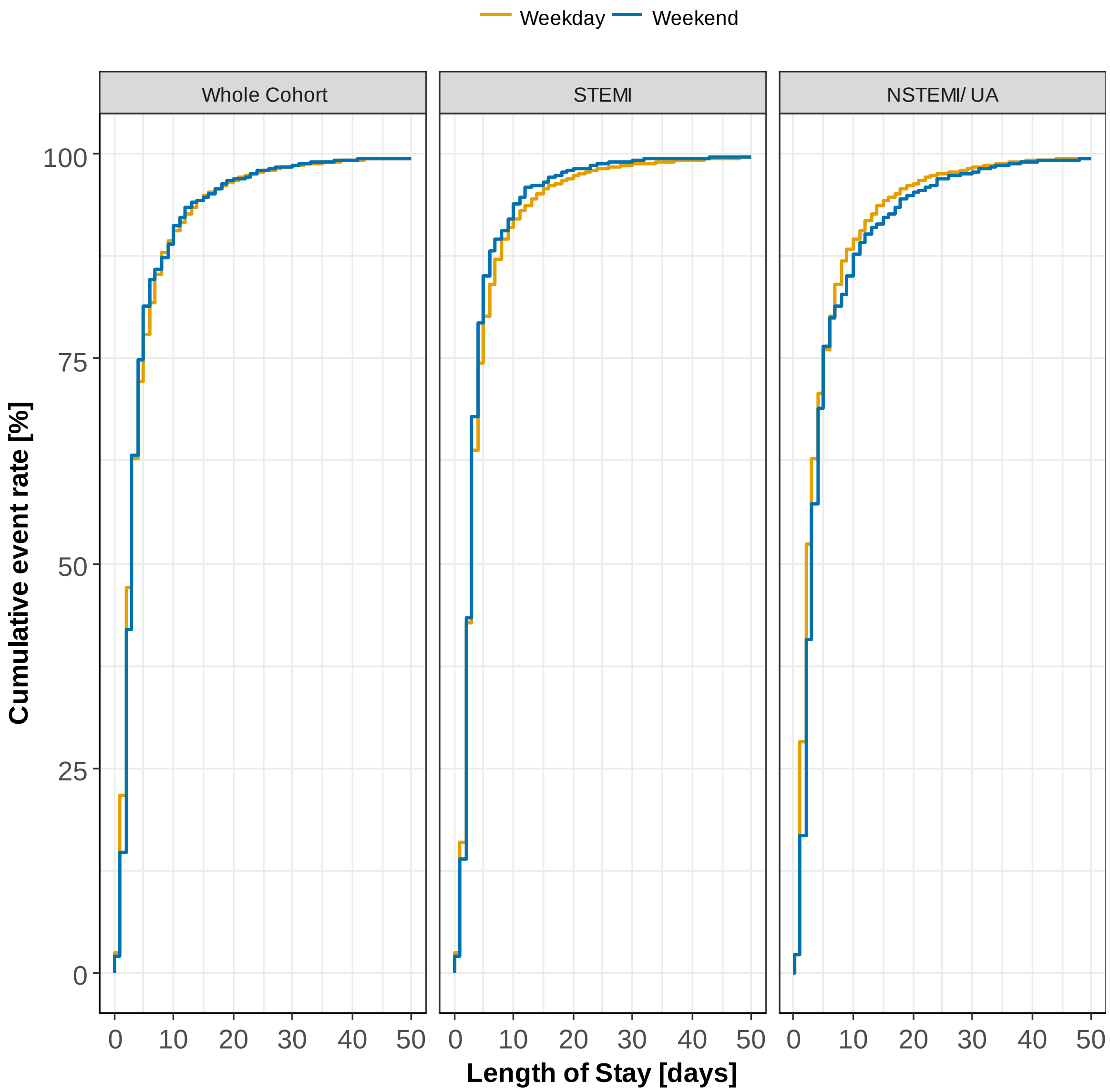

Supplementary Tables

Supplementary Table 1: Number of patients discharged within each time-window and PS adjusted hazard ratios (HR) from the time-dependent Cox proportional hazards models for length of stay, across STEMI and NSTEMI/ UA subgroups. Bold items indicate significant effects at the 5% level.

	**STEMI**		
**Length of stay**	**Discharged, n (%) Weekday Cohort**	**Discharged, n (%) Weekend Cohort**	**HR (95% CI)**
0 – 1 day	1074 (15.9%)	352 (13.9%)	**0.85 (0.75, 0.96)**
1 - 4 days	3953 (69.5%)	1659 (76.0%)	**1.13 (1.06, 1.19)**
4 - 50 days	1702 (98.3%)	513 (98.1%)	1.04 (0.94, 1.15)
	**NSTEMI/ UA**		
0 – 1 day	1733 (28.1%)	283 (16.7%)	**0.65 (0.57, 0.74)**
1 - 4 days	2628 (59.3%)	884 (62.7%)	**1.12 (1.04, 1.21)**
4 - 50 days	1770 (98.1%)	515 (97.9%)	0.93 (0.84, 1.03)

Supplementary Table 2: Proportions of NSTEMI/ UA patients receiving angiography within 24 and 72 hours across weekend admission groups.

**Time-to-angiography**	**Weekday NSTEMI/ UA**	**Weekend NSTEMI/ UA**	**p-value**
Within 24 hours	3242/ 4529 (71.6%)	662/ 1176 (56.3%)	<0.001
Within 72 hours	4125/ 4529 (91.1%)	1046/ 1176 (88.9%)	0.028

Supplementary Table 3: NSTEMI/UA patient characteristics across those that underwent coronary angiography within 24 hours compared with those after 24 hours.

**Variable**	**Angiography≤24 hours [n=3902]**	**Angiography>24 hours [n=1802]**	**p-value**
Age at Admission, mean (SD)	66.2 (12.4)	67.2 (12.0)	0.004
Male, n (%)	2807 (71.9)	1258 (69.8)	0.109
Caucasian, n (%)	3286 (84.2)	1101 (61.1)	<0.001
Admission Diagnosis			
Acute coronary syndrome, n (%)	3627 (93.0)	1459 (81.0)	<0.001
Chest pain unknown cause, n (%)	253 (6.48)	271 (15.0)	<0.001
Other, n (%)	23 (0.59)	72 (4.00)	<0.001
Previous AMI, n (%)	1169 (30.0)	551 (30.6)	0.659
Previous Angina, n (%)	1555 (39.9)	661 (36.7)	0.024
Hypertension, n (%)	2505 (64.2)	1142 (63.4)	0.567
Hypercholesterolemia, n (%)	2167 (55.5)	877 (48.7)	<0.001
PVD, n (%)	297 (7.61)	164 (9.10)	0.062
Cerebrovascular Disease, n (%)	339 (8.69)	161 (8.93)	0.798
Asthma or COPD, n (%)	647 (16.6)	311 (17.3)	0.550
Chronic Renal Failure, n (%)	126 (3.23)	129 (7.16)	<0.001
Heart Failure, n (%)	147 (3.77)	111 (6.16)	<0.001
Enzymes Elevated, n (%)	3382 (86.7)	1611 (89.4)	0.004
Previous/ Current Smoker, n (%)	2600 (66.6)	1210 (67.1)	0.724
Cholesterol, mean (SD)	4.53 (1.38)	4.48 (1.33)	0.379
Diabetes			
Dietary control, n (%)	157 (4.02)	81 (4.50)	0.449
Oral medicine, n (%)	473 (12.1)	248 (13.8)	0.091
Insulin, n (%)	223 (5.72)	123 (6.83)	0.116
Insulin and medication, n (%)	59 (1.51)	62 (3.44)	<0.001
Previous PCI, n (%)	783 (20.1)	343 (19.0)	0.382
Previous CABG, n (%)	274 (7.02)	192 (10.7)	<0.001
Systolic BP, mean (SD)	136.6 (24.6)	140.3 (25.6)	<0.001
Heart Rate, mean (SD)	71.4 (15.4)	75.8 (18.0)	<0.001
Admitting Consultant			
Cardiologist, n (%)	3893 (99.8)	1708 (94.8)	<0.001
Other general physician, n (%)	4 (0.10)	65 (3.61)	<0.001
Other, n (%)	3 (0.08)	21 (1.17)	<0.001
Beta Blocker, n (%)	1458 (37.4)	713 (39.6)	0.118
Statin, n (%)	2034 (52.1)	988 (54.8)	0.061

Glucose, mean (SD)	7.45 (3.48)	8.16 (4.13)	<0.001
Height, mean (SD)	169.7 (9.90)	169.3 (10.1)	0.251
Weight, mean (SD)	82.7 (17.7)	82.6 (18.5)	0.890
Family History of CHD, n (%)	1942 (49.8)	774 (43.0)	<0.001
Creatinine, mean (SD)	97.6 (54.9)	103.4 (80.4)	0.006
Haemoglobin, mean (SD)	137.4 (18.0)	135.7 (19.6)	0.002
Killip class			
No evidence of Heart Failure, n (%)	3083 (79.0)	1108 (61.5)	<0.001
Basal Crepitations, n (%)	101 (2.59)	172 (9.54)	<0.001
Pulmonary Oedema, n (%)	139 (3.56)	105 (5.83)	<0.001
Cardiogenic Shock, n (%)	14 (0.36)	7 (0.39)	0.999
Cardiac Arrest, n (%)	100 (2.56)	46 (2.55)	0.999
Thienopyridene, n (%)	3384 (86.7)	1662 (92.2)	<0.001

AMI: Acute Myocardial Infarction; BP: Blood Pressure; CABG: Coronary Artery Bypass Graft; CHD: Coronary Heart Disease; COPD: Chronic Obstructive Pulmonary Disease; LVEF: Left Ventricular Ejection Fraction; MI: Myocardial Infarction; PCI: Percutaneous Coronary Intervention; PVD: Peripheral Vascular Disease
