# Supplementary material for: Effect of weekend admission on process of care and clinical outcomes for the management of acute coronary syndromes: a retrospective analysis of three UK centres
Source: BMJ Open. 2017 Sep 29;7(9):e016866. doi: 10.1136/bmjopen-2017-016866 (PMC5640127; doi:10.1136/bmjopen-2017-016866)
Supplement: Author's manuscript [file bmjopen-2017-016866.draft_revisions.pdf]

BMJ Open is committed to open peer review. As part of this commitment we make the peer review history of every article we publish publicly available.

When an article is published we post the peer reviewers' comments and the authors' responses online. We also post the versions of the paper that were used during peer review. These are the versions that the peer review comments apply to.

The versions of the paper that follow are the versions that were submitted during the peer review process. They are not the versions of record or the final published versions. They should not be cited or distributed as the published version of this manuscript.

BMJ Open is an open access journal and the full, final, typeset and author-corrected version of record of the manuscript is available on our site with no access controls, subscription charges or pay-per-view fees (http://bmjopen.bmj.com).

If you have any questions on BMJ Open's open peer review process please email editorial.bmjopen@bmj.com

BMJ Open

Effect of Weekend Admission on Process of Care and Clinical Outcomes for the Management of Acute Coronary Syndromes: a retrospective analysis of three UK centres

Journal:	*BMJ Open*
Manuscript ID	bmjopen-2017-016866
Article Type:	Research
Date Submitted by the Author:	15-Mar-2017
Complete List of Authors:	Martin, Glen; University of Manchester, Health eResearch Centre, Farr Institute<br>Kinnaird, Tim; Keele University, Keele Cardiovascular Research Group; University Hospital of Wales, Department of Cardiology<br>Sperrin, Matthew; University of Manchester, Health eResearch Centre, Farr Institute<br>Anderson, Richard; University Hospital of Wales, Department of Cardiology<br>Gamal, Amr; Newcastle University, Department of Cardiology<br>Jabbar, Avais; Newcastle University, Department of Cardiology<br>Kwok, Chun; Keele University, Keele Cardiovascular Research Group<br>Barker, Diane; Keele University, Keele Cardiovascular Research Group<br>Heatlie, Grant; Keele University, Keele Cardiovascular Research Group<br>Zaman, Azfar; Freeman Hospital, Department of Cardiology<br>Mamas, Mamas; University of Manchester, Health eResearch Centre, Farr Institute; Keele University, Keele Cardiovascular Research Group
**Primary Subject Heading**:	Health informatics
Secondary Subject Heading:	Cardiovascular medicine
Keywords:	Myocardial infarction < CARDIOLOGY, Coronary heart disease < CARDIOLOGY, Human resource management < HEALTH SERVICES ADMINISTRATION & MANAGEMENT

SCHOLARONE™
Manuscripts

Effect of Weekend Admission on Process of Care and Clinical Outcomes for the Management of Acute Coronary Syndromes: a retrospective analysis of three UK centres

Glen P. Martin, MSc *[1]; Tim Kinnaird, MBBCh, MD *[2, 3]; Matthew Sperrin, PhD [1]; Richard Anderson, BSc, MBBS, MD [3]; Amr Gamal MBBS [4]; Avais Jabbar, MBBS [4]; Chun Shing Kwok, MBBS, BSc, MSc [2]; Diane Barker, MB ChB, MD [2]; Grant Heatlie, BSc, PhD, MBBS [2]; Azfar G. Zaman, BSc, MBChB, MD [4]; Mamas A. Mamas, MA, DPhil, FRCP [1, 2]

* Joint first authors: these authors contributed equally to manuscript

1: Farr Institute, Faculty of Biology, Medicine and Health, University of Manchester, Manchester Academic Health Science Centre, Manchester, United Kingdom

2: Keele Cardiovascular Research Group, Institute of Applied Clinical Science, Keele University, Stoke-on-Trent, United Kingdom

3: Department of Cardiology, University Hospital of Wales, Cardiff, United Kingdom

4: Department of Cardiology, Freeman Hospital and Institute of Cellular Medicine, Newcastle University, Newcastle-upon-Tyne, United Kingdom

Manuscript Word Count: 3038

Running title: Effect of weekend admission on outcomes after ACS

Corresponding author:
Mamas A. Mamas

Professor of Cardiology / Honorary Consultant Cardiologist

Keele Cardiovascular Research Group, Guy Hilton Research Centre, Thornburrow Drive,

Keele University, Stoke-on-Trent, ST4 7QB, UK

Email: mamasmamas1@yahoo.co.uk

For peer review only

For peer review only - http://bmjopen.bmj.com/site/about/guidelines.xhtml

Abstract

Objective

The effect of weekend versus weekday admission following acute coronary syndrome (ACS) on process of care and mortality remains controversial. This study aimed to investigate the "weekend-effect" on outcomes using a multicentre dataset of ST-elevation myocardial infarction (STEMI) and non-ST-elevation myocardial infarction/ unstable angina (NSTEMI/ UA) patients.

Methods

The association between weekend admission and hospital mortality, long-term survival and several processes of care indicators was studied using data from three UK contributing centres to the Myocardial Ischaemia National Audit Project. Propensity score stratification was used to adjust for observed confounding. Logistic regression was used to model binary outcomes and time-to-event endpoints were modelled using Cox proportional hazards.

Results

Between January 2010 and March 2016, 17,705 admissions met the study inclusion criteria, 4,327 of which were at a weekend. After PS stratification adjustment, outcomes were similar between weekend and weekday admission across STEMI and NSTEMI/ UA patients. Weekend admissions were less likely to be discharged within one day (hazard ratio 0.72, 95% CI: 0.66, 0.78), but after 4 days the length of stay was similar (hazard ratio 0.97, 95% CI: 0.90, 1.04). Fewer NSTEMI/ UA patients received angiography between 0 and 24 hours at a weekend (hazard ratio 0.71, 95% CI: 0.65, 0.77). Weekend STEMI patients were less likely to undergo an angiogram within 1 hour, but there was no significant difference after this time point.

For peer review only - http://bmjopen.bmj.com/site/about/guidelines.xhtml

Conclusion

ACS patients had similar mortality and processes of care when admitted on a weekend compared with a weekday. There was evidence of a delay to angiography for NSTEMI/ UA patients admitted at the weekend.

Keywords

Myocardial infarction – Process of care – Acute Coronary Syndrome - Resource management – Weekend effect

For peer review only

Article Summary

Strengths and limitations of this study

- An analysis of a weekend effect after acute coronary syndromes in contemporary practice.
- This study analysed data derived from a robust national database (MINAP), of three large tertiary interventional centres.
- Included both ST elevation myocardial infarction cases and non-ST elevation myocardial infarction/ unstable angina cases.
- Any analysis of a weekend effect will be confounded by patient demographics, healthcare systems and the hospitals involved, thus limiting the generalisability of the results.
- Due to the observational nature of the study, unmeasured confounders potentially influence the conclusions.

For peer review only - http://bmjopen.bmj.com/site/about/guidelines.xhtml

Introduction

A timely intervention following presentation with an acute coronary syndrome (ACS) can mitigate loss of cardiac function and reduce morbidity and mortality [1,2]. Invasive procedures such as primary percutaneous coronary intervention (PCI) are central to the care of patients presenting with ST-elevation myocardial infarction (STEMI) [3] and guidelines also recommend an early invasive strategy for non-ST-elevation myocardial infarction (NSTEMI) cases [4,5].

Nevertheless, heterogeneity in hospital resource allocation, service provision and staffing levels across weekday and weekends can affect the delivery of optimal care, leading to studies of the so-called "weekend-effect" [6–8]. Higher rates of mortality have been indicated in patients admitted to hospital for an acute myocardial infarction (AMI) at a weekend, an evening or holiday period [7,9,10]. However, previous results in AMI patients are inconsistent [11–13]. An analysis of the CRUSADE registry highlighted that, despite weekend admission being associated with a delay in invasive therapy, this did not impact rates of adverse events [13]. Similarly, the Get With the Guidelines-Coronary Artery Disease database showed that despite longer door-to-balloon time, mortality rates were similar between 'out-of-hour' and 'in-hour' hospital admission [11].

Many previous studies have focused on outcome differences across weekend/ weekday admissions in STEMI patients, with limited data for patients with NSTEMI or unstable angina (NSTEMI/ UA). Moreover, service provision and delivery, healthcare systems, deprivation and population demographics vary both temporally and by geography, thereby confounding the analysis of a weekend-effect. Finally, the definition of weekend or "out-of-hours" admission varies between populations. Such factors might explain, in part, the inconsistencies across previous studies. Therefore, the aim of this study was to examine the

For peer review only - http://bmjopen.bmj.com/site/about/guidelines.xhtml

effect of weekend vs. weekday admission on outcomes following presentation with STEMI or NSTEMI/ UA, in a contemporary, multicentre dataset of the UK.

Methods

MINAP Dataset

The Myocardial Ischaemia National Audit Project (MINAP) collects prospective data on the management of heart attacks in the UK [14]. This study analysed data from three contributing centres, namely the Freeman Hospital (Newcastle-upon-Tyne), the Royal Stoke Hospital (Stoke-on-Trent) and the University Hospital of Wales (Cardiff). The dataset includes variables detailing patient characteristics, emergency response/ admission dates, processes of care, and outcomes within hospital discharge. Long-term mortality tracking was available from the Office for National Statistics for English patients and the Welsh Demographic Service for Welsh patients.

Study Design

This retrospective analysis included all patients who had a discharge diagnosis of an ACS and were sub-classified into ST elevation myocardial infarction, non-ST elevation myocardial infarction, or unstable angina.

Using hospital admission dates, patients were classified into weekend or weekday admission (reference group). In line with previous studies, weekends were defined as any admission between 12:00am Saturday and 11:59pm Sunday, with weekday defined as any admissions outside this time window [7–9]. The primary outcome measures were in-hospital mortality, 30-day mortality and long-term survival. Secondary outcomes included the following process of care indicators: admission to a cardiology ward, length of stay (defined as whole number of days between admission date and discharge date), performance of coronary angiography/ echocardiography, time to coronary angiography (defined as number

For peer review only - http://bmjopen.bmj.com/site/about/guidelines.xhtml

of hours between admission time and time of angiogram), and discharged on beta-blocker, ACE-I or statins. Note that any patient admitted and discharged on the same day had length of stay defined as zero. All the outcomes were analysed as a whole cohort (with adjustment for STEMI indication) and separately in STEMI and NSTEMI/ UA subgroups.

Statistical Analysis

For descriptive analysis, continuous variables were presented as means with standard deviations and compared using the t-test. Categorical variables were presented as frequencies of occurrence and compared using the chi-squared test. Patients with missing endpoint variables were excluded from the analysis of that endpoint. Other patient characteristic variables that were missing were assigned to the gender-matched median value for continuous variables or the reference category for categorical variables.

To control for potential differences in baseline covariates between weekend and weekday admission, propensity scores (PS) for being admitted at a weekend were calculated for each patient [15,16]. Although day of hospital admission is not a modifiable intervention, one could regard this in the context of differences in resource allocation between the weekend and weekday. Variables included in the logistic regression model to calculate the PS included all those given in **Table 1** and an admitting centre indicator. Patients were stratified into five strata using their PS, with the cut-off values determined by the quintiles of the PS distribution for all patients. Such a method has been shown to remove 90% of the bias due to the covariates within the PS model [15,16].

Within each PS strata, binary outcomes were compared directly between admission day groups, with the odds ratios (ORs) pooled by the Mantel-Haenszel method [17]. For time-to-event outcomes (length of stay, time-to-angiography and long-term survival), the unadjusted survival curves were obtained using the Kaplan-Meier estimate and adjusted hazards ratios (HRs) were calculated using a Cox proportional hazards model. The Cox

For peer review only - http://bmjopen.bmj.com/site/about/guidelines.xhtml

proportional hazards model included weekend indication as the covariate and was stratified by PS strata, thereby allowing the baseline hazard to vary across strata. The proportional hazards assumption of the weekend effect variable was checked by examining the Schoenfeld residuals against time; weekend-by-time interactions were included where necessary. We did not model length of stay beyond 50 days, or time-to-angiography beyond 7 days for NSTEMI/ UA and beyond 5 hours for STEMI.

R version 3.3.1 [18] was used for all statistical analyses. Graphical plots where made using the "ggplot2" package [19], and the "survival" package was used for time-to-event analyses [20,21].

Results

There were 18,166 admissions across the three centres between 1st January 2010 and 30th March 2016. This comprised 3,140 admissions in University Hospital of Wales, 7,023 admissions in Royal Stoke Hospital and 8,003 admissions in Freeman Hospital. Together, 461 admissions did not meet the discharge diagnosis criteria. Thus, the analysis sample size was 17,705 with n=9,322 admissions for a STEMI and n=7,887 admissions for an NSTEMI/ UA; the type of ACS was unknown in the remaining 496 records, which were removed from the STEMI or NSTEMI/ UA subgroup analysis.

Table 1 gives the patient baseline characteristics by weekend or weekday admission. Patients admitted on a weekday were older (p=0.001), were more likely female (p=0.028), and had higher rates of previous AMI (p=0.011), previous angina (p=0.002), cerebrovascular disease (p=0.010), asthma/ COPD (p=0.044) and previous PCI (p=0.014). Conversely, weekend patients were more likely to have elevated enzymes (p<0.001) or present with ST elevation (p<0.001), although Killip class did not significantly differ between admission groups. Importantly, rates of admission to a cardiologist were not different across weekend or

For peer review only - http://bmjopen.bmj.com/site/about/guidelines.xhtml

weekday cohorts, with this being over 96% for both weekday and weekend admissions (p=0.216).

Mortality

Figure 1 shows hospital mortality rates by day of the week. There was no apparent trend in either overall hospital mortality or cardiac/ non-cardiac related deaths. In the whole cohort, the observed hospital mortality rate was 3.96% and 3.41% for weekday and weekend admission groups, respectively (OR 0.86, 95% CI: 0.71, 1.03) (**Table 2**). After PS stratification, the pooled OR was 0.82 (95% CI: 0.58, 1.09). Similar findings were found across STEMI and NSTEMI/ UA subgroups (**Table 3**).

Overall one-, three- and five-year survival rates were 89.7%, 82.3% and 74.9%, respectively. As a whole cohort, there was no significant difference in the Kaplan-Meier survival curves between admission groups (log-rank test, p=0.569) (**Figure 2**). The PS adjusted HR from a Cox proportional hazards model for the whole cohort was 0.99 (95% CI: 0.90, 1.09) for weekend over weekday admission. In STEMI cases, the Kaplan-Meier curves were significantly different (log-rank test, p=0.032), but this was not observed for NSTEMI/ UA cases (log-rank test, p=0.103). After PS adjustment, long-term survival was not significantly different between weekday or weekend admissions, with a HR for STEMI cases of 0.94 (95% CI: 0.82, 1.07) and for NSTEMI/ UA cases of 1.02 (95% CI: 0.89, 1.18).

Admission Process

In the weekend group, 4045 of 4318 (93.7%) patients were admitted to either a cardiac ward or a cardiac care unit (CCU), compared with 12548 of 13345 (94.0%) in the weekday group (**Table 2**). Rates of admission to a cardiac ward or CCU were not significantly different between the weekend and weekday, with an adjusted OR of 0.97 (95%

For peer review only - http://bmjopen.bmj.com/site/about/guidelines.xhtml

CI: 0.76, 1.23). In STEMI admissions the adjusted OR was 1.08 (95% CI: 0.67, 1.52) and in NSTEMI/ UA admissions was 0.93 (95% CI: 0.71, 1.20) (**Table 3**).

There was a significant difference in the Kaplan-Meier curves for length of stay between weekday and weekend admission for STEMI (log-rank p=0.001) and for NSTEMI/ UA patients (log-rank p<0.001). A time-dependent Cox proportional hazards model indicated that patients admitted during the weekend were significantly less likely to be discharged within one day (HR 0.72, 95% CI: 0.66, 0.78), but more likely to be discharged between 1 and 4 days (HR 1.13, 95% CI: 1.08, 1.18). For instance, 21.7% of weekday admission patients were discharged within one day, compared with 14.7% at the weekend. After 4 days, the length of stay between weekday and weekend admission was comparable (**Table 4**). Similar findings were found when examining length of stay across STEMI and NSTEMI/ UA subgroups (**Supplementary Table 1**).

Coronary Angiography

There was no significant difference in the proportion of patients receiving a coronary angiography between weekday and weekend admission (OR 0.91, 95% CI: 0.71, 1.13) (**Table 2**). In STEMI cases, 96.5% and 96.6% of patients underwent an angiography on a weekday and weekend, respectively (adjusted OR 0.95, 95% CI: 0.62, 1.36). Similarly, NSTEMI/ UA patients did not have significantly different odds of undergoing a coronary angiography by weekend admission, after PS stratification adjustment (**Table 3**).

Within the group of patients who underwent a coronary angiography, we assessed the time-to-angiography where time of the angiogram was also available (n=7,284 STEMI and n=5,705 NSTEMI/ UA). There was a significant difference in the cumulative event rates for time-to-angiography for NSTEMI/ UA (log-rank p<0.001), and for STEMI cases (log-rank p=0.01) (**Figure 3**). In NSTEMI/ UA cases, the number of patients receiving angiography within 24 hours differed significantly by weekend admission groups, with similar findings at

For peer review only - http://bmjopen.bmj.com/site/about/guidelines.xhtml

72 hours (**Supplementary Table 2**). For instance, 88.9% of weekend NSTEMI/ UA admissions received an angiography within 72 hours, compared with 91.1% of weekday NSTEMI/ UA admissions (p=0.028). After PS adjustment, the time dependent Cox proportional hazards model indicated that weekend NSTEMI/ UA patients were less likely to undergo a coronary angiography within 2 hours (HR 0.54, 95% CI: 0.42, 0.69) and between 2-24 hours (HR 0.72, 95% CI: 0.66, 0.79), with the observed mean time-to-angiography being approximately 3 hours longer for the weekend cohort by 24 hours (**Table 5**). Baseline NSTEMI/ UA patient characteristics between those undergoing angiography within 24 hours and those after 24 hours are given in **Supplementary Table 3**. NSTEMI/ UA patients who underwent angiography within 24 hours were more likely to have an ACS admission diagnosis (p<0.001), previous angina (p=0.024), hypercholesterolemia (p<0.001) or a family history of coronary heart disease (p<0.001), and were significantly more likely to be admitted to a cardiologist (p<0.001) compared with those over 24 hours.

In STEMI patients, after PS adjustment the Cox proportional hazards model indicated that there was a slight delay for weekend admission within 1 hour (HR 0.89, 95% CI: 0.84, 0.94) (**Table 5**). Specifically, between 0 and 1 hour, the observed mean time-to-angiography was 0.55 hours and 0.57 hours for weekday and weekend admission, respectively. After 1 hour, there was no significant difference in time-to-angiography between weekend or weekday STEMI admissions after PS adjustment (**Table 5**).

Echocardiography

At the weekend, 54.9% of patients underwent an echocardiogram, compared with 51.3% of weekday admissions, with both the unadjusted and PS adjusted ORs being significantly different from one (**Table 2**). Specifically, the pooled OR from the PS stratification was 1.16 (95% CI: 1.01, 1.31), indicating that patients admitted over the

For peer review only - http://bmjopen.bmj.com/site/about/guidelines.xhtml

weekend had 16% greater odds of undergoing an echocardiography compared with those admitted during the week.

Prescribed Medication on discharge

The unadjusted analysis of both prescription of beta-blocker on discharge and prescription of ACE-I on discharge indicated significantly increased odds for those admitted at the weekend (**Table 2**). However, these findings were not significant after adjusting for PS. The crude proportions of statins on discharge were similar across the two groups, at 95.5% for both weekday and weekend admission (adjusted OR 0.96, 95% CI: 0.68, 1.24).

Discussion

The findings of the current study can be summarised as follows: 1) in current UK practice, patients admitted to 3 tertiary cardiac centres during weekends do not present with a higher baseline risk than weekday patients; 2) there was no difference in short- or long-term survival between weekends and weekday groups; 3) for NSTEMI patients there was a delay in angiography for patients admitted at a weekend; 4) other metrics of care quality such as admission under a cardiologist, performance of echocardiography and discharge medications were not adversely affected by a weekend admission.

The lack of a weekend effect in the treatment of an ST elevation myocardial infarction in a contemporary UK cohort is not surprising. The most recently published MINAP data for 2013/14 demonstrates that for the whole of the UK, 98.5% of patients with ST elevation received primary PCI, with few patients receiving thrombolysis [22]. Amongst its many benefits, with respect to day of the week, a primary PCI pathway is a consultant-led and consultant-delivered service, which is undertaken regardless of the time of day or day of the week. Therefore, STEMI treatment in the UK is treated on a 24/7 basis and unless the

For peer review only - http://bmjopen.bmj.com/site/about/guidelines.xhtml

patients admitted at a weekend are of a higher-risk profile, then it would not be expected that a weekend admission would be disadvantageous to their outcomes.

However, pathways for the treatment of NSTEMI/ UA are markedly different in the UK to those for STEMI. The findings of the current study identify differences in patient treatment with respect to their timings, but not regarding patient survival. The lack of a mortality difference for a weekend NSTEMI admission may, in part, be due to the high percentage of cases admitted under the care of a cardiologist regardless of day of admission (>96% for both cohorts). Consequently, the patients may well have received optimal care in a timely fashion. Crucially, review by a cardiologist early in a patient's admission can identify patients who are unstable or deemed to be high-risk NSTEMI patients and these can be prioritised and fast-tracked to the catheter-lab along a pathway similar to primary PCI. Early identification of high-risk NSTEMI patients by the admitting cardiologist and performing coronary angiography within 2 hours, regardless of the day of the week, can ensure that this risk is ameliorated by invasive management [23]. Indeed, several UK centres have developed direct transfer protocols to reduce time-to-angiography for patients with NSTEMI [24]. The optimal timing of angiography is less clear for intermediate-risk NSTEMI cases, with a lack of consistency in published trials [25–27]. In a recent meta-analysis of 10 time-to-angiography trials, there was no difference in mortality between patients receiving angiography early (within 24-hours) or late (24-86 hours) [28]. Therefore, although the ESC guidelines suggest that the high-risk cohort receive angiography within 24 hours, they recommend that the intermediate-risk cohort receive angiography within 72 hours [5]. In the current study, the proportion of NSTEMI/ UA patients receiving angiography within 24 and 72 hours was significantly lower for weekend admissions compared with weekday admissions.

Although the current study does not demonstrate a weekend-effect on patient outcomes, it does demonstrate an effect on length of stay, which is increasingly used as an

For peer review only - http://bmjopen.bmj.com/site/about/guidelines.xhtml

endpoint to monitor quality of service. The HAC-X group demonstrated that a direct transfer protocol for angiography in NSTEMI patients reduced the length of stay by six days per admission [24]. Therefore, although 7 day working (and with it angiography) may not improve patient survival after an ACS, it is likely to significantly reduce their length of stay. A prolonged hospital admission has been closely associated with iatrogenic complications such as infection and venous thrombosis [29,30]. Therefore, as well as having significant implications on bed utilisation, efficiency and costs, performing angiography on NSTEMI patients at the weekend is likely to improve patients' experience and reduce their potential hospital acquired morbidity. Speculatively, the longer length of stay for weekend admissions might explain the finding of higher rates of echocardiograms performed at weekends.

Several factors might explain the differences in the results of previous studies with the current one. Firstly, the data analysed in the current study is derived from the period 2010-2016 and hence reflects contemporary ACS management. Current medical practice has evolved greatly over the last decade. Therefore, the findings of older studies will not necessarily be expected to be relevant or similar to a more contemporary cohort [6–8]. Secondly, the three centres involved in the current study are large university teaching hospitals with a daily review of patients by a senior cardiologist, and 24/7 access to echocardiography and cardiac catheterisation. Therefore, data that includes significant numbers of admissions to smaller hospitals without 24/7 cardiology services might lead to differing outcomes for patients admitted during the weekends compared to a weekday. Finally, differing service provision, patient populations, demographics and healthcare systems might also lead to conflicting results of previous studies with the current study.

The strengths of the current study are that it is an analysis of contemporary practice, the data is derived from a robust national database, and the three centres involved are large tertiary interventional centres with high numbers of patients. However, as with any

For peer review only - http://bmjopen.bmj.com/site/about/guidelines.xhtml

retrospective analysis there are limitations and the robustness of the conclusions are directly related to the quality of data entered. Unmeasured confounders inherent in observational studies of this nature potentially influence any conclusions. Finally, as discussed above, any weekend-effect in ACS patients will largely be affected by the patient demographics and their baseline risk, the healthcare system and the hospitals involved. Therefore, our findings might not generalise to other hospitals or healthcare systems.

Conclusions

In regional, university cardiac centres, with specialist cardiology services in the UK, ACS patients had similar mortality and process of care when admitted at a weekend compared with a weekday. Delays to time-to-angiography occurred in NSTEMI/ UA patients admitted at the weekend compared to weekdays, but this did not influence survival.

Funding Statement

This work was funded by the Medical Research Council through the Health e-Research Centre, University of Manchester [MR/K006665/1] and a grant through the North Staffordshire Heart Committee.

Competing Interests Statement

The authors have no conflict of interest.

Author Contributions

TM, AGZ, MAM contributed to the acquisition of the data for the analysis. GPM, TK, MS and MAM made substantial contributions to the concept of the work in addition to performing the analysis. GPM and TK drafted the initial version of the manuscript. GPM, TK, MS, RA, AG, AJ, CSK, DB, GH, AGZ and MAM interpreted the results, revised the

For peer review only - http://bmjopen.bmj.com/site/about/guidelines.xhtml

paper critically for important intellectual content and approved the final version of the paper. All authors agreed to be accountable for all aspects of the work.

Data sharing statement

The statistical code used to analyse the data is available upon reasonable request by emailing MAM (corresponding author).

For peer review only

For peer review only - http://bmjopen.bmj.com/site/about/guidelines.xhtml

Figure Legends

Figure 1: In-hospital mortality rates by the day of arrival decomposed into cardiac and non-cardiac related mortality.

Figure 2: Long-term survival following weekend versus weekday admission as a whole cohort and across STEMI and NSTEMI/ UA subgroups.

Figure 3: Cumulative event rate for time to coronary angiography following weekend versus weekday admission across STEMI and NSTEMI/ UA subgroups.

For peer review only

For peer review only - http://bmjopen.bmj.com/site/about/guidelines.xhtml

Tables

Table 1: Baseline Summary

Variable	Whole Cohort [n=17705]	Weekday Admission [n=13378]	Weekend Admission [n=4327]	p-value	Missing (% of whole cohort)
Age at Admission, mean (SD)	66.0 (13.0)	66.2 (13.0)	65.5 (13.1)	0.001	7 (0.04)
Male, n (%)	12530 (70.8)	9410 (70.0)	3120 (72.3)	0.028	0 (0.00)
Caucasian, n (%)	12283 (69.4)	9360 (70.0)	2923 (67.6)	0.003	4988 (28.2)
Admission Diagnosis					0 (0.00)
Acute coronary syndrome, n (%)	15427 (87.1)	11689 (87.4)	3738 (86.4)	0.097	
Chest pain unknown cause, n (%)	1759 (9.9)	1316 (9.84)	443 (10.2)	0.461	
Other, n (%)	519 (2.9)	373 (2.79)	146 (3.37)	0.053	
Previous AMI, n (%)	3768 (21.3)	2907 (21.7)	861 (19.9)	0.011	109 (0.62)
Previous Angina, n (%)	3968 (22.4)	3073 (23.0)	895 (20.7)	0.002	282 (1.59)

For peer review only - http://bmjopen.bmj.com/site/about/guidelines.xhtml

Hypertension, n (%)	9392 (53.0)	7153 (53.5)	2239 (51.7)	0.050	255 (1.44)
Hypercholesterolemia, n (%)	7166 (40.5)	5458 (40.8)	1708 (39.5)	0.127	574 (3.24)
PVD, n (%)	809 (4.57)	626 (4.68)	183 (4.23)	0.234	85 (0.48)
Cerebrovascular Disease, n (%)	1270 (7.17)	998 (7.46)	272 (6.29)	0.010	83 (0.47)
Asthma or COPD, n (%)	2717 (15.3)	2095 (15.7)	622 (14.4)	0.044	114 (0.64)
Chronic Renal Failure, n (%)	691 (3.90)	527 (3.94)	164 (3.79)	0.693	143 (0.81)
Heart Failure, n (%)	508 (2.87)	379 (2.83)	129 (2.98)	0.649	159 (0.90)
Enzymes Elevated, n (%)	16378 (92.5)	12304 (92.0)	4074 (94.2)	<0.001	634 (3.58)
Previous/ Current Smoker, n (%)	11892 (67.2)	9021 (67.4)	2871 (66.4)	0.195	550 (3.11)
Cholesterol, mean (SD)	4.77 (1.35)	4.76 (1.35)	4.82 (1.38)	0.015	4590 (25.9)
Diabetes					126 (0.71)
Dietary control, n (%)	609 (3.44)	464 (3.47)	145 (3.35)	0.749	
Oral medicine, n (%)	1870 (10.6)	1394 (10.4)	476 (11.0)	0.293	
Insulin, n (%)	721 (4.07)	571 (4.27)	150 (3.47)	0.023	
Insulin and medication, n (%)	297 (1.68)	224 (1.67)	73 (1.69)	0.999	
Previous PCI, n (%)	2124 (12.0)	1651 (12.3)	473 (10.9)	0.014	17 (0.10)

For peer review only - http://bmjopen.bmj.com/site/about/guidelines.xhtml

Previous CABG, n (%)	959 (5.42)	732 (5.47)	227 (5.25)	0.595	4 (0.02)
Systolic BP, mean (SD)	135.3 (27.7)	135.1 (27.5)	135.8 (28.3)	0.219	2574 (14.5)
Heart Rate, mean (SD)	76.8 (19.3)	76.5 (19.1)	77.5 (19.8)	0.006	2257 (12.7)
Admitting Consultant					52 (0.29)
Cardiologist, n (%)	17093 (96.5)	12929 (96.6)	4164 (96.2)	0.216	
Other general physician, n (%)	445 (2.51)	325 (2.43)	120 (2.77)	0.230	
Other, n (%)	115 (0.65)	88 (0.66)	27 (0.62)	0.895	
Beta Blocker, n (%)	4670 (26.4)	3555 (26.6)	1115 (25.8)	0.306	617 (3.48)
Statin, n (%)	6997 (39.5)	5360 (40.1)	1637 (37.8)	0.009	630 (3.56)
Glucose, mean (SD)	8.60 (4.60)	8.55 (4.61)	8.76 (4.58)	0.015	3044 (17.2)
Height, mean (SD)	170.1 (9.76)	170.0 (9.81)	170.5 (9.57)	0.011	3514 (19.8)
Weight, mean (SD)	81.9 (18.7)	81.8 (18.8)	82.0 (18.3)	0.624	2571 (14.5)
Family History of CHD, n (%)	8287 (46.8)	6273 (46.9)	2014 (46.5)	0.705	1939 (11.0)
Creatinine, mean (SD)	95.7 (64.6)	95.5 (63.3)	96.3 (68.4)	0.502	266 (1.50)
Haemoglobin, mean (SD)	136.6 (19.1)	136.3 (19.2)	137.5 (18.8)	<0.001	195 (1.10)

For peer review only - http://bmjopen.bmj.com/site/about/guidelines.xhtml

Killip class					6919 (39.1)
No evidence of Heart Failure, n (%)	9103 (51.4)	6918 (51.7)	2185 (50.5)	0.170	
Basal Crepitations, n (%)	811 (4.58)	590 (4.41)	221 (5.11)	0.062	
Pulmonary Oedema, n (%)	600 (3.39)	453 (3.39)	147 (3.40)	0.999	
Cardiogenic Shock, n (%)	266 (1.50)	195 (1.46)	71 (1.64)	0.430	
Cardiac Arrest, n (%)	1493 (8.43)	1106 (8.27)	387 (8.94)	0.174	0 (0.00)
Thienopyridene, n (%)	15632 (88.3)	11774 (88.0)	3858 (89.2)	0.043	109 (0.62)
STEMI, n (%)	9322 (52.7)	6781 (50.7)	2541 (58.7)	<0.001	496 (2.80)

AMI: Acute Myocardial Infarction; BP: Blood Pressure; CABG: Coronary Artery Bypass Graft; CHD: Coronary Heart Disease; COPD: Chronic Obstructive Pulmonary Disease; LVEF: Left Ventricular Ejection Fraction; MI: Myocardial Infarction; PCI: Percutaneous Coronary Intervention; PVD: Peripheral Vascular Disease; STEMI: ST Segment Elevation Myocardial Infarction

For peer review only - http://bmjopen.bmj.com/site/about/guidelines.xhtml

Table 2: Event numbers, crude odds ratios (OR) and pooled OR across PS stratification strata. Bold item indicate significant effects at the 5% level.

**Outcome**	**Weekday Admission [n=13378]**	**Weekend Admission [n=4327]**	**Crude OR (95% CI)**	**PS Adjusted OR (95% CI)**
Hospital Mortality	529/13354 (3.96%)	147/4312 (3.41%)	0.86 (0.71, 1.03)	0.82 (0.58, 1.09)
30-day Mortality	553/10874 (5.09%)	182/3545 (5.13%)	1.01 (0.85, 1.20)	0.96 (0.72, 1.28)
Admission to Cardiology Ward	12548/13345 (94.0%)	4045/4318 (93.7)	0.94 (0.82, 1.09)	0.97 (0.76, 1.23)
Coronary Angiography	12401/13265 (93.5%)	3987/4288 (93.0%)	0.92 (0.81, 1.06)	0.91 (0.71, 1.13)
Echocardiography	5772/11262 (51.3%)	2014/3666 (54.9%)	**1.16 (1.08, 1.25)**	**1.16 (1.01, 1.31)**
Discharged on Beta-blocker	10320/12181 (84.7%)	3404/3943 (86.3%)	**1.14 (1.03, 1.26)**	1.06 (0.88, 1.26)
Discharged on ACE-I	10801/12109 (89.2%)	3552/3930 (90.4%)	**1.14 (1.01, 1.29)**	1.09 (0.88, 1.34)
Discharged on Statins	11590/12131 (95.5%)	3764/3942 (95.5%)	0.99 (0.83, 1.18)	0.96 (0.68, 1.24)

For peer review only - http://bmjopen.bmj.com/site/about/guidelines.xhtml

Table 3: Event numbers and pooled odds ratios (OR) across PS stratification strata by STEMI and NSTEMI/ UA subgroups.

	STEMI (n=9322)			NSTEMI/ UA (n=7887)		
**Outcome**	**Weekday Admission [n=6781]**	**Weekend Admission [n=2541]**	**PS Adjusted OR (95% CI)**	**Weekday Admission [n=6190]**	**Weekend Admission [n=1697]**	**PS Adjusted OR (95% CI)**
Hospital Mortality	379/6774 (5.59%)	110/2533 (4.34%)	0.83 (0.58, 1.15)	137/6175 (2.22%)	36/1691 (2.13%)	0.82 (0.45, 1.38)
30-day Mortality	379/5633 (6.73%)	132/2123 (6.22%)	0.99 (0.69, 1.35)	157/4913 (3.20%)	45/1352 (3.33%)	0.88 (0.53, 1.41)
Admission to Cardiology Ward	6486/6763 (95.9%)	2442/2533 (96.4%)	1.08 (0.67, 1.52)	5673/6176 (91.9%)	1518/1696 (89.5%)	0.93 (0.71, 1.20)
Coronary Angiography	6512/6749 (96.5%)	2445/2530 (96.6%)	0.95 (0.62, 1.36)	5532/6113 (90.5%)	1465/1669 (87.8%)	0.92 (0.70, 1.15)
Echocardiography	3241/6085 (53.3%)	1267/2210 (57.3%)	1.17 (0.98, 1.38)	2377/4796 (49.6%)	714/1374 (52.0%)	1.16 (0.93, 1.42)
Discharged on Beta-blocker	5226/5976 (87.4%)	2018/2261 (89.3%)	1.08 (0.83, 1.39)	4838/5846 (82.8%)	1328/1600 (83.0%)	1.03 (0.80, 1.32)

For peer review only - http://bmjopen.bmj.com/site/about/guidelines.xhtml

Discharged on ACE-I	5529/5940 (93.1%)	2109/2254 (93.6%)	1.04 (0.72, 1.41)	4998/5815 (86.0%)	1374/1594 (86.2%)	1.13 (0.83, 1.41)
Discharged on Statins	5772/5951 (97.0%)	2187/2260 (96.8%)	0.89 (0.50, 1.32)	5481/5824 (94.1%)	1498/1600 (93.6%)	1.01 (0.68, 1.41)

For peer review only - http://bmjopen.bmj.com/site/about/guidelines.xhtml

Table 4: Number of patients discharged within each time-window and PS adjusted hazard ratios (HR) from the time-dependent Cox proportional hazards models for length of stay, as a whole cohort. Bold items indicate significant effects at the 5% level.

Length of stay	Discharged, n (%) Weekday Cohort	Discharged, n (%) Weekend Cohort	HR (95% CI)
0 – 1 day	2888 (21.7%)	636 (14.7%)	**0.72 (0.66, 0.78)**
1 - 4 days	6731 (64.5%)	2595 (70.5%)	**1.13 (1.08, 1.18)**
4 - 50 days	3639 (98.2%)	1063 (98.1%)	0.97 (0.90, 1.04)

For peer review only

For peer review only - http://bmjopen.bmj.com/site/about/guidelines.xhtml

Table 5: PS adjusted hazard ratios (HR) from time-dependent Cox proportional hazards models for time to coronary angiography in STEMI and NSTEMI/ UA patients. Bold items indicate significant effects at the 5% level.

	STEMI		
**Time to coronary angiography**	**Mean time-to-angiography Weekday, (hours)**	**Mean time-to-angiography Weekend, (hours)**	**PS adjusted HR (95% CI)**
0 - 1 hour	0.55	0.57	**0.89 (0.84, 0.94)**
1 - 2 hours	1.52	1.53	0.99 (0.87, 1.11)
2 – 5 hours	3.27	3.11	1.25 (1.00, 1.56)
	**NSTEMI/ UA**		
0 - 2 hours	1.89	1.94	**0.54 (0.42, 0.69)**
2 - 24 hours	15.7	18.3	**0.71 (0.65, 0.77)**
24 hours - 4 days	56.2	53.9	**1.22 (1.09, 1.36)**
4 - 7 days	139.9	137.0	0.94 (0.68, 1.29)

For peer review only

For peer review only - http://bmjopen.bmj.com/site/about/guidelines.xhtml

References

1. Berger PB, Ellis SG, Holmes DRJ, Granger CB, Criger DA, Betriu A, Topol EJ, Califf RM. Relationship between delay in performing direct coronary angioplasty and early clinical outcome in patients with acute myocardial infarction: results from the global use of strategies to open occluded arteries in Acute Coronary Syndromes (GUSTO-IIb) trial. *Circulation*. 1999;100:14–20.

2. McNamara RL, Wang Y, Herrin J, Curtis JP, Bradley EH, Magid DJ, Peterson ED, Blaney M, Frederick PD, Krumholz HM. Effect of Door-to-Balloon Time on Mortality in Patients With ST-Segment Elevation Myocardial Infarction. *J Am Coll Cardiol*. 2006;47:2180–2186.

3. O'Gara PT, Kushner FG, Ascheim DD, Casey DE, Chung MK, de Lemos JA, Ettinger SM, Fang JC, Fesmire FM, Franklin BA, Granger CB, Krumholz HM, Linderbaum JA, Morrow DA, Newby LK, Ornato JP, Ou N, Radford MJ, Tamis-Holland JE, Tommaso CL, Tracy CM, Woo YJ, Zhao DX. 2013 ACCF/AHA Guideline for the Management of ST-Elevation Myocardial Infarction. *J Am Coll Cardiol*. 2013;61:e78–e140.

4. Amsterdam EA, Wenger NK, Brindis RG, Casey DE, Ganiats TG, Holmes DR, Jaffe AS, Jneid H, Kelly RF, Kontos MC, Levine GN, Liebson PR, Mukherjee D, Peterson ED, Sabatine MS, Smalling RW, Zieman SJ. 2014 AHA/acc guideline for the management of patients with Non-ST-Elevation acute coronary syndromes: A report of the American College of Cardiology/American Heart Association Task Force on Practice Guidelines. *J Am Coll Cardiol*. 2014;64:e139–e228.

5. Roffi M, Patrono C, Collet J-P, Mueller C, Valgimigli M, Andreotti F, Bax JJ, Borger MA, Brotons C, Chew DP, Gencer B, Hasenfuss G, Kjeldsen K, Lancellotti P, Landmesser U, Mehilli J, Mukherjee D, Storey RF, Windecker S, Baumgartner H,

For peer review only - http://bmjopen.bmj.com/site/about/guidelines.xhtml

Gaemperli O, Achenbach S, Agewall S, Badimon L, Baigent C, Bueno H, Bugiardini R, Carerj S, Casselman F, Cuisset T, Erol Ç, Fitzsimons D, Halle M, Hamm C, Hildick-Smith D, Huber K, Iliodromitis E, James S, Lewis BS, Lip GYH, Piepoli MF, Richter D, Rosemann T, Sechtem U, Steg PG, Vrints C, Luis Zamorano J. 2015 ESC Guidelines for the management of acute coronary syndromes in patients presenting without persistent ST-segment elevation. *Eur Heart J*. 2016;37:267–315.

6. Bell CM, Redelmeier DA. Mortality among Patients Admitted to Hospitals on Weekends as Compared with Weekdays. *N Engl J Med*. 2001;345:663–668.

7. Kostis WJ, Demissie K, Marcella SW, Shao Y-H, Wilson AC, Moreyra AE. Weekend versus Weekday Admission and Mortality from Myocardial Infarction. *N Engl J Med*. 2007;356:1099–1109.

8. Cram P, Hillis SL, Barnett M, Rosenthal GE. Effects of weekend admission and hospital teaching status on in-hospital mortality. *Am J Med*. 2004;117:151–157.

9. Khoshchehreh M, Groves EM, Tehrani D, Amin A, Patel PM, Malik S. Changes in mortality on weekend versus weekday admissions for Acute Coronary Syndrome in the United States over the past decade. *Int J Cardiol*. 2016;210:164–172.

10. Sorita A, Ahmed A, Starr SR, Thompson KM, Reed DA, Prokop L, Shah ND, Murad MH, Ting HH. Off-hour presentation and outcomes in patients with acute myocardial infarction: systematic review and meta-analysis. *BMJ*. 2014;348:f7393–f7393.

11. Jneid H, Fonarow GC, Cannon CP, Palacios IF, Kilic T, Moukarbel G V., Maree AO, LaBresh KA, Liang L, Newby LK, Fletcher G, Wexler L, Peterson E. Impact of Time of Presentation on the Care and Outcomes of Acute Myocardial Infarction. *Circulation*. 2008;117:2502–2509.

12. Berger A, Stauffer J-C, Radovanovic D, Urban P, Bertel O, Erne P. Comparison of In-Hospital Mortality for Acute Myocardial Infarction in Switzerland With Admission

For peer review only

During Routine Duty Hours Versus Admission During Out of Hours (Insight Into the AMIS Plus Registry). *Am J Cardiol*. 2008;101:422–427.

13. Ryan JW, Peterson ED, Chen AY, Roe MT, Ohman EM, Cannon CP, Berger PB, Saucedo JF, DeLong ER, Normand SL, Pollack C V., Cohen DJ. Optimal timing of intervention in non-ST-segment elevation acute coronary syndromes: Insights from the CRUSADE (Can Rapid risk stratification of Unstable angina patients Suppress ADverse outcomes with Early implementation of the ACC/AHA guidelines) regist. *Circulation*. 2005;112:3049–3057.

14. Mamas MA, Kwok CS, Kontopantelis E, Fryer AA, Buchan I, Bachmann MO, Zaman MJ, Myint PK. Relationship Between Anemia and Mortality Outcomes in a National Acute Coronary Syndrome Cohort: Insights From the UK Myocardial Ischemia National Audit Project Registry. *J Am Heart Assoc*. 2016;5:e003348.

15. Rosenbaum PR, Rubin DB. The Central Role of the Propensity Score in Observational Studies for Causal Effects. *Biometrika*. 1983;70:41–55.

16. Rosenbaum PR, Rubin DB. Reducing Bias in Observational Studies Using Subclassification on the Propensity Score. *J Am Stat Assoc*. 1984;79:516–524.

17. Mantel N, Haenszel W. Statistical aspects of the analysis of data from retrospective studies of disease. *J Natl Cancer Inst*. 1959;22:719–48.

18. R Core Team R. R: A Language and Environment for Statistical Computing [Internet]. R Found. Stat. Comput. 2016;Available from: http://www.r-project.org/

19. Wickham H. ggplot2: elegant graphics for data analysis. Springer New York; 2009.

20. Therneau T. A Package for Survival Analysis in S. 2015;Available from: http://cran.r-project.org/package=survival

21. Therneau T, Grambsch P. Modeling Survival Data: Extending the Cox Model. New York: Springer; 2000.

For peer review only - http://bmjopen.bmj.com/site/about/guidelines.xhtml

For peer review only

22. Weston C, Gavalova L, Whittaker T, Van Leeven R. Myocardial Ischaemia National Audit Project: How the NHS cares for patients with heart attack [Internet]. 2014;Available from: https://www.ucl.ac.uk/nicor/audits/minap/documents/annual_reports/minap-public-report-2014

23. de Araujo Goncalves P. TIMI, PURSUIT, and GRACE risk scores: sustained prognostic value and interaction with revascularization in NSTE-ACS. *Eur Heart J*. 2005;26:865–872.

24. Gallagher SM, Lovell MJ, Jones DA, Ferguson E, Ahktar A, Buckhoree Z, Wragg A, Knight CJ, Mathur A, Smith EJ, Cliffe S, Archbold RA, Rothman MT, Jain AK. Does a “direct” transfer protocol reduce time to coronary angiography for patients with non-ST-elevation acute coronary syndromes? A prospective observational study. *BMJ Open*. 2014;4:e005525.

25. Neumann F-J, Kastrati A, Pogatsa-Murray G, Mehilli J, Bollwein H, Bestehorn H-P, Schmitt C, Seyfarth M, Dirschinger J, Schömig A. Evaluation of Prolonged Antithrombotic Pretreatment (“Cooling-Off” Strategy) Before Intervention in Patients With Unstable Coronary Syndromes. *JAMA*. 2003;290:1593–1599.

26. Montalescot G, Cayla G, Collet J-P, Elhadad S, Beygui F, Le Breton H, Choussat R, Leclercq F, Silvain J, Duclos F, Aout M, Dubois-Randé J-L, Barthélémy O, Ducrocq G, Bellemain-Appaix A, Payot L, Steg P, Henry P, Spaulding C, Vicaut E, ABOARD Investigators for the. Immediate vs Delayed Intervention for Acute Coronary Syndromes. *JAMA*. 2009;302:947.

27. van ’t Hof AWJ, de Vries ST, Dambrink J-HE, Miedema K, Suryapranata H, Hoorntje JCA, Gosselink ATM, Zijlstra F, de Boer M-J. A comparison of two invasive strategies in patients with non-ST elevation acute coronary syndromes: results of the

For peer review only - http://bmjopen.bmj.com/site/about/guidelines.xhtml

Early or Late Intervention in unStable Angina (ELISA) pilot study. 2b/3a upstream therapy and acute coronary syndromes. *Eur Heart J*. 2003;24:1401–5.

28. Milasinovic D, Milosevic A, Marinkovic J, Vukcevic V, Ristic A, Asanin M, Stankovic G. Timing of invasive strategy in NSTE-ACS patients and effect on clinical outcomes: A systematic review and meta-analysis of randomized controlled trials. *Atherosclerosis*. 2015;241:48–54.

29. Sbrojavacca R, Pietrangelo A, Fenoglio L, Violi F, Perticone F, Corazza GR. Reducing the risk of hospital admission: A call to action from the Italian Society of Internal Medicine. *Eur J Intern Med*. 2015;26:476–477.

30. Lilly CM, Cody S, Zhao H, Landry K, Baker SP, McIlwaine J, Chandler MW, Irwin RS, University of Massachusetts Memorial Critical Care Operations Group. Hospital mortality, length of stay, and preventable complications among critically ill patients before and after tele-ICU reengineering of critical care processes. *JAMA*. 2011;305:2175–83.

For peer review only

For peer review only - http://bmjopen.bmj.com/site/about/guidelines.xhtml

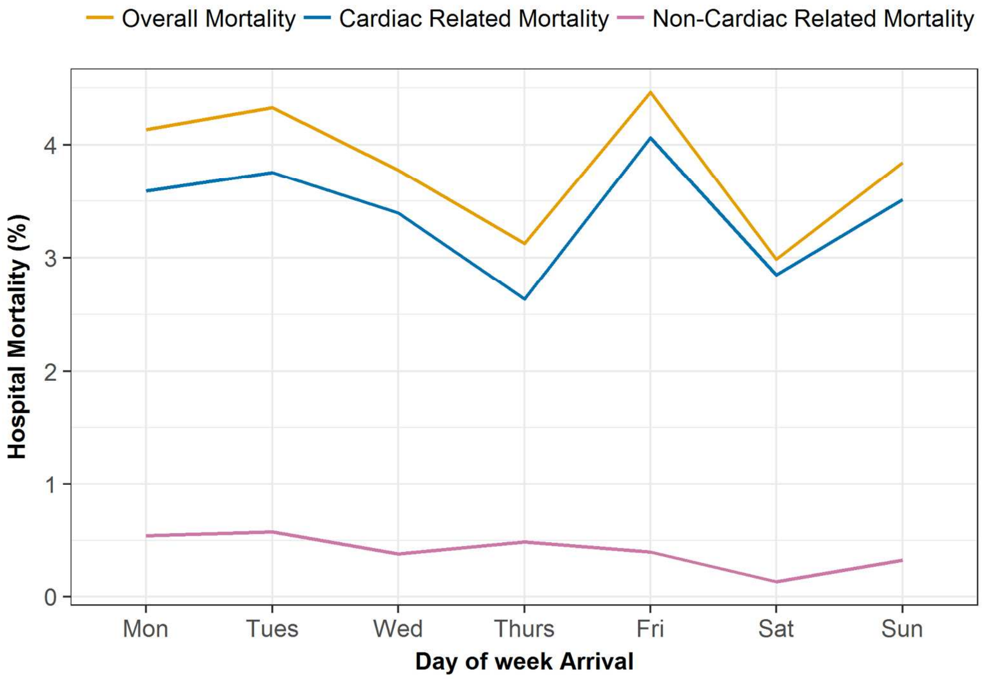

In-hospital mortality rates by the day of arrival decomposed into cardiac and non-cardiac related mortality.

177x127mm (300 x 300 DPI)

view only

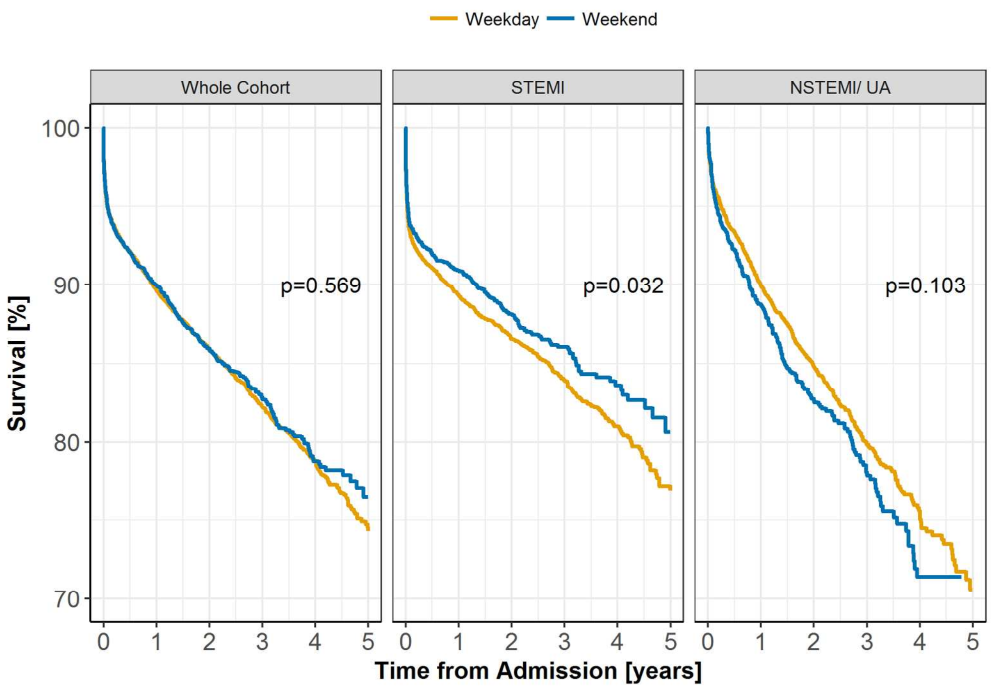

Long-term survival following weekend versus weekday admission as a whole cohort and across STEMI and NSTEMI/ UA subgroups.

177x127mm (300 x 300 DPI)

review only

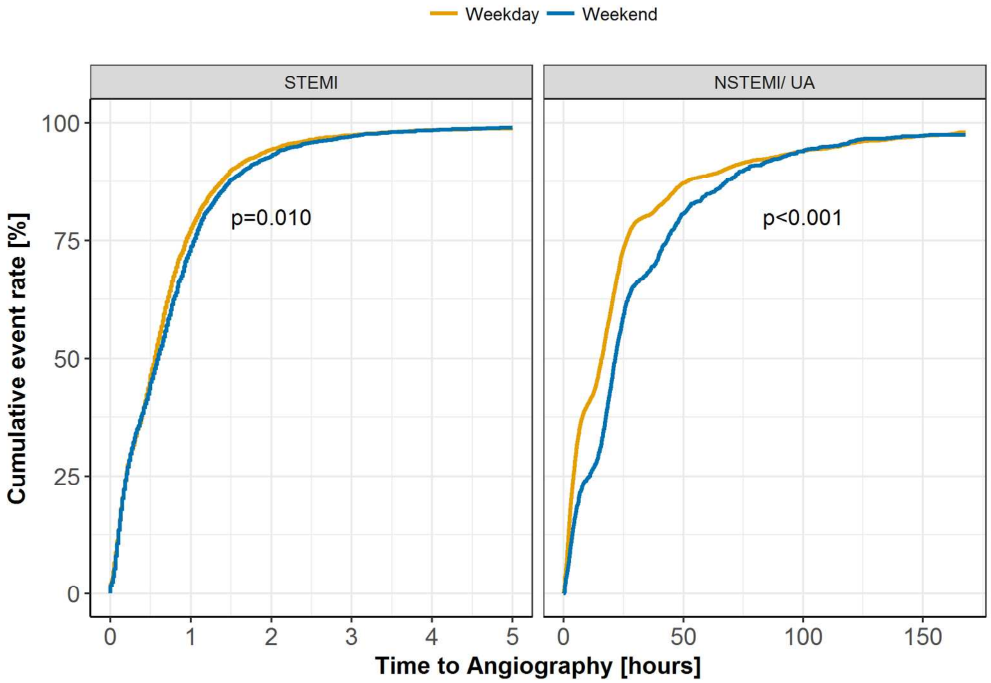

Cumulative event rate for time to coronary angiography following weekend versus weekday admission across STEMI and NSTEMI/ UA subgroups.

177x127mm (300 x 300 DPI)

Supplementary Material

Effect of Weekend Admission on Process of Care and Clinical Outcomes for the Management of Acute Coronary Syndromes

Glen P. Martin*; Tim Kinnaird*; Matthew Sperrin; Richard Anderson; Amr Gamal; Avais Jabbar; Shing Kwok; Diane Barker; Grant Heatlie; Azfar G. Zaman; Mamas A. Mamas

* Joint first authors: these authors contributed equally to manuscript

Supplementary Tables

For peer review only

Supplementary Table 1: Number of patients discharged within each time-window and PS adjusted hazard ratios (HR) from the time-dependent Cox proportional hazards models for length of stay, across STEMI and NSTEMI/ UA subgroups. Bold items indicate significant effects at the 5% level.

	STEMI		
**Length of stay**	**Discharged, n (%) Weekday Cohort**	**Discharged, n (%) Weekend Cohort**	**HR (95% CI)**
0 – 1 day	1074 (15.9%)	352 (13.9%)	**0.85 (0.75, 0.96)**
1 - 4 days	3953 (69.5%)	1659 (76.0%)	**1.13 (1.06, 1.19)**
4 - 50 days	1702 (98.3%)	513 (98.1%)	1.04 (0.94, 1.15)
	**NSTEMI/ UA**		
0 – 1 day	1733 (28.1%)	283 (16.7%)	**0.65 (0.57, 0.74)**
1 - 4 days	2628 (59.3%)	884 (62.7%)	**1.12 (1.04, 1.21)**
4 - 50 days	1770 (98.1%)	515 (97.9%)	0.93 (0.84, 1.03)

For peer review only

Supplementary Table 2: Proportions of NSTEMI/ UA patients receiving angiography within 24 and 72 hours across weekend admission groups.

Time-to-angiography	Weekday NSTEMI/ UA	Weekend NSTEMI/ UA	p-value
Within 24 hours	3242/ 4529 (71.6%)	662/ 1176 (56.2%)	<0.001
Within 72 hours	4125/ 4529 (91.1%)	1046/ 1176 (88.9%)	0.028

For peer review only

Supplementary Table 3: NSTEMI/UA patient characteristics across those that underwent coronary angiography within 24 hours compared with those after 24 hours.

Variable	Angiography≤24 hours [n=3902]	Angiography>24 hours [n=1802]	p-value
Age at Admission, mean (SD)	66.2 (12.4)	67.2 (12.0)	0.004
Male, n (%)	2807 (71.9)	1258 (69.8)	0.109
Caucasian, n (%)	3286 (84.2)	1101 (61.1)	<0.001
Admission Diagnosis			
Acute coronary syndrome, n (%)	3627 (93.0)	1459 (81.0)	<0.001
Chest pain unknown cause, n (%)	253 (6.48)	271 (15.0)	<0.001
Other, n (%)	23 (0.59)	72 (4.00)	<0.001
Previous AMI, n (%)	1169 (30.0)	551 (30.6)	0.659
Previous Angina, n (%)	1555 (39.9)	661 (36.7)	0.024
Hypertension, n (%)	2505 (64.2)	1142 (63.4)	0.567
Hypercholesterolemia, n (%)	2167 (55.5)	877 (48.7)	<0.001
PVD, n (%)	297 (7.61)	164 (9.10)	0.062
Cerebrovascular Disease, n (%)	339 (8.69)	161 (8.93)	0.798
Asthma or COPD, n (%)	647 (16.6)	311 (17.3)	0.550
Chronic Renal Failure, n (%)	126 (3.23)	129 (7.16)	<0.001
Heart Failure, n (%)	147 (3.77)	111 (6.16)	<0.001
Enzymes Elevated, n (%)	3382 (86.7)	1611 (89.4)	0.004
Previous/ Current Smoker, n (%)	2600 (66.6)	1210 (67.1)	0.724
Cholesterol, mean (SD)	4.53 (1.38)	4.48 (1.33)	0.379
Diabetes			
Dietary control, n (%)	157 (4.02)	81 (4.50)	0.449
Oral medicine, n (%)	473 (12.1)	248 (13.8)	0.091
Insulin, n (%)	223 (5.72)	123 (6.83)	0.116
Insulin and medication, n (%)	59 (1.51)	62 (3.44)	<0.001
Previous PCI, n (%)	783 (20.1)	343 (19.0)	0.382
Previous CABG, n (%)	274 (7.02)	192 (10.7)	<0.001
Systolic BP, mean (SD)	136.6 (24.6)	140.3 (25.6)	<0.001
Heart Rate, mean (SD)	71.4 (15.4)	75.8 (18.0)	<0.001
Admitting Consultant			
Cardiologist, n (%)	3893 (99.8)	1708 (94.8)	<0.001
Other general physician, n (%)	4 (0.10)	65 (3.61)	<0.001
Other, n (%)	3 (0.08)	21 (1.17)	<0.001
Beta Blocker, n (%)	1458 (37.4)	713 (39.6)	0.118
Statin, n (%)	2034 (52.1)	988 (54.8)	0.061

For peer review only

Glucose, mean (SD)	7.45 (3.48)	8.16 (4.13)	<0.001
Height, mean (SD)	169.7 (9.90)	169.3 (10.1)	0.251
Weight, mean (SD)	82.7 (17.7)	82.6 (18.5)	0.890
Family History of CHD, n (%)	1942 (49.8)	774 (43.0)	<0.001
Creatinine, mean (SD)	97.6 (54.9)	103.4 (80.4)	0.006
Haemoglobin, mean (SD)	137.4 (18.0)	135.7 (19.6)	0.002
Killip class			
No evidence of Heart Failure, n (%)	3083 (79.0)	1108 (61.5)	<0.001
Basal Crepitations, n (%)	101 (2.59)	172 (9.54)	<0.001
Pulmonary Oedema, n (%)	139 (3.56)	105 (5.83)	<0.001
Cardiogenic Shock, n (%)	14 (0.36)	7 (0.39)	0.999
Cardiac Arrest, n (%)	100 (2.56)	46 (2.55)	0.999
Thienopyridene, n (%)	3384 (86.7)	1662 (92.2)	<0.001

AMI: Acute Myocardial Infarction; BP: Blood Pressure; CABG: Coronary Artery Bypass Graft; CHD: Coronary Heart Disease; COPD: Chronic Obstructive Pulmonary Disease; LVEF: Left Ventricular Ejection Fraction; MI: Myocardial Infarction; PCI: Percutaneous Coronary Intervention; PVD: Peripheral Vascular Disease

For peer review only

STROBE 2007 (v4) checklist of items to be included in reports of observational studies in epidemiology*

Checklist for cohort, case-control, and cross-sectional studies (combined)

Section/Topic	Item #	Recommendation	Reported on page #
**Title and abstract**	1	(*a*) Indicate the study's design with a commonly used term in the title or the abstract	Title page
		(*b*) Provide in the abstract an informative and balanced summary of what was done and what was found	2
**Introduction**			
Background/rationale	2	Explain the scientific background and rationale for the investigation being reported	5
Objectives	3	State specific objectives, including any pre-specified hypotheses	5
**Methods**			
Study design	4	Present key elements of study design early in the paper	6
Setting	5	Describe the setting, locations, and relevant dates, including periods of recruitment, exposure, follow-up, and data collection	6 and 8
Participants	6	(*a*) *Cohort study*—Give the eligibility criteria, and the sources and methods of selection of participants. Describe methods of follow-up<br>*Case-control study*—Give the eligibility criteria, and the sources and methods of case ascertainment and control selection. Give the rationale for the choice of cases and controls<br>*Cross-sectional study*—Give the eligibility criteria, and the sources and methods of selection of participants	6 and 7
		(*b*) *Cohort study*—For matched studies, give matching criteria and number of exposed and unexposed<br>*Case-control study*—For matched studies, give matching criteria and the number of controls per case	N/A
Variables	7	Clearly define all outcomes, exposures, predictors, potential confounders, and effect modifiers. Give diagnostic criteria, if applicable	6 and 7
Data sources/ measurement	8*	For each variable of interest, give sources of data and details of methods of assessment (measurement). Describe comparability of assessment methods if there is more than one group	*6*
Bias	9	Describe any efforts to address potential sources of bias	7
Study size	10	Explain how the study size was arrived at	N/A – registry data
Quantitative variables	11	Explain how quantitative variables were handled in the analyses. If applicable, describe which groupings were chosen and why	7
Statistical methods	12	(*a*) Describe all statistical methods, including those used to control for confounding	7
		(*b*) Describe any methods used to examine subgroups and interactions	7
		(*c*) Explain how missing data were addressed	7
		(*d*) *Cohort study*—If applicable, explain how loss to follow-up was addressed<br>*Case-control study*—If applicable, explain how matching of cases and controls was addressed	N/A

For peer review only

		Cross-sectional study—If applicable, describe analytical methods taking account of sampling strategy	
		(*e*) Describe any sensitivity analyses	N/A
**Results**			
Participants	13*	(a) Report numbers of individuals at each stage of study—eg numbers potentially eligible, examined for eligibility, confirmed eligible, included in the study, completing follow-up, and analysed	8
		(b) Give reasons for non-participation at each stage	8
		(c) Consider use of a flow diagram	N/A
Descriptive data	14*	(a) Give characteristics of study participants (eg demographic, clinical, social) and information on exposures and potential confounders	Table 1
		(b) Indicate number of participants with missing data for each variable of interest	Table 1
		(c) *Cohort study*—Summarise follow-up time (eg, average and total amount)	N/A
Outcome data	15*	*Cohort study*—Report numbers of outcome events or summary measures over time	Table 2 and throughout results section
		Case-control study—Report numbers in each exposure category, or summary measures of exposure	N/A
		Cross-sectional study—Report numbers of outcome events or summary measures	N/a
Main results	16	(*a*) Give unadjusted estimates and, if applicable, confounder-adjusted estimates and their precision (eg, 95% confidence interval). Make clear which confounders were adjusted for and why they were included	Table 2 and page 7
		(*b*) Report category boundaries when continuous variables were categorized	N/A
		(*c*) If relevant, consider translating estimates of relative risk into absolute risk for a meaningful time period	N/A
Other analyses	17	Report other analyses done—eg analyses of subgroups and interactions, and sensitivity analyses	Table 3 and throughout results section
**Discussion**			
Key results	18	Summarise key results with reference to study objectives	12
Limitations	19	Discuss limitations of the study, taking into account sources of potential bias or imprecision. Discuss both direction and magnitude of any potential bias	14 and 15
Interpretation	20	Give a cautious overall interpretation of results considering objectives, limitations, multiplicity of analyses, results from similar studies, and other relevant evidence	15
Generalisability	21	Discuss the generalisability (external validity) of the study results	15
**Other information**			
Funding	22	Give the source of funding and the role of the funders for the present study and, if applicable, for the original study on which the present article is based	Title page

For peer review only

*Give information separately for cases and controls in case-control studies and, if applicable, for exposed and unexposed groups in cohort and cross-sectional studies.
Note: An Explanation and Elaboration article discusses each checklist item and gives methodological background and published examples of transparent reporting. The STROBE checklist is best used in conjunction with this article (freely available on the Web sites of PLoS Medicine at http://www.plosmedicine.org/, Annals of Internal Medicine at http://www.annals.org/, and Epidemiology at http://www.epidem.com/). Information on the STROBE Initiative is available at www.strobe-statement.org.

For peer review only

BMJ Open

Effect of Weekend Admission on Process of Care and Clinical Outcomes for the Management of Acute Coronary Syndromes: a retrospective analysis of three UK centres

Journal:	*BMJ Open*
Manuscript ID	bmjopen-2017-016866.R1
Article Type:	Research
Date Submitted by the Author:	12-Jun-2017

Complete List of Authors:	Martin, Glen; University of Manchester, Health eResearch Centre, Farr Institute<br>Kinnaird, Tim; Keele University, Keele Cardiovascular Research Group; University Hospital of Wales, Department of Cardiology<br>Sperrin, Matthew; University of Manchester, Health eResearch Centre, Farr Institute<br>Anderson, Richard; University Hospital of Wales, Department of Cardiology<br>Gamal, Amr; Newcastle University, Department of Cardiology<br>Jabbar, Avais; Newcastle University, Department of Cardiology<br>Kwok, Chun; Keele University, Keele Cardiovascular Research Group<br>Barker, Diane; Keele University, Keele Cardiovascular Research Group<br>Heatlie, Grant; Keele University, Keele Cardiovascular Research Group<br>Zaman, Azfar; Freeman Hospital, Department of Cardiology<br>Mamas, Mamas; University of Manchester, Health eResearch Centre, Farr Institute; Keele University, Keele Cardiovascular Research Group

**Primary Subject Heading**:	Health informatics
Secondary Subject Heading:	Cardiovascular medicine
Keywords:	Myocardial infarction < CARDIOLOGY, Coronary heart disease < CARDIOLOGY, Human resource management < HEALTH SERVICES ADMINISTRATION & MANAGEMENT

SCHOLARONE™
Manuscripts

Effect of Weekend Admission on Process of Care and Clinical Outcomes for the Management of Acute Coronary Syndromes: a retrospective analysis of three UK centres

Glen P. Martin, MSc *[1]; Tim Kinnaird, MBBCh, MD *[2, 3]; Matthew Sperrin, PhD [1]; Richard Anderson, BSc, MBBS, MD [3]; Amr Gamal MBBCh, MSc , MD [4]; Avais Jabbar, MBBS [4]; Chun Shing Kwok, MBBS, BSc, MSc [2, 5]; Diane Barker, MB ChB, MD [5]; Grant Heatlie, BSc, PhD, MBBS [5]; Azfar G. Zaman, BSc, MBChB, MD [4]; Mamas A. Mamas, MA, DPhil, FRCP [1, 2,5]

* Joint first authors: these authors contributed equally to manuscript

1: Farr Institute, Faculty of Biology, Medicine and Health, University of Manchester, Manchester Academic Health Science Centre, Manchester, United Kingdom

2: Keele Cardiovascular Research Group, Centre for Prognosis Research, Institutes of Applied Clinical Science and Primary Care and Health sciences, Keele University, Stoke-on-Trent, United Kingdom

3: Department of Cardiology, University Hospital of Wales, Cardiff, United Kingdom

4: Department of Cardiology, Freeman Hospital and Institute of Cellular Medicine, Newcastle University, Newcastle-upon-Tyne, United Kingdom

5: Academic Department of Cardiology, Royal Stoke Hospital, University Hospital North Midlands, Stoke-on-Trent, United Kingdom

Manuscript Word Count: 3222

Running title: Effect of weekend admission on outcomes after ACS

Corresponding author:
Mamas A. Mamas

Professor of Cardiology / Honorary Consultant Cardiologist

Keele Cardiovascular Research Group, Guy Hilton Research Centre, Thornburrow Drive,

Keele University, Stoke-on-Trent, ST4 7QB, UK

Email: mamasmamas1@yahoo.co.uk

For peer review only

Abstract

Objectives

The effect of weekend versus weekday admission following acute coronary syndrome (ACS) on process of care and mortality remains controversial. This study aimed to investigate the "weekend-effect" on outcomes using a multicentre dataset of ST-elevation myocardial infarction (STEMI) and non-ST-elevation myocardial infarction/ unstable angina (NSTEMI/ UA) patients.

Design

This retrospective observational study used propensity score stratification to adjust estimates of weekend effect for observed confounding. Logistic regression was used to estimate odds ratios (ORs) for binary outcomes and time-to-event endpoints were modelled using Cox proportional hazards to estimate hazard ratios (HRs).

Setting

Three tertiary cardiac centres in England and Wales that contribute to the Myocardial Ischaemia National Audit Project (MINAP).

Participants

Between January 2010 and March 2016, 17,705 admissions met the study inclusion criteria, 4,327 of which were at a weekend.

Primary and Secondary Outcomes

Associations were studied between weekend admissions and the following primary outcome measures: in-hospital mortality, 30-day mortality and long-term survival; secondary outcomes included several processes of care indicators, such as time to coronary angiography.

For peer review only - http://bmjopen.bmj.com/site/about/guidelines.xhtml

Results

After PS stratification adjustment, mortality outcomes were similar between weekend and weekday admission across STEMI and NSTEMI/ UA patients. Weekend admissions were less likely to be discharged within one day (HR 0.72, 95% CI: 0.66, 0.78), but after 4 days the length of stay was similar (HR 0.97, 95% CI: 0.90, 1.04). Fewer NSTEMI/ UA patients received angiography between 0 and 24 hours at a weekend (HR 0.71, 95% CI: 0.65, 0.77). Weekend STEMI patients were less likely to undergo an angiogram within 1 hour, but there was no significant difference after this time point.

Conclusion

ACS patients had similar mortality and processes of care when admitted on a weekend compared with a weekday. There was evidence of a delay to angiography for NSTEMI/ UA patients admitted at the weekend.

Keywords

Myocardial infarction – Process of care – Acute Coronary Syndrome - Resource management – Weekend effect

For peer review only

For peer review only - http://bmjopen.bmj.com/site/about/guidelines.xhtml

Article Summary

Strengths and limitations of this study

- An analysis of a weekend effect after acute coronary syndromes in contemporary practice.
- This study analysed data derived from a robust national database (MINAP), of three large tertiary interventional centres.
- Included both ST elevation myocardial infarction cases and non-ST elevation myocardial infarction/ unstable angina cases.
- Any analysis of a weekend effect will be confounded by patient demographics, healthcare systems and the hospitals involved, thus limiting the generalisability of the results.
- Due to the observational nature of the study, unmeasured confounders potentially influence the conclusions.

For peer review only - http://bmjopen.bmj.com/site/about/guidelines.xhtml

Introduction

A timely revascularization following presentation with an acute coronary syndrome (ACS) can mitigate loss of cardiac function and reduce morbidity and mortality [1,2]. Invasive procedures such as primary percutaneous coronary intervention (PCI) are central to the care of patients presenting with ST-elevation myocardial infarction (STEMI) [3] and guidelines also recommend an early invasive strategy for non-ST-elevation myocardial infarction (NSTEMI) cases [4,5].

Nevertheless, heterogeneity in hospital resource allocation, service provision and staffing levels across weekday and weekends can affect the delivery of optimal care, leading to studies of the so-called "weekend-effect" [6–8]. Higher rates of mortality have been indicated in patients admitted to hospital for an acute myocardial infarction (AMI) at a weekend, an evening or holiday period [7,9,10]. However, previous results in AMI patients are inconsistent [11–13]. An analysis of a multicentre registry highlighted that, despite weekend admission being associated with a delay in invasive therapy, this did not impact rates of adverse events [13]. Similarly, the "Get With the Guidelines-Coronary Artery Disease" database showed that despite longer door-to-balloon time, mortality rates were similar between 'out-of-hour' and 'in-hour' hospital admission [11].

Many previous studies have focused on outcome differences across weekend/ weekday admissions in STEMI patients, with limited data for patients with NSTEMI or unstable angina (NSTEMI/ UA). Service provision and delivery, healthcare systems, deprivation and population demographics vary both temporally and by geography, thereby confounding the analysis of a weekend-effect; even the definition of weekend or "out-of-hours" admission varies between populations. Such factors might explain, in part, the inconsistencies across previous studies. Therefore, the aim of this study was to examine the

For peer review only - http://bmjopen.bmj.com/site/about/guidelines.xhtml

effect of weekend vs. weekday admission on outcomes following presentation with STEMI or NSTEMI/ UA, in a contemporary, multicentre dataset of the UK.

Methods

MINAP Dataset

The Myocardial Ischaemia National Audit Project (MINAP) collects prospective data on the management of heart attacks in the UK [14]. Each centre is responsible for data entry into MINAP, based on agreed definitions and options for each variable. This study had access to data from three contributing centres, namely the Freeman Hospital (Newcastle-upon-Tyne), the Royal Stoke Hospital (Stoke-on-Trent) and the University Hospital of Wales (Cardiff). The dataset includes variables detailing patient characteristics, emergency response/ admission dates, processes of care, and outcomes within hospital discharge. Long-term mortality tracking was available from the Office for National Statistics for English patients and the Welsh Demographic Service for Welsh patients.

Study Design

This retrospective analysis included all patients who had a discharge diagnosis of an ACS and were sub-classified into ST elevation myocardial infarction, non-ST elevation myocardial infarction, or unstable angina. After such inclusion criteria, the STEMI sub-group was defined as those patients with an admission and/or discharge diagnosis of ST elevation Myocardial Infarction; all other patients meeting the discharge diagnosis inclusion criteria were included in the NSTEMI/UA sub-group.

Using hospital admission dates, patients were classified into weekend or weekday admission (reference group). In line with previous studies, weekends were defined as any admission between 12:00am Saturday and 11:59pm Sunday, with weekday defined as any admissions outside this time window [7–9]. The primary outcome measures were in-hospital

For peer review only - http://bmjopen.bmj.com/site/about/guidelines.xhtml

mortality, 30-day mortality and long-term survival. Secondary outcomes included the following process of care indicators: admission to a cardiology ward, length of stay (defined as whole number of days between admission date and discharge date), performance of coronary angiography/ echocardiography, time to coronary angiography (defined as number of hours between admission time and time of angiogram), and discharged on beta-blocker, ACE-I or statins. Note that any patient admitted and discharged on the same day had length of stay defined as zero. All the outcomes were analysed as a whole cohort (with adjustment for STEMI indication) and separately in the STEMI and NSTEMI/ UA sub-groups.

Statistical Analysis

For descriptive analysis, continuous variables were presented as means with standard deviations and compared using the t-test. Categorical variables were presented as frequencies of occurrence and compared using the chi-squared test. Patients with missing endpoint variables were excluded from the analysis of that endpoint. Other patient characteristic variables that were missing were assigned to the gender-matched median value for continuous variables or the reference category for categorical variables.

To control for potential differences in baseline covariates between weekend and weekday admission, propensity scores (PS) for being admitted at a weekend were calculated for each patient [15,16]. Although day of hospital admission is not a modifiable intervention, one could regard this in the context of differences in resource allocation between the weekend and weekday. Variables included in the logistic regression model to calculate the PS included all those given in **Table 1** and an admitting centre indicator. Patients were stratified into five strata using their PS, with the cut-off values determined by the quintiles of the PS distribution for all patients. Such PS stratification has been shown to remove 90% of the bias due to the covariates within the PS model [15,16].

For peer review only - http://bmjopen.bmj.com/site/about/guidelines.xhtml

Within each PS strata, binary outcomes (mortality, admission to cardiology ward, performance of coronary angiography/ echocardiography and prescription of medications) were compared directly between admission day groups (using logistic regression), with the odds ratios (ORs) pooled by the Mantel-Haenszel method [17]. For time-to-event outcomes (length of stay, time-to-angiography and long-term survival), the unadjusted survival curves were obtained using the Kaplan-Meier estimate and adjusted hazards ratios (HRs) were estimated using a Cox proportional hazards model. The Cox proportional hazards model included weekend indication as the covariate and was stratified by PS strata. The proportional hazards assumption of the weekend effect variable was checked by examining the Schoenfeld residuals against time; weekend-by-time interactions were included where necessary. We did not model length of stay beyond 50 days, or time-to-angiography beyond 7 days for NSTEMI/ UA and beyond 5 hours for STEMI. All estimates are reported with corresponding 95% confidence intervals (CIs).

R version 3.3.1 [18] was used for all statistical analyses. Graphical plots where made using the "ggplot2" package [19], and the "survival" package was used for time-to-event analyses [20,21].

Results

There were 18,166 admissions across the three centres between 1st January 2010 and 30th March 2016. This comprised 3,140 admissions in University Hospital of Wales, 7,023 admissions in Royal Stoke Hospital and 8,003 admissions in Freeman Hospital. Together, 461 admissions did not meet the discharge diagnosis inclusion criteria. Thus, the analysis sample size was 17,705 with n=9,322 admissions for a STEMI and n=7,887 admissions for an NSTEMI/ UA; the type of ACS was unknown in the remaining 496 records, which were removed from the STEMI and NSTEMI/ UA sub-group analyses.

For peer review only - http://bmjopen.bmj.com/site/about/guidelines.xhtml

Table 1 gives the patient baseline characteristics by weekend or weekday admission. Patients admitted on a weekday were older (p=0.001), were more likely female (p=0.028), and had higher rates of previous AMI (p=0.011), previous angina (p=0.002), cerebrovascular disease (p=0.010), asthma/chronic obstructive pulmonary disease (p=0.044) and previous PCI (p=0.014). Conversely, weekend patients were more likely to have elevated enzymes (p<0.001) or present with ST elevation (p<0.001), although Killip class did not significantly differ between admission groups. Importantly, rates of admission to a cardiologist were not different across weekend or weekday cohorts, with both being over 96% (p=0.216).

Mortality

Figure 1 shows hospital mortality rates by day of the week. There was no apparent trend in either overall hospital mortality or cardiac/ non-cardiac related deaths. In the whole cohort, the observed hospital mortality rate was 3.96% and 3.41% for weekday and weekend admission groups, respectively (OR 0.86, 95% CI: 0.71, 1.03) (**Table 2**). After PS stratification, the pooled OR was 0.82 (95% CI: 0.58, 1.09). Similar findings were found across STEMI and NSTEMI/ UA sub-groups (**Table 3**).

Overall one-, three- and five-year survival rates were 89.7%, 82.3% and 74.9%, respectively. As a whole cohort, there was no significant difference in the Kaplan-Meier survival curves between admission groups (log-rank test, p=0.569) (**Figure 2**). The PS adjusted HR from a Cox proportional hazards model for the whole cohort was 0.99 (95% CI: 0.90, 1.09) for weekend over weekday admission. In STEMI cases, the Kaplan-Meier curves were significantly different (log-rank test, p=0.032), but this was not observed for NSTEMI/ UA cases (log-rank test, p=0.103). After PS adjustment, long-term survival was not significantly different between weekday or weekend admissions, with a HR for STEMI cases of 0.94 (95% CI: 0.82, 1.07) and for NSTEMI/ UA cases of 1.02 (95% CI: 0.89, 1.18).

For peer review only - http://bmjopen.bmj.com/site/about/guidelines.xhtml

Admission Process

In the weekend group, 4045 of 4318 (93.7%) patients were admitted to either a cardiac ward or a coronary care unit, compared with 12548 of 13345 (94.0%) in the weekday group (**Table 2**). Rates of admission to a cardiac ward or coronary care unit were not significantly different between the weekend and weekday, with an adjusted OR of 0.97 (95% CI: 0.76, 1.23). In STEMI admissions the adjusted OR was 1.08 (95% CI: 0.67, 1.52) and in NSTEMI/ UA admissions was 0.93 (95% CI: 0.71, 1.20) (**Table 3**).

There was a significant difference in the Kaplan-Meier curves for length of stay between weekday and weekend admission for STEMI (log-rank p=0.001) and for NSTEMI/ UA patients (log-rank p<0.001) (**Supplementary Figure 1**). A time-dependent Cox proportional hazards model indicated that patients admitted during the weekend were significantly less likely to be discharged within one day (HR 0.72, 95% CI: 0.66, 0.78), but more likely to be discharged between 1 and 4 days (HR 1.13, 95% CI: 1.08, 1.18). For instance, 21.7% of weekday admission patients were discharged within one day, compared with 14.7% at the weekend. Between 4 and 50 days, the length of stay between weekday and weekend admission was comparable (**Table 4**). Similar findings were found when examining length of stay across STEMI and NSTEMI/ UA sub-groups (**Supplementary Table 1**).

Coronary Angiography

There was no significant difference in the proportion of patients receiving a coronary angiography between weekday and weekend admission (OR 0.91, 95% CI: 0.71, 1.13) (**Table 2**). In STEMI cases, 96.5% and 96.6% of patients underwent an angiography on a weekday and weekend, respectively (adjusted OR 0.95, 95% CI: 0.62, 1.36). Similarly, NSTEMI/ UA patients did not have significantly different odds of undergoing a coronary angiography by weekend admission, after PS stratification adjustment (**Table 3**).

For peer review only - http://bmjopen.bmj.com/site/about/guidelines.xhtml

Within the group of patients who underwent a coronary angiography, we assessed the time-to-angiography where time of the angiogram was also available (n=7,284 STEMI and n=5,705 NSTEMI/ UA). There was a significant difference in the cumulative event rates for time-to-angiography for NSTEMI/ UA (log-rank p<0.001), and for STEMI cases (log-rank p=0.010) (**Figure 3**). In NSTEMI/ UA cases, the number of patients receiving angiography within 24 hours differed significantly by weekend admission groups, with similar findings at 72 hours (**Supplementary Table 2**). For instance, 88.9% of weekend NSTEMI/ UA admissions received an angiography within 72 hours, compared with 91.1% of weekday NSTEMI/ UA admissions (p=0.028). After PS adjustment, the time dependent Cox proportional hazards model indicated that weekend NSTEMI/ UA patients were less likely to undergo a coronary angiography within 2 hours (HR 0.54, 95% CI: 0.42, 0.69) and between 2-24 hours (HR 0.71, 95% CI: 0.65, 0.77), with the observed mean time-to-angiography being approximately 3 hours longer within the weekend cohort by 24 hours (**Table 5**). Baseline NSTEMI/ UA patient characteristics between those undergoing angiography within 24 hours and those after 24 hours are given in **Supplementary Table 3**. NSTEMI/ UA patients who underwent angiography within 24 hours were more likely to have an ACS admission diagnosis (p<0.001), previous angina (p=0.024), hypercholesterolemia (p<0.001) or a family history of coronary heart disease (p<0.001), and were significantly more likely to be admitted to a cardiologist (p<0.001) compared with those over 24 hours.

In STEMI patients, after PS adjustment the Cox proportional hazards model indicated that there was a slight delay for weekend admission within 1 hour (HR 0.89, 95% CI: 0.84, 0.94) (**Table 5**). Specifically, between 0 and 1 hour, the observed mean time-to-angiography was 0.55 hours and 0.57 hours for weekday and weekend admission, respectively. After 1 hour, there was no significant difference in time-to-angiography between weekend or weekday STEMI admissions after PS adjustment (**Table 5**). Similarly, we examined door-to-

For peer review only - http://bmjopen.bmj.com/site/about/guidelines.xhtml

For peer review only

balloon time in those STEMI patients who underwent primary PCI as the initial reperfusion treatment and where time of reperfusion was also available. Here, door-to-balloon time was longer than 90-minutes (as recommended by the American College of Cardiology) in 6.95% of weekday admissions, compared with 9.12% of weekend admissions (p=0.004). Likewise, the European Society of Cardiology guideline of door-to-balloon time within 120 minutes was exceeded significantly more in weekend admissions (4.13%) compared with weekday admissions (2.96%) (p=0.02).

Echocardiography

At the weekend, 54.9% of patients underwent an echocardiogram, compared with 51.3% of weekday admissions, with both the unadjusted and PS adjusted ORs being significantly different from one (**Table 2**). Specifically, the pooled OR from the PS stratification was 1.16 (95% CI: 1.01, 1.31), indicating that patients admitted over the weekend had 16% greater odds of undergoing an echocardiography compared with those admitted during the week.

Prescribed Medication on discharge

The unadjusted analysis of both prescription of beta-blocker on discharge and prescription of ACE-I on discharge indicated significantly increased odds for those admitted at the weekend (**Table 2**). However, these findings were not significant after adjusting for PS. The crude proportions of statins on discharge were similar across the two groups, at 95.5% for both weekday and weekend admission (adjusted OR 0.96, 95% CI: 0.68, 1.24).

Discussion

The findings of the current study can be summarised as follows: 1) in current UK practice, patients admitted to 3 tertiary cardiac centres during weekends do not present with a higher baseline risk than weekday patients; 2) there was no difference in short- or long-term

For peer review only - http://bmjopen.bmj.com/site/about/guidelines.xhtml

survival between weekends and weekday groups; 3) for NSTEMI patients there was a delay in angiography for patients admitted at a weekend; 4) other metrics of care quality such as admission under a cardiologist, performance of echocardiography and discharge medications were not adversely affected by a weekend admission.

The lack of a weekend effect in the treatment of an ST elevation myocardial infarction in a contemporary UK cohort is not surprising. The most recently published MINAP data for 2013/14 demonstrates that for the whole of the UK, 98.5% of patients with ST elevation received primary PCI, with few patients receiving thrombolysis [22]. Amongst its many benefits, with respect to day of the week, a primary PCI pathway is a consultant-led and consultant-delivered service, which is undertaken regardless of the time of day or day of the week. Therefore, STEMI treatment in the UK is treated on a 24/7 basis and unless the patients admitted at a weekend are of a higher-risk profile, then it would not be expected that a weekend admission would be disadvantageous to their outcomes.

However, pathways for the treatment of NSTEMI/ UA are markedly different in the UK to those for STEMI. The findings of the current study identify differences in patient treatment with respect to their timings, but not regarding patient survival. The lack of a mortality difference for a weekend NSTEMI admission may, in part, be due to the high percentage of cases admitted under the care of a cardiologist regardless of day of admission (>96% for both cohorts). Consequently, the patients may well have received optimal care in a timely fashion. Crucially, review by a cardiologist early in a patient's admission can identify patients who are unstable or deemed to be high-risk NSTEMI patients and these can be prioritised and fast-tracked to the catheter-lab along a pathway similar to primary PCI. Early identification of high-risk NSTEMI patients by the admitting cardiologist and performing coronary angiography within 2 hours, regardless of the day of the week, can ensure that this risk is ameliorated by invasive management [23]. Indeed, several UK centres have developed

For peer review only - http://bmjopen.bmj.com/site/about/guidelines.xhtml

direct transfer protocols to reduce time-to-angiography for patients with NSTEMI (the so-called HAC-X group) [24]. The optimal timing of angiography is less clear for intermediate-risk NSTEMI cases, with a lack of consistency in published trials [25–27]. In a recent meta-analysis of 10 time-to-angiography trials, there was no difference in mortality between patients receiving angiography early (within 24-hours) or late (24-86 hours) [28]. Therefore, although the European Society of Cardiology guidelines suggest that the high-risk cohort receive angiography within 24 hours, they recommend that the intermediate-risk cohort receive angiography within 72 hours [5]. In the current study, the proportion of NSTEMI/ UA patients receiving angiography within 24 and 72 hours was significantly lower for weekend admissions compared with weekday admissions.

Although the current study does not demonstrate a weekend-effect on patient outcomes, it does demonstrate an effect on length of stay, which is increasingly used as an endpoint to monitor quality of service. The HAC-X group demonstrated that a direct transfer protocol for angiography in NSTEMI patients reduced the length of stay by six days per admission [24]. Therefore, although 7 day working (and with it angiography) may not improve patient survival after an ACS, it is likely to significantly reduce their length of stay. A prolonged hospital admission has been closely associated with iatrogenic complications such as infection and venous thrombosis [29,30]. Therefore, as well as having significant implications on bed utilisation, efficiency and costs, performing angiography on NSTEMI patients at the weekend is likely to improve patients' experience and reduce their potential hospital acquired morbidity. Speculatively, the longer length of stay for weekend admissions might explain the finding of higher rates of echocardiograms performed at weekends (or *vice versa*).

Several factors might explain the differences in the results of previous studies with the current one. Firstly, the data analysed in the current study is derived from the period 2010-

For peer review only - http://bmjopen.bmj.com/site/about/guidelines.xhtml

2016 and hence reflects contemporary ACS management. Current medical practice has evolved greatly over the last decade. Therefore, the findings of older studies will not necessarily be expected to be relevant or similar to a more contemporary cohort [6–8]. Secondly, the three centres involved in the current study are large university teaching hospitals with a daily review of patients by a senior cardiologist, and 24/7 access to echocardiography and cardiac catheterisation. Therefore, data that includes significant numbers of admissions to smaller hospitals without 24/7 cardiology services might lead to differing outcomes for patients admitted during the weekends compared to a weekday. Finally, differing service provision, patient populations, demographics and healthcare systems might also lead to conflicting results of previous studies with the current study.

The strengths of the current study are that it is an analysis of contemporary practice, the data is derived from a robust national database, and the three centres involved are large tertiary interventional centres with high numbers of patients. However, as with any retrospective analysis there are limitations and the robustness of the conclusions are directly related to the quality of data entered. Unmeasured confounders inherent in observational studies of this nature potentially influence any conclusions. Additionally, this study included only those patients with a discharge diagnosis of STEMI or NSTEMI/ UA; consequently, outcomes might differ if the cohort was defined based solely on admission diagnosis. Finally, our findings might not generalise to other hospitals or healthcare systems due to heterogeneity in patient demographics, the healthcare system, and the hospitals involved.

Conclusions

In regional, university cardiac centres, with specialist cardiology services in the UK, ACS patients had similar mortality and process of care when admitted at a weekend compared with a weekday. Delays to time-to-angiography occurred in NSTEMI/ UA patients admitted at the weekend compared to weekdays, but this did not influence survival.

For peer review only - http://bmjopen.bmj.com/site/about/guidelines.xhtml

For peer review only

Funding Statement

This work was funded by the Medical Research Council through the Health e-Research Centre, University of Manchester [MR/K006665/1] and a grant through the North Staffordshire Heart Committee.

Competing Interests Statement

The authors have no conflict of interest.

Author Contributions

TM, AGZ, MAM contributed to the acquisition of the data for the analysis. GPM, TK, MS and MAM made substantial contributions to the concept of the work in addition to performing the analysis. GPM and TK drafted the initial version of the manuscript. GPM, TK, MS, RA, AG, AJ, CSK, DB, GH, AGZ and MAM interpreted the results, revised the paper critically for important intellectual content and approved the final version of the paper. All authors agreed to be accountable for all aspects of the work.

Data sharing statement

The statistical code used to analyse the data is available upon reasonable request by emailing MAM (corresponding author).

Ethics

The National Institute for Cardiovascular Outcomes Research (NICOR) which includes the MINAP database (Ref: NIGB: ECC 1-06 (d)/2011) has support under section 251 of the National Health Service Act 2006 to use patient information for medical research without informed consent. Further ethical approval was not required under current National Health

For peer review only - http://bmjopen.bmj.com/site/about/guidelines.xhtml

Service research governance arrangements, as all data analysed in the study was pseudonymised and contained no patient identifiable information.

For peer review only

For peer review only - http://bmjopen.bmj.com/site/about/guidelines.xhtml

Figure Legends

Figure 1: In-hospital mortality rates by the day of arrival decomposed into cardiac and non-cardiac related mortality.

Figure 2: Long-term survival following weekend versus weekday admission as a whole cohort and across STEMI and NSTEMI/ UA sub-groups.

Figure 3: Cumulative event rate for time to coronary angiography following weekend versus weekday admission across STEMI and NSTEMI/ UA sub-groups.

For peer review only

For peer review only - http://bmjopen.bmj.com/site/about/guidelines.xhtml

Tables

Table 1: Baseline Summary

Variable	Whole Cohort [n=17705]	Weekday Admission [n=13378]	Weekend Admission [n=4327]	p-value	Missing (% of whole cohort)
Age at Admission, mean (SD)	66.0 (13.0)	66.2 (13.0)	65.5 (13.1)	0.001	7 (0.04)
Male, n (%)	12530 (70.8)	9410 (70.3)	3120 (72.1)	0.028	0 (0.00)
Caucasian, n (%)	12283 (69.4)	9360 (70.0)	2923 (67.6)	0.003	4988 (28.2)
Admission Diagnosis					0 (0.00)
Acute coronary syndrome, n (%)	15427 (87.1)	11689 (87.4)	3738 (86.4)	0.097	
Chest pain unknown cause, n (%)	1759 (9.94)	1316 (9.84)	443 (10.2)	0.461	
Other, n (%)	519 (2.93)	373 (2.79)	146 (3.37)	0.053	
Previous AMI, n (%)	3768 (21.3)	2907 (21.7)	861 (19.9)	0.011	109 (0.62)
Previous Angina, n (%)	3968 (22.4)	3073 (23.0)	895 (20.7)	0.002	282 (1.59)

For peer review only - http://bmjopen.bmj.com/site/about/guidelines.xhtml

Hypertension, n (%)	9392 (53.0)	7153 (53.5)	2239 (51.7)	0.050	255 (1.44)
Hypercholesterolemia, n (%)	7166 (40.5)	5458 (40.8)	1708 (39.5)	0.127	574 (3.24)
PVD, n (%)	809 (4.57)	626 (4.68)	183 (4.23)	0.234	85 (0.48)
Cerebrovascular Disease, n (%)	1270 (7.17)	998 (7.46)	272 (6.29)	0.010	83 (0.47)
Asthma or COPD, n (%)	2717 (15.3)	2095 (15.7)	622 (14.4)	0.044	114 (0.64)
Chronic Renal Failure, n (%)	691 (3.90)	527 (3.94)	164 (3.79)	0.693	143 (0.81)
Heart Failure, n (%)	508 (2.87)	379 (2.83)	129 (2.98)	0.649	159 (0.90)
Enzymes Elevated, n (%)	16378 (92.5)	12304 (92.0)	4074 (94.2)	<0.001	634 (3.58)
Previous/ Current Smoker, n (%)	11892 (67.2)	9021 (67.4)	2871 (66.4)	0.195	550 (3.11)
Cholesterol, mean (SD)	4.77 (1.35)	4.76 (1.35)	4.82 (1.38)	0.015	4590 (25.9)
Diabetes					126 (0.71)
Dietary control, n (%)	609 (3.44)	464 (3.47)	145 (3.35)	0.749	
Oral medicine, n (%)	1870 (10.6)	1394 (10.4)	476 (11.0)	0.293	
Insulin, n (%)	721 (4.07)	571 (4.27)	150 (3.47)	0.023	
Insulin and medication, n (%)	297 (1.68)	224 (1.67)	73 (1.69)	0.999	
Previous PCI, n (%)	2124 (12.0)	1651 (12.3)	473 (10.9)	0.014	17 (0.10)

For peer review only - http://bmjopen.bmj.com/site/about/guidelines.xhtml

Previous CABG, n (%)	959 (5.42)	732 (5.47)	227 (5.25)	0.595	4 (0.02)
Systolic BP, mean (SD)	135.3 (27.7)	135.1 (27.5)	135.8 (28.3)	0.219	2574 (14.5)
Heart Rate, mean (SD)	76.8 (19.3)	76.5 (19.1)	77.5 (19.8)	0.006	2257 (12.7)
Admitting Consultant					52 (0.29)
Cardiologist, n (%)	17093 (96.5)	12929 (96.6)	4164 (96.2)	0.216	
Other general physician, n (%)	445 (2.51)	325 (2.43)	120 (2.77)	0.230	
Other, n (%)	115 (0.65)	88 (0.66)	27 (0.62)	0.895	
Beta Blocker, n (%)	4670 (26.4)	3555 (26.6)	1115 (25.8)	0.306	617 (3.48)
Statin, n (%)	6997 (39.5)	5360 (40.1)	1637 (37.8)	0.009	630 (3.56)
Glucose, mean (SD)	8.60 (4.60)	8.55 (4.61)	8.76 (4.58)	0.015	3044 (17.2)
Height, mean (SD)	170.1 (9.76)	170.0 (9.81)	170.5 (9.57)	0.011	3514 (19.8)
Weight, mean (SD)	81.9 (18.7)	81.8 (18.8)	82.0 (18.3)	0.624	2571 (14.5)
Family History of CHD, n (%)	8287 (46.8)	6273 (46.9)	2014 (46.5)	0.705	1939 (11.0)
Creatinine, mean (SD)	95.7 (64.6)	95.5 (63.3)	96.3 (68.4)	0.502	266 (1.50)
Haemoglobin, mean (SD)	136.6 (19.1)	136.3 (19.2)	137.5 (18.8)	<0.001	195 (1.10)

For peer review only - http://bmjopen.bmj.com/site/about/guidelines.xhtml

For peer review only

Killip class					6919 (39.1)
No evidence of Heart Failure, n (%)	9103 (51.4)	6918 (51.7)	2185 (50.5)	0.170	
Basal Crepitations, n (%)	811 (4.58)	590 (4.41)	221 (5.11)	0.062	
Pulmonary Oedema, n (%)	600 (3.39)	453 (3.39)	147 (3.40)	0.999	
Cardiogenic Shock, n (%)	266 (1.50)	195 (1.46)	71 (1.64)	0.430	
Cardiac Arrest, n (%)	1493 (8.43)	1106 (8.27)	387 (8.94)	0.174	0 (0.00)
Thienopyridene, n (%)	15632 (88.3)	11774 (88.0)	3858 (89.2)	0.043	109 (0.62)
STEMI, n (%)	9322 (52.7)	6781 (50.7)	2541 (58.7)	<0.001	496 (2.80)

AMI: Acute Myocardial Infarction; BP: Blood Pressure; CABG: Coronary Artery Bypass Graft; CHD: Coronary Heart Disease; COPD: Chronic Obstructive Pulmonary Disease; LVEF: Left Ventricular Ejection Fraction; MI: Myocardial Infarction; PCI: Percutaneous Coronary Intervention; PVD: Peripheral Vascular Disease; STEMI: ST Segment Elevation Myocardial Infarction

For peer review only

Table 2: Event numbers, unadjusted odds ratios (OR) and pooled OR across PS strata. Bold item indicate significant effects at the 5% level.

**Outcome**	**Weekday Admission [n=13378]**	**Weekend Admission [n=4327]**	**Unadjusted OR (95% CI)**	**PS Adjusted OR (95% CI)**
Hospital Mortality	529/13354 (3.96%)	147/4312 (3.41%)	0.86 (0.71, 1.03)	0.82 (0.58, 1.09)
30-day Mortality	553/10874 (5.09%)	182/3545 (5.13%)	1.01 (0.85, 1.20)	0.96 (0.72, 1.28)
Admission to Cardiology Ward	12548/13345 (94.0%)	4045/4318 (93.7)	0.94 (0.82, 1.09)	0.97 (0.76, 1.23)
Coronary Angiography	12401/13265 (93.5%)	3987/4288 (93.0%)	0.92 (0.81, 1.06)	0.91 (0.71, 1.13)
Echocardiography	5772/11262 (51.3%)	2014/3666 (54.9%)	**1.16 (1.08, 1.25)**	**1.16 (1.01, 1.31)**
Discharged on Beta-blocker	10320/12181 (84.7%)	3404/3943 (86.3%)	**1.14 (1.03, 1.26)**	1.06 (0.88, 1.26)
Discharged on ACE-I	10801/12109 (89.2%)	3552/3930 (90.4%)	**1.14 (1.01, 1.29)**	1.09 (0.88, 1.34)
Discharged on Statins	11590/12131 (95.5%)	3764/3942 (95.5%)	0.99 (0.83, 1.18)	0.96 (0.68, 1.24)

For peer review only - http://bmjopen.bmj.com/site/about/guidelines.xhtml

Table 3: Event numbers and pooled odds ratios (OR) across PS strata by STEMI and NSTEMI/ UA sub-groups.

	**STEMI (n=9322)**			**NSTEMI/ UA (n=7887)**		
**Outcome**	**Weekday Admission [n=6781]**	**Weekend Admission [n=2541]**	**PS Adjusted OR (95% CI)**	**Weekday Admission [n=6190]**	**Weekend Admission [n=1697]**	**PS Adjusted OR (95% CI)**
Hospital Mortality	379/6774 (5.59%)	110/2533 (4.34%)	0.83 (0.58, 1.15)	137/6175 (2.22%)	36/1691 (2.13%)	0.82 (0.45, 1.38)
30-day Mortality	379/5633 (6.73%)	132/2123 (6.22%)	0.99 (0.69, 1.35)	157/4913 (3.20%)	45/1352 (3.33%)	0.88 (0.53, 1.41)
Admission to Cardiology Ward	6486/6763 (95.9%)	2442/2533 (96.4%)	1.08 (0.67, 1.52)	5673/6176 (91.9%)	1518/1696 (89.5%)	0.93 (0.71, 1.20)
Coronary Angiography	6512/6749 (96.5%)	2445/2530 (96.6%)	0.95 (0.62, 1.36)	5532/6113 (90.5%)	1465/1669 (87.8%)	0.92 (0.70, 1.15)
Echocardiography	3241/6085 (53.3%)	1267/2210 (57.3%)	1.17 (0.98, 1.38)	2377/4796 (49.6%)	714/1374 (52.0%)	1.16 (0.93, 1.42)
Discharged on Beta-blocker	5226/5976 (87.4%)	2018/2261 (89.3%)	1.08 (0.83, 1.39)	4838/5846 (82.8%)	1328/1600 (83.0%)	1.03 (0.80, 1.32)

For peer review only - http://bmjopen.bmj.com/site/about/guidelines.xhtml

Discharged on ACE-I	5529/5940 (93.1%)	2109/2254 (93.6%)	1.04 (0.72, 1.41)	4998/5815 (86.0%)	1374/1594 (86.2%)	1.13 (0.83, 1.41)
Discharged on Statins	5772/5951 (97.0%)	2187/2260 (96.8%)	0.89 (0.50, 1.32)	5481/5824 (94.1%)	1498/1600 (93.6%)	1.01 (0.68, 1.41)

For peer review only

Table 4: Number of patients discharged within each time-window and PS adjusted hazard ratios (HR) from the time-dependent Cox proportional hazards models for length of stay, as a whole cohort. Bold items indicate significant effects at the 5% level.

**Length of stay**	**Discharged, n (%) Weekday Cohort**	**Discharged, n (%) Weekend Cohort**	**PS adjusted HR (95% CI)**
0 – 1 day	2888 (21.7%)	636 (14.7%)	**0.72 (0.66, 0.78)**
1 - 4 days	6731 (64.5%)	2595 (70.5%)	**1.13 (1.08, 1.18)**
4 - 50 days	3639 (98.2%)	1063 (98.1%)	0.97 (0.90, 1.04)

For peer review only

Table 5: PS adjusted hazard ratios (HR) from time-dependent Cox proportional hazards models for time to coronary angiography in STEMI and NSTEMI/ UA patients. Bold items indicate significant effects at the 5% level.

	**STEMI**		
**Time to coronary angiography***	**Mean time-to-angiography Weekday, (hours)**	**Mean time-to-angiography Weekend, (hours)**	**PS adjusted HR (95% CI)**
0 - 1 hour	0.55	0.57	**0.89 (0.84, 0.94)**
1 - 2 hours	1.52	1.53	0.99 (0.87, 1.11)
2 – 5 hours	3.27	3.11	1.25 (1.00, 1.56)
	**NSTEMI/ UA**		
0 - 2 hours	1.89	1.94	**0.54 (0.42, 0.69)**
2 - 24 hours	15.7	18.3	**0.71 (0.65, 0.77)**
24 hours - 4 days	56.2	53.9	**1.22 (1.09, 1.36)**
4 - 7 days	139.9	137.0	0.94 (0.68, 1.29)

**: The time-windows for the time-dependent Cox proportional hazard model were made to satisfy the proportionality assumption throughout time. In the case of NSTEMI/ UA, we selected 24 hours as one of the time cut-offs to reflect the ESC guidelines for high-risk patients.*

For peer review only - http://bmjopen.bmj.com/site/about/guidelines.xhtml

References

1 Berger PB, Ellis SG, Holmes DRJ, *et al.* Relationship between delay in performing direct coronary angioplasty and early clinical outcome in patients with acute myocardial infarction: results from the global use of strategies to open occluded arteries in Acute Coronary Syndromes (GUSTO-IIb) trial. *Circulation* 1999;**100**:14–20.

2 McNamara RL, Wang Y, Herrin J, *et al.* Effect of Door-to-Balloon Time on Mortality in Patients With ST-Segment Elevation Myocardial Infarction. *J Am Coll Cardiol* 2006;**47**:2180–6. doi:10.1016/j.jacc.2005.12.072

3 O'Gara PT, Kushner FG, Ascheim DD, *et al.* 2013 ACCF/AHA Guideline for the Management of ST-Elevation Myocardial Infarction. *J Am Coll Cardiol* 2013;**61**:e78–140. doi:10.1016/j.jacc.2012.11.019

4 Amsterdam EA, Wenger NK, Brindis RG, *et al.* 2014 AHA/acc guideline for the management of patients with Non-ST-Elevation acute coronary syndromes: A report of the American College of Cardiology/American Heart Association Task Force on Practice Guidelines. *J Am Coll Cardiol* 2014;**64**:e139–228. doi:10.1016/j.jacc.2014.09.017

5 Roffi M, Patrono C, Collet J-P, *et al.* 2015 ESC Guidelines for the management of acute coronary syndromes in patients presenting without persistent ST-segment elevation. *Eur Heart J* 2016;**37**:267–315. doi:10.1093/eurheartj/ehv320

6 Bell CM, Redelmeier DA. Mortality among Patients Admitted to Hospitals on Weekends as Compared with Weekdays. *N Engl J Med* 2001;**345**:663–8. doi:10.1056/NEJMsa003376

7 Kostis WJ, Demissie K, Marcella SW, *et al.* Weekend versus Weekday Admission and Mortality from Myocardial Infarction. *N Engl J Med* 2007;**356**:1099–109.

For peer review only

For peer review only - http://bmjopen.bmj.com/site/about/guidelines.xhtml

doi:10.1056/NEJMoa063355

8 Cram P, Hillis SL, Barnett M, *et al.* Effects of weekend admission and hospital teaching status on in-hospital mortality. *Am J Med* 2004;**117**:151–7. doi:10.1016/j.amjmed.2004.02.035

9 Khoshchehreh M, Groves EM, Tehrani D, *et al.* Changes in mortality on weekend versus weekday admissions for Acute Coronary Syndrome in the United States over the past decade. *Int J Cardiol* 2016;**210**:164–72. doi:10.1016/j.ijcard.2016.02.087

10 Sorita A, Ahmed A, Starr SR, *et al.* Off-hour presentation and outcomes in patients with acute myocardial infarction: systematic review and meta-analysis. *BMJ* 2014;**348**:f7393–f7393. doi:10.1136/bmj.f7393

11 Jneid H, Fonarow GC, Cannon CP, *et al.* Impact of Time of Presentation on the Care and Outcomes of Acute Myocardial Infarction. *Circulation* 2008;**117**:2502–9. doi:10.1161/CIRCULATIONAHA.107.752113

12 Berger A, Stauffer J-C, Radovanovic D, *et al.* Comparison of In-Hospital Mortality for Acute Myocardial Infarction in Switzerland With Admission During Routine Duty Hours Versus Admission During Out of Hours (Insight Into the AMIS Plus Registry). *Am J Cardiol* 2008;**101**:422–7. doi:10.1016/j.amjcard.2007.09.092

13 Ryan JW, Peterson ED, Chen AY, *et al.* Optimal timing of intervention in non-ST-segment elevation acute coronary syndromes: Insights from the CRUSADE (Can Rapid risk stratification of Unstable angina patients Suppress ADverse outcomes with Early implementation of the ACC/AHA guidelines) regist. *Circulation* 2005;**112**:3049–57. doi:10.1161/CIRCULATIONAHA.105.582346

14 Mamas MA, Kwok CS, Kontopantelis E, *et al.* Relationship Between Anemia and Mortality Outcomes in a National Acute Coronary Syndrome Cohort: Insights From the UK Myocardial Ischemia National Audit Project Registry. *J Am Heart Assoc*

For peer review only

For peer review only - http://bmjopen.bmj.com/site/about/guidelines.xhtml

2016;**5**:e003348. doi:10.1161/JAHA.116.003348

15 Rosenbaum PR, Rubin DB. The Central Role of the Propensity Score in Observational Studies for Causal Effects. *Biometrika* 1983;**70**:41–55.

16 Rosenbaum PR, Rubin DB. Reducing Bias in Observational Studies Using Subclassification on the Propensity Score. *J Am Stat Assoc* 1984;**79**:516–24.

17 Mantel N, Haenszel W. Statistical aspects of the analysis of data from retrospective studies of disease. *J Natl Cancer Inst* 1959;**22**:719–48.

18 R Core Team R. R: A Language and Environment for Statistical Computing. R Found. Stat. Comput. 2017. doi:10.1007/978-3-540-74686-7

19 Wickham H. *ggplot2: elegant graphics for data analysis*. Springer New York 2009.

20 Therneau T. A Package for Survival Analysis in S. Published Online First: 2015.http://cran.r-project.org/package=survival

21 Therneau T, Grambsch P. *Modeling Survival Data: Extending the Cox Model*. New York: : Springer 2000.

22 Weston C, Gavalova L, Whittaker T, *et al.* Myocardial Ischaemia National Audit Project: How the NHS cares for patients with heart attack. 2014.https://www.ucl.ac.uk/nicor/audits/minap/documents/annual_reports/minap-public-report-2014

23 de Araujo Goncalves P. TIMI, PURSUIT, and GRACE risk scores: sustained prognostic value and interaction with revascularization in NSTE-ACS. *Eur Heart J* 2005;**26**:865–72. doi:10.1093/eurheartj/ehi187

24 Gallagher SM, Lovell MJ, Jones DA, *et al.* Does a 'direct' transfer protocol reduce time to coronary angiography for patients with non-ST-elevation acute coronary syndromes? A prospective observational study. *BMJ Open* 2014;**4**:e005525. doi:10.1136/bmjopen-2014-005525

For peer review only - http://bmjopen.bmj.com/site/about/guidelines.xhtml

25 Neumann F-J, Kastrati A, Pogatsa-Murray G, *et al.* Evaluation of Prolonged Antithrombotic Pretreatment (‘Cooling-Off’ Strategy) Before Intervention in Patients With Unstable Coronary Syndromes. *JAMA* 2003;**290**:1593–9. doi:10.1001/jama.290.12.1593

26 Montalescot G, Cayla G, Collet J-P, *et al.* Immediate vs Delayed Intervention for Acute Coronary Syndromes. *JAMA* 2009;**302**:947. doi:10.1001/jama.2009.1267

27 van ’t Hof AWJ, de Vries ST, Dambrink J-HE, *et al.* A comparison of two invasive strategies in patients with non-ST elevation acute coronary syndromes: results of the Early or Late Intervention in unStable Angina (ELISA) pilot study. 2b/3a upstream therapy and acute coronary syndromes. *Eur Heart J* 2003;**24**:1401–5. doi:10.1016/S0195-668X(03)00259-8

28 Milasinovic D, Milosevic A, Marinkovic J, *et al.* Timing of invasive strategy in NSTE-ACS patients and effect on clinical outcomes: A systematic review and meta-analysis of randomized controlled trials. *Atherosclerosis* 2015;**241**:48–54. doi:10.1016/j.atherosclerosis.2015.04.808

29 Sbrojavacca R, Pietrangelo A, Fenoglio L, *et al.* Reducing the risk of hospital admission: A call to action from the Italian Society of Internal Medicine. *Eur J Intern Med* 2015;**26**:476–7. doi:10.1016/j.ejim.2015.06.003

30 Lilly CM, Cody S, Zhao H, *et al.* Hospital mortality, length of stay, and preventable complications among critically ill patients before and after tele-ICU reengineering of critical care processes. *JAMA* 2011;**305**:2175–83. doi:10.1001/jama.2011.697

For peer review only

For peer review only - http://bmjopen.bmj.com/site/about/guidelines.xhtml

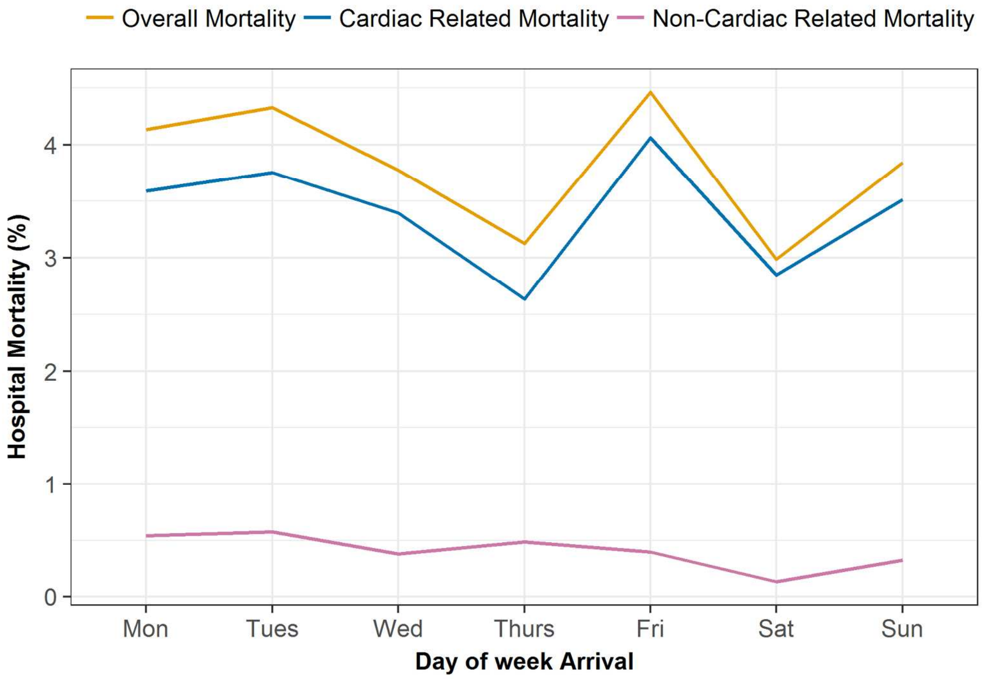

In-hospital mortality rates by the day of arrival decomposed into cardiac and non-cardiac related mortality.

177x127mm (300 x 300 DPI)

view only

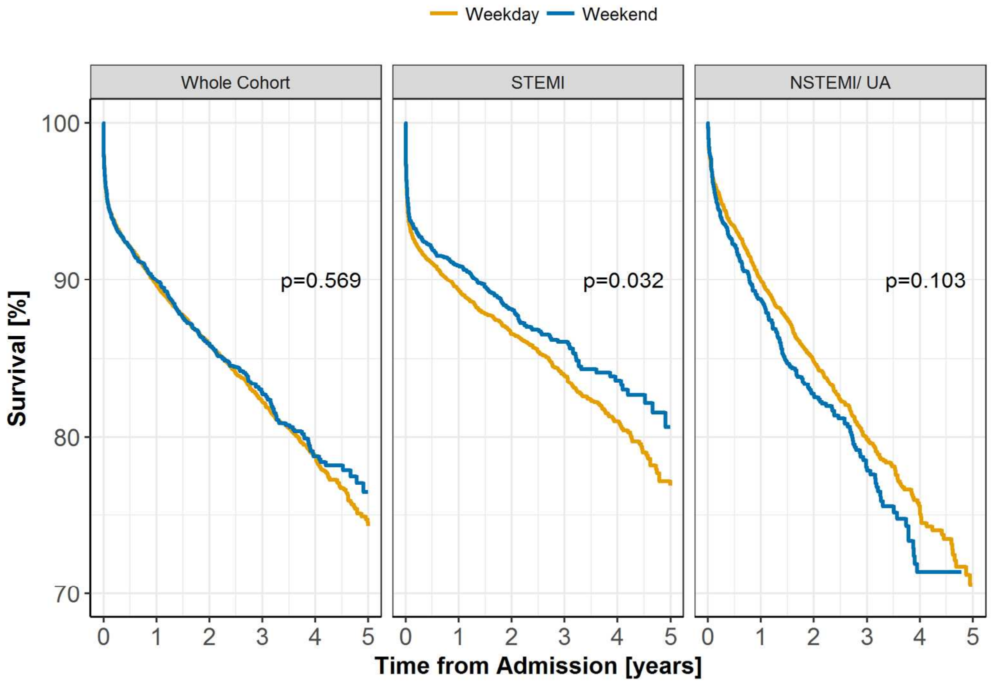

Long-term survival following weekend versus weekday admission as a whole cohort and across STEMI and NSTEMI/ UA subgroups.

177x127mm (300 x 300 DPI)

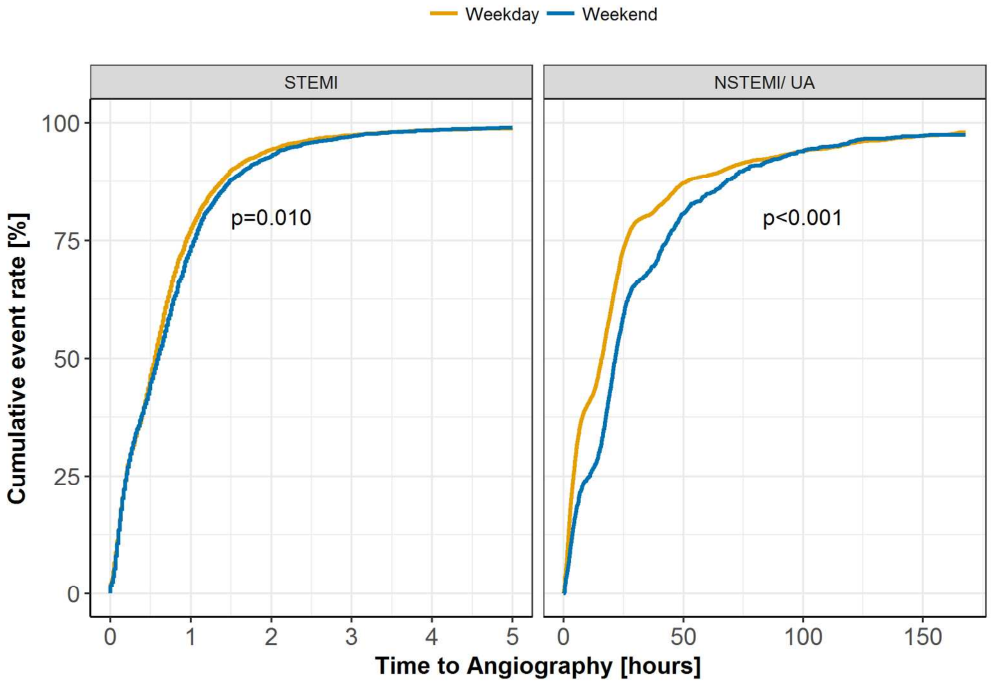

Cumulative event rate for time to coronary angiography following weekend versus weekday admission across STEMI and NSTEMI/ UA subgroups.

177x127mm (300 x 300 DPI)

Supplementary Material

Effect of Weekend Admission on Process of Care and Clinical Outcomes for the Management of Acute Coronary Syndromes

Glen P. Martin*; Tim Kinnaird*; Matthew Sperrin; Richard Anderson; Amr Gamal; Avais Jabbar; Chun Shing Kwok; Diane Barker; Grant Heatlie; Azfar G. Zaman; Mamas A. Mamas

* Joint first authors: these authors contributed equally to manuscript

Supplementary Figures

Supplementary Figure 1: Cumulative event rate for length of stay following weekend versus weekday admission across STEMI and NSTEMI/ UA subgroups.

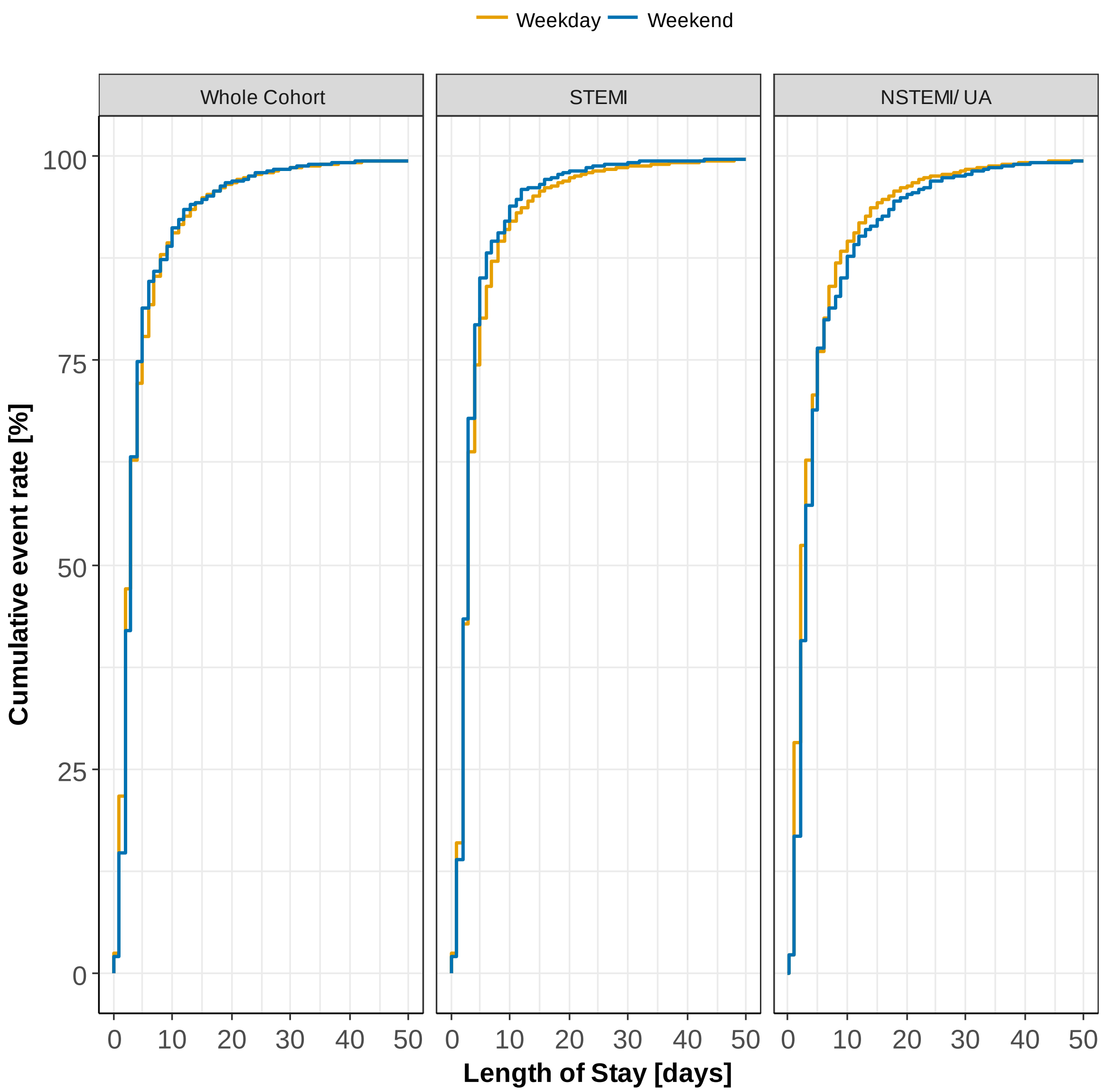

Supplementary Tables

Supplementary Table 1: Number of patients discharged within each time-window and PS adjusted hazard ratios (HR) from the time-dependent Cox proportional hazards models for length of stay, across STEMI and NSTEMI/ UA subgroups. Bold items indicate significant effects at the 5% level.

	**STEMI**		
**Length of stay**	**Discharged, n (%) Weekday Cohort**	**Discharged, n (%) Weekend Cohort**	**HR (95% CI)**
0 – 1 day	1074 (15.9%)	352 (13.9%)	**0.85 (0.75, 0.96)**
1 - 4 days	3953 (69.5%)	1659 (76.0%)	**1.13 (1.06, 1.19)**
4 - 50 days	1702 (98.3%)	513 (98.1%)	1.04 (0.94, 1.15)
	**NSTEMI/ UA**		
0 – 1 day	1733 (28.1%)	283 (16.7%)	**0.65 (0.57, 0.74)**
1 - 4 days	2628 (59.3%)	884 (62.7%)	**1.12 (1.04, 1.21)**
4 - 50 days	1770 (98.1%)	515 (97.9%)	0.93 (0.84, 1.03)

For peer review only

Supplementary Table 2: Proportions of NSTEMI/ UA patients receiving angiography within 24 and 72 hours across weekend admission groups.

Time-to-angiography	Weekday NSTEMI/ UA	Weekend NSTEMI/ UA	p-value
Within 24 hours	3242/ 4529 (71.6%)	662/ 1176 (56.3%)	<0.001
Within 72 hours	4125/ 4529 (91.1%)	1046/ 1176 (88.9%)	0.028

For peer review only

Supplementary Table 3: NSTEMI/UA patient characteristics across those that underwent coronary angiography within 24 hours compared with those after 24 hours.

**Variable**	**Angiography≤24 hours [n=3902]**	**Angiography>24 hours [n=1802]**	**p-value**
Age at Admission, mean (SD)	66.2 (12.4)	67.2 (12.0)	0.004
Male, n (%)	2807 (71.9)	1258 (69.8)	0.109
Caucasian, n (%)	3286 (84.2)	1101 (61.1)	<0.001
Admission Diagnosis			
Acute coronary syndrome, n (%)	3627 (93.0)	1459 (81.0)	<0.001
Chest pain unknown cause, n (%)	253 (6.48)	271 (15.0)	<0.001
Other, n (%)	23 (0.59)	72 (4.00)	<0.001
Previous AMI, n (%)	1169 (30.0)	551 (30.6)	0.659
Previous Angina, n (%)	1555 (39.9)	661 (36.7)	0.024
Hypertension, n (%)	2505 (64.2)	1142 (63.4)	0.567
Hypercholesterolemia, n (%)	2167 (55.5)	877 (48.7)	<0.001
PVD, n (%)	297 (7.61)	164 (9.10)	0.062
Cerebrovascular Disease, n (%)	339 (8.69)	161 (8.93)	0.798
Asthma or COPD, n (%)	647 (16.6)	311 (17.3)	0.550
Chronic Renal Failure, n (%)	126 (3.23)	129 (7.16)	<0.001
Heart Failure, n (%)	147 (3.77)	111 (6.16)	<0.001
Enzymes Elevated, n (%)	3382 (86.7)	1611 (89.4)	0.004
Previous/ Current Smoker, n (%)	2600 (66.6)	1210 (67.1)	0.724
Cholesterol, mean (SD)	4.53 (1.38)	4.48 (1.33)	0.379
Diabetes			
Dietary control, n (%)	157 (4.02)	81 (4.50)	0.449
Oral medicine, n (%)	473 (12.1)	248 (13.8)	0.091
Insulin, n (%)	223 (5.72)	123 (6.83)	0.116
Insulin and medication, n (%)	59 (1.51)	62 (3.44)	<0.001
Previous PCI, n (%)	783 (20.1)	343 (19.0)	0.382
Previous CABG, n (%)	274 (7.02)	192 (10.7)	<0.001
Systolic BP, mean (SD)	136.6 (24.6)	140.3 (25.6)	<0.001
Heart Rate, mean (SD)	71.4 (15.4)	75.8 (18.0)	<0.001
Admitting Consultant			
Cardiologist, n (%)	3893 (99.8)	1708 (94.8)	<0.001
Other general physician, n (%)	4 (0.10)	65 (3.61)	<0.001
Other, n (%)	3 (0.08)	21 (1.17)	<0.001
Beta Blocker, n (%)	1458 (37.4)	713 (39.6)	0.118
Statin, n (%)	2034 (52.1)	988 (54.8)	0.061

For peer review only

Glucose, mean (SD)	7.45 (3.48)	8.16 (4.13)	<0.001
Height, mean (SD)	169.7 (9.90)	169.3 (10.1)	0.251
Weight, mean (SD)	82.7 (17.7)	82.6 (18.5)	0.890
Family History of CHD, n (%)	1942 (49.8)	774 (43.0)	<0.001
Creatinine, mean (SD)	97.6 (54.9)	103.4 (80.4)	0.006
Haemoglobin, mean (SD)	137.4 (18.0)	135.7 (19.6)	0.002
Killip class			
No evidence of Heart Failure, n (%)	3083 (79.0)	1108 (61.5)	<0.001
Basal Crepitations, n (%)	101 (2.59)	172 (9.54)	<0.001
Pulmonary Oedema, n (%)	139 (3.56)	105 (5.83)	<0.001
Cardiogenic Shock, n (%)	14 (0.36)	7 (0.39)	0.999
Cardiac Arrest, n (%)	100 (2.56)	46 (2.55)	0.999
Thienopyridene, n (%)	3384 (86.7)	1662 (92.2)	<0.001

AMI: Acute Myocardial Infarction; BP: Blood Pressure; CABG: Coronary Artery Bypass Graft; CHD: Coronary Heart Disease; COPD: Chronic Obstructive Pulmonary Disease; LVEF: Left Ventricular Ejection Fraction; MI: Myocardial Infarction; PCI: Percutaneous Coronary Intervention; PVD: Peripheral Vascular Disease

For peer review only

STROBE 2007 (v4) checklist of items to be included in reports of observational studies in epidemiology*

Checklist for cohort, case-control, and cross-sectional studies (combined)

Section/Topic	Item #	Recommendation	Reported on page #
**Title and abstract**	1	(*a*) Indicate the study's design with a commonly used term in the title or the abstract	Title page
		(*b*) Provide in the abstract an informative and balanced summary of what was done and what was found	2
**Introduction**			
Background/rationale	2	Explain the scientific background and rationale for the investigation being reported	5
Objectives	3	State specific objectives, including any pre-specified hypotheses	5
**Methods**			
Study design	4	Present key elements of study design early in the paper	6
Setting	5	Describe the setting, locations, and relevant dates, including periods of recruitment, exposure, follow-up, and data collection	6 and 8
Participants	6	(*a*) *Cohort study*—Give the eligibility criteria, and the sources and methods of selection of participants. Describe methods of follow-up<br>*Case-control study*—Give the eligibility criteria, and the sources and methods of case ascertainment and control selection. Give the rationale for the choice of cases and controls<br>*Cross-sectional study*—Give the eligibility criteria, and the sources and methods of selection of participants	6 and 7
		(*b*) *Cohort study*—For matched studies, give matching criteria and number of exposed and unexposed<br>*Case-control study*—For matched studies, give matching criteria and the number of controls per case	N/A
Variables	7	Clearly define all outcomes, exposures, predictors, potential confounders, and effect modifiers. Give diagnostic criteria, if applicable	6 and 7
Data sources/ measurement	8*	For each variable of interest, give sources of data and details of methods of assessment (measurement). Describe comparability of assessment methods if there is more than one group	*6*
Bias	9	Describe any efforts to address potential sources of bias	7
Study size	10	Explain how the study size was arrived at	N/A – registry data
Quantitative variables	11	Explain how quantitative variables were handled in the analyses. If applicable, describe which groupings were chosen and why	7
Statistical methods	12	(*a*) Describe all statistical methods, including those used to control for confounding	7
		(*b*) Describe any methods used to examine subgroups and interactions	7
		(*c*) Explain how missing data were addressed	7
		(*d*) *Cohort study*—If applicable, explain how loss to follow-up was addressed<br>*Case-control study*—If applicable, explain how matching of cases and controls was addressed	N/A

For peer review only

		Cross-sectional study—If applicable, describe analytical methods taking account of sampling strategy	
		(*e*) Describe any sensitivity analyses	N/A
**Results**			
Participants	13*	(a) Report numbers of individuals at each stage of study—eg numbers potentially eligible, examined for eligibility, confirmed eligible, included in the study, completing follow-up, and analysed	8
		(b) Give reasons for non-participation at each stage	8
		(c) Consider use of a flow diagram	N/A
Descriptive data	14*	(a) Give characteristics of study participants (eg demographic, clinical, social) and information on exposures and potential confounders	Table 1
		(b) Indicate number of participants with missing data for each variable of interest	Table 1
		(c) *Cohort study*—Summarise follow-up time (eg, average and total amount)	N/A
Outcome data	15*	*Cohort study*—Report numbers of outcome events or summary measures over time	Table 2 and throughout results section
		Case-control study—Report numbers in each exposure category, or summary measures of exposure	N/A
		Cross-sectional study—Report numbers of outcome events or summary measures	N/a
Main results	16	(*a*) Give unadjusted estimates and, if applicable, confounder-adjusted estimates and their precision (eg, 95% confidence interval). Make clear which confounders were adjusted for and why they were included	Table 2 and page 7
		(*b*) Report category boundaries when continuous variables were categorized	N/A
		(*c*) If relevant, consider translating estimates of relative risk into absolute risk for a meaningful time period	N/A
Other analyses	17	Report other analyses done—eg analyses of subgroups and interactions, and sensitivity analyses	Table 3 and throughout results section
**Discussion**			
Key results	18	Summarise key results with reference to study objectives	12
Limitations	19	Discuss limitations of the study, taking into account sources of potential bias or imprecision. Discuss both direction and magnitude of any potential bias	14 and 15
Interpretation	20	Give a cautious overall interpretation of results considering objectives, limitations, multiplicity of analyses, results from similar studies, and other relevant evidence	15
Generalisability	21	Discuss the generalisability (external validity) of the study results	15
**Other information**			
Funding	22	Give the source of funding and the role of the funders for the present study and, if applicable, for the original study on which the present article is based	Title page

For peer review only

*Give information separately for cases and controls in case-control studies and, if applicable, for exposed and unexposed groups in cohort and cross-sectional studies.
Note: An Explanation and Elaboration article discusses each checklist item and gives methodological background and published examples of transparent reporting. The STROBE checklist is best used in conjunction with this article (freely available on the Web sites of PLoS Medicine at http://www.plosmedicine.org/, Annals of Internal Medicine at http://www.annals.org/, and Epidemiology at http://www.epidem.com/). Information on the STROBE Initiative is available at www.strobe-statement.org.

For peer review only

Effect of Weekend Admission on Process of Care and Clinical Outcomes for the Management of Acute Coronary Syndromes: a retrospective analysis of three UK centres

Journal:	*BMJ Open*
Manuscript ID	bmjopen-2017-016866.R2
Article Type:	Research
Date Submitted by the Author:	07-Aug-2017
Complete List of Authors:	Martin, Glen; University of Manchester, Health eResearch Centre, Farr Institute<br>Kinnaird, Tim; Keele University, Keele Cardiovascular Research Group; University Hospital of Wales, Department of Cardiology<br>Sperrin, Matthew; University of Manchester, Health eResearch Centre, Farr Institute<br>Anderson, Richard; University Hospital of Wales, Department of Cardiology<br>Gamal, Amr; Newcastle University, Department of Cardiology<br>Jabbar, Avais; Newcastle University, Department of Cardiology<br>Kwok, Chun; Keele University, Keele Cardiovascular Research Group<br>Barker, Diane; Keele University, Keele Cardiovascular Research Group<br>Heatlie, Grant; Keele University, Keele Cardiovascular Research Group<br>Zaman, Azfar; Freeman Hospital, Department of Cardiology<br>Mamas, Mamas; University of Manchester, Health eResearch Centre, Farr Institute; Keele University, Keele Cardiovascular Research Group
**Primary Subject Heading**:	Health informatics
Secondary Subject Heading:	Cardiovascular medicine
Keywords:	Myocardial infarction < CARDIOLOGY, Coronary heart disease < CARDIOLOGY, Human resource management < HEALTH SERVICES ADMINISTRATION & MANAGEMENT

Effect of Weekend Admission on Process of Care and Clinical Outcomes for the Management of Acute Coronary Syndromes: a retrospective analysis of three UK centres

Glen P. Martin, MSc *[1]; Tim Kinnaird, MBBCh, MD *[2, 3]; Matthew Sperrin, PhD [1]; Richard Anderson, BSc, MBBS, MD [3]; Amr Gamal MBBCh, MSc , MD [4]; Avais Jabbar, MBBS [4]; Chun Shing Kwok, MBBS, BSc, MSc [2, 5]; Diane Barker, MB ChB, MD [5]; Grant Heatlie, BSc, PhD, MBBS [5]; Azfar G. Zaman, BSc, MBChB, MD [4]; Mamas A. Mamas, MA, DPhil, FRCP [1, 2,5]

* Joint first authors: these authors contributed equally to manuscript

1: Farr Institute, Faculty of Biology, Medicine and Health, University of Manchester, Manchester Academic Health Science Centre, Manchester, United Kingdom

2: Keele Cardiovascular Research Group, Centre for Prognosis Research, Institutes of Applied Clinical Science and Primary Care and Health sciences, Keele University, Stoke-on-Trent, United Kingdom

3: Department of Cardiology, University Hospital of Wales, Cardiff, United Kingdom

4: Department of Cardiology, Freeman Hospital and Institute of Cellular Medicine, Newcastle University, Newcastle-upon-Tyne, United Kingdom

5: Academic Department of Cardiology, Royal Stoke Hospital, University Hospital North Midlands, Stoke-on-Trent, United Kingdom

Manuscript Word Count: 3179

Running title: Effect of weekend admission on outcomes after ACS

Corresponding author:
Mamas A. Mamas

Professor of Cardiology / Honorary Consultant Cardiologist

Keele Cardiovascular Research Group, Guy Hilton Research Centre, Thornburrow Drive,

Keele University, Stoke-on-Trent, ST4 7QB, UK

Email: mamasmamas1@yahoo.co.uk

For peer review only

Abstract

Objectives

The effect of weekend versus weekday admission following acute coronary syndrome (ACS) on process of care and mortality remains controversial. This study aimed to investigate the "weekend-effect" on outcomes using a multicentre dataset of ST-elevation myocardial infarction (STEMI) and non-ST-elevation myocardial infarction/ unstable angina (NSTEMI/ UA) patients.

Design

This retrospective observational study used propensity score stratification to adjust estimates of weekend effect for observed confounding. Logistic regression was used to estimate odds ratios (ORs) for binary outcomes and time-to-event endpoints were modelled using Cox proportional hazards to estimate hazard ratios (HRs).

Setting

Three tertiary cardiac centres in England and Wales that contribute to the Myocardial Ischaemia National Audit Project (MINAP).

Participants

Between January 2010 and March 2016, 17,705 admissions met the study inclusion criteria, 4,327 of which were at a weekend.

Primary and Secondary Outcomes

Associations were studied between weekend admissions and the following primary outcome measures: in-hospital mortality, 30-day mortality and long-term survival; secondary outcomes included several processes of care indicators, such as time to coronary angiography.

For peer review only - http://bmjopen.bmj.com/site/about/guidelines.xhtml

Results

After PS stratification adjustment, mortality outcomes were similar between weekend and weekday admission across STEMI and NSTEMI/ UA patients. Weekend admissions were less likely to be discharged within one day (HR 0.72, 95% CI: 0.66, 0.78), but after 4 days the length of stay was similar (HR 0.97, 95% CI: 0.90, 1.04). Fewer NSTEMI/ UA patients received angiography between 0 and 24 hours at a weekend (HR 0.71, 95% CI: 0.65, 0.77). Weekend STEMI patients were less likely to undergo an angiogram within 1 hour, but there was no significant difference after this time point.

Conclusion

ACS patients had similar mortality and processes of care when admitted on a weekend compared with a weekday. There was evidence of a delay to angiography for NSTEMI/ UA patients admitted at the weekend.

Keywords

Myocardial infarction – Process of care – Acute Coronary Syndrome - Resource management – Weekend effect

For peer review only

Article Summary

Strengths and limitations of this study

- An analysis of a weekend effect after acute coronary syndromes in contemporary practice.
- This study analysed data derived from a robust national database (MINAP), of three large tertiary interventional centres.
- Included both ST elevation myocardial infarction cases and non-ST elevation myocardial infarction/ unstable angina cases.
- Any analysis of a weekend effect will be confounded by patient demographics, healthcare systems and the hospitals involved, thus limiting the generalisability of the results.
- Due to the observational nature of the study, unmeasured confounders potentially influence the conclusions.

For peer review only

Introduction

A timely revascularization following presentation with an acute coronary syndrome (ACS) can mitigate loss of cardiac function and reduce morbidity and mortality [1,2]. Invasive procedures such as primary percutaneous coronary intervention (PCI) are central to the care of patients presenting with ST-elevation myocardial infarction (STEMI) [3] and guidelines also recommend an early invasive strategy for non-ST-elevation myocardial infarction (NSTEMI) cases [4,5].

Nevertheless, heterogeneity in hospital resource allocation, service provision and staffing levels across weekday and weekends can affect the delivery of optimal care, leading to studies of the so-called "weekend-effect" [6–8]. Higher rates of mortality have been indicated in patients admitted to hospital for an acute myocardial infarction (AMI) at a weekend, an evening or holiday period [7,9,10]. However, previous results in AMI patients are inconsistent [11–13]. An analysis of a multicentre registry highlighted that, despite weekend admission being associated with a delay in invasive therapy, this did not impact rates of adverse events [13]. Similarly, the "Get With the Guidelines-Coronary Artery Disease" database showed that despite longer door-to-balloon time, mortality rates were similar between 'out-of-hour' and 'in-hour' hospital admission [11].

Many previous studies have focused on outcome differences across weekend/ weekday admissions in STEMI patients, with limited data for patients with NSTEMI or unstable angina (NSTEMI/ UA). Service provision/ delivery, healthcare systems, deprivation, and population demographics vary both temporally and by geography, thereby confounding the analysis of a weekend-effect; even the definition of weekend or "out-of-hours" admission varies between populations. Such factors might explain, in part, the inconsistencies across previous studies. Therefore, the aim of this study was to examine the effect of weekend vs.

For peer review only - http://bmjopen.bmj.com/site/about/guidelines.xhtml

weekday admission on outcomes following presentation with STEMI or NSTEMI/ UA, in a contemporary, multicentre dataset of the UK.

Methods

MINAP Dataset

The Myocardial Ischaemia National Audit Project (MINAP) collects prospective data on the management of heart attacks in the UK [14]. Each centre is responsible for data entry into MINAP, based on agreed definitions and options for each variable. This study had access to data from three contributing centres, namely the Freeman Hospital (Newcastle-upon-Tyne), the Royal Stoke Hospital (Stoke-on-Trent) and the University Hospital of Wales (Cardiff). The dataset includes variables detailing patient characteristics, emergency response/ admission dates, processes of care, and outcomes within hospital discharge. Long-term mortality tracking was available from the Office for National Statistics for English patients and the Welsh Demographic Service for Welsh patients.

Study Design

This retrospective analysis included all patients who had a discharge diagnosis of an ACS and were sub-classified into ST elevation myocardial infarction, non-ST elevation myocardial infarction, or unstable angina. After such inclusion criteria, the STEMI sub-group was defined as those patients with an admission and/or discharge diagnosis of ST elevation Myocardial Infarction; all other patients meeting the discharge diagnosis inclusion criteria were included in the NSTEMI/UA sub-group.

Using hospital admission dates, patients were classified into weekend or weekday admission (reference group). In line with previous studies, weekends were defined as any admission between 12:00am Saturday and 11:59pm Sunday, with weekday defined as any admissions outside this time window [7–9]. The primary outcome measures were in-hospital

For peer review only - http://bmjopen.bmj.com/site/about/guidelines.xhtml

mortality, 30-day mortality and long-term survival. Secondary outcomes included the following process of care indicators: admission to a cardiology ward, length of stay (defined as whole number of days between admission date and discharge date), performance of coronary angiography/ echocardiography, time to coronary angiography (defined as number of hours between admission time and time of angiogram), and discharged on beta-blocker, ACE-I or statins. Note that any patient admitted and discharged on the same day had length of stay defined as zero. All the outcomes were analysed as a whole cohort (with adjustment for STEMI indication) and separately in the STEMI and NSTEMI/ UA sub-groups.

Statistical Analysis

For descriptive analysis, continuous variables were presented as means with standard deviations and compared using the t-test. Categorical variables were presented as frequencies of occurrence and compared using the chi-squared test. Patients with missing endpoint variables were excluded from the analysis of that endpoint. Other patient characteristic variables that were missing were assigned to the gender-matched median value for continuous variables or the reference category for categorical variables.

To control for potential differences in baseline covariates between weekend and weekday admission, propensity scores (PS) for being admitted at a weekend were calculated for each patient [15,16]. Although day of hospital admission is not a modifiable intervention, one could regard this in the context of differences in resource allocation between the weekend and weekday. Variables included in the logistic regression model to calculate the PS included all those given in **Table 1** and an admitting centre indicator. Patients were stratified into five strata using their PS, with the cut-off values determined by the quintiles of the PS distribution for all patients. Such PS stratification has been shown to remove 90% of the bias due to the covariates within the PS model [15,16].

For peer review only - http://bmjopen.bmj.com/site/about/guidelines.xhtml

Within each PS strata, binary outcomes (mortality, admission to cardiology ward, performance of coronary angiography/ echocardiography and prescription of medications) were compared directly between admission day groups (using logistic regression), with the odds ratios (ORs) pooled by the Mantel-Haenszel method [17]. For time-to-event outcomes (length of stay, time-to-angiography and long-term survival), the unadjusted survival curves were obtained using the Kaplan-Meier estimate and adjusted hazards ratios (HRs) were estimated using a Cox proportional hazards model. The Cox proportional hazards model included weekend indication as the covariate and was stratified by PS strata. The proportional hazards assumption of the weekend effect variable was checked by examining the Schoenfeld residuals against time; weekend-by-time interactions were included where necessary. We did not model length of stay beyond 50 days, or time-to-angiography beyond 7 days for NSTEMI/ UA and beyond 5 hours for STEMI. All estimates are reported with corresponding 95% confidence intervals (CIs).

R version 3.3.1 [18] was used for all statistical analyses. Graphical plots where made using the “ggplot2” package [19], and the “survival” package was used for time-to-event analyses [20,21].

Results

There were 18,166 admissions across the three centres between 1st January 2010 and 30th March 2016. This comprised 3,140 admissions in University Hospital of Wales, 7,023 admissions in Royal Stoke Hospital and 8,003 admissions in Freeman Hospital. Together, 461 admissions did not meet the discharge diagnosis inclusion criteria. Thus, the analysis sample size was 17,705 with n=9,322 admissions for a STEMI and n=7,887 admissions for an NSTEMI/ UA; the type of ACS was unknown in the remaining 496 records, which were removed from the STEMI and NSTEMI/ UA sub-group analyses.

For peer review only - http://bmjopen.bmj.com/site/about/guidelines.xhtml

Table 1 gives the patient baseline characteristics by weekend or weekday admission. Patients admitted on a weekday were older (p=0.001), were more likely female (p=0.028), and had higher rates of previous AMI (p=0.011), previous angina (p=0.002), cerebrovascular disease (p=0.010), asthma/chronic obstructive pulmonary disease (p=0.044) and previous PCI (p=0.014). Conversely, weekend patients were more likely to have elevated enzymes (p<0.001) or present with ST elevation (p<0.001), although Killip class did not significantly differ between admission groups. Importantly, rates of admission to a cardiologist were not different across weekend or weekday cohorts, with both being over 96% (p=0.216).

Mortality

Figure 1 shows hospital mortality rates by day of the week. There was no apparent trend in either overall hospital mortality or cardiac/ non-cardiac related deaths. In the whole cohort, the observed hospital mortality rate was 3.96% and 3.41% for weekday and weekend admission groups, respectively (OR 0.86, 95% CI: 0.71, 1.03) (**Table 2**). After PS stratification, the pooled OR was 0.82 (95% CI: 0.58, 1.09). Similar findings were found across STEMI and NSTEMI/ UA sub-groups (**Table 3**).

Overall one-, three- and five-year survival rates were 89.7%, 82.3% and 74.9%, respectively. As a whole cohort, there was no significant difference in the Kaplan-Meier survival curves between admission groups (log-rank test, p=0.569) (**Figure 2**). The PS adjusted HR from a Cox proportional hazards model for the whole cohort was 0.99 (95% CI: 0.90, 1.09) for weekend over weekday admission. In STEMI cases, the Kaplan-Meier curves were significantly different (log-rank test, p=0.032), but this was not observed for NSTEMI/ UA cases (log-rank test, p=0.103). After PS adjustment, long-term survival was not significantly different between weekday or weekend admissions, with a HR for STEMI cases of 0.94 (95% CI: 0.82, 1.07) and for NSTEMI/ UA cases of 1.02 (95% CI: 0.89, 1.18).

For peer review only - http://bmjopen.bmj.com/site/about/guidelines.xhtml

Admission Process

In the weekend group, 4045 of 4318 (93.7%) patients were admitted to either a cardiac ward or a coronary care unit, compared with 12548 of 13345 (94.0%) in the weekday group (**Table 2**). Rates of admission to a cardiac ward or coronary care unit were not significantly different between the weekend and weekday, with an adjusted OR of 0.97 (95% CI: 0.76, 1.23). In STEMI admissions the adjusted OR was 1.08 (95% CI: 0.67, 1.52) and in NSTEMI/ UA admissions was 0.93 (95% CI: 0.71, 1.20) (**Table 3**).

There was a significant difference in the Kaplan-Meier curves for length of stay between weekday and weekend admission for STEMI (log-rank p=0.001) and for NSTEMI/ UA patients (log-rank p<0.001) (**Supplementary Figure 1**). Weekend admission was associated with significantly lower hazards of being discharged within one-day (HR 0.72, 95% CI: 0.66, 0.78), but significantly higher hazards of being discharged between 1 and 4 days (HR 1.13, 95% CI: 1.08, 1.18) (**Table 4**). Similar findings were found when examining length of stay across STEMI and NSTEMI/ UA sub-groups (**Supplementary Table 1**).

Coronary Angiography

There was no significant difference in the proportion of patients receiving a coronary angiography between weekday and weekend admission (OR 0.91, 95% CI: 0.71, 1.13) (**Table 2**). In STEMI cases, 96.5% and 96.6% of patients underwent an angiography on a weekday and weekend, respectively (adjusted OR 0.95, 95% CI: 0.62, 1.36). Similarly, NSTEMI/ UA patients did not have significantly different odds of undergoing a coronary angiography by weekend admission, after PS stratification adjustment (**Table 3**).

Within the group of patients who underwent a coronary angiography, we assessed the time-to-angiography where time of the angiogram was also available (n=7,284 STEMI and n=5,705 NSTEMI/ UA). There was a significant difference in the cumulative event rates for time-to-angiography for NSTEMI/ UA (log-rank p<0.001), and for STEMI cases (log-rank

For peer review only - http://bmjopen.bmj.com/site/about/guidelines.xhtml

For peer review only

p=0.010) (**Figure 3**). In NSTEMI/ UA cases, the number of patients receiving angiography within 24 hours differed significantly by weekend admission groups, with similar findings at 72 hours (**Supplementary Table 2**). For instance, 88.9% of weekend NSTEMI/ UA admissions received an angiography within 72 hours, compared with 91.1% of weekday NSTEMI/ UA admissions (p=0.028). After PS adjustment, the time dependent Cox proportional hazards model indicated that weekend NSTEMI/ UA patients were less likely to undergo a coronary angiography within 2 hours (HR 0.54, 95% CI: 0.42, 0.69) and between 2-24 hours (HR 0.71, 95% CI: 0.65, 0.77), with the observed mean time-to-angiography being approximately 3 hours longer within the weekend cohort by 24 hours (**Table 5**). Baseline NSTEMI/ UA patient characteristics between those undergoing angiography within 24 hours and those after 24 hours are given in **Supplementary Table 3**. NSTEMI/ UA patients who underwent angiography within 24 hours were more likely to have an ACS admission diagnosis (p<0.001), previous angina (p=0.024), hypercholesterolemia (p<0.001) or a family history of coronary heart disease (p<0.001), and were significantly more likely to be admitted to a cardiologist (p<0.001) compared with those over 24 hours.

In STEMI patients, after PS adjustment the Cox proportional hazards model indicated that there was a slight delay for weekend admission within 1 hour (HR 0.89, 95% CI: 0.84, 0.94) (**Table 5**). Specifically, between 0 and 1 hour, the observed mean time-to-angiography was 0.55 hours and 0.57 hours for weekday and weekend admission, respectively. After 1 hour, there was no significant difference in time-to-angiography between weekend or weekday STEMI admissions after PS adjustment (**Table 5**). Similarly, we examined door-to-balloon time in those STEMI patients who underwent primary PCI as the initial reperfusion treatment and where time of reperfusion was also available. Here, door-to-balloon time was longer than 90-minutes (as recommended by the American College of Cardiology) in 6.95% of weekday admissions, compared with 9.12% of weekend admissions (p=0.004). Likewise,

For peer review only - http://bmjopen.bmj.com/site/about/guidelines.xhtml

the European Society of Cardiology guideline of door-to-balloon time within 120 minutes was exceeded significantly more in weekend admissions (4.13%) compared with weekday admissions (2.96%) (p=0.02).

Echocardiography

At the weekend, 54.9% of patients underwent an echocardiogram, compared with 51.3% of weekday admissions, with both the unadjusted and PS adjusted ORs being significantly different from one (**Table 2**). Specifically, the pooled OR from the PS stratification was 1.16 (95% CI: 1.01, 1.31), indicating that patients admitted over the weekend had 16% greater odds of undergoing an echocardiography compared with those admitted during the week.

Prescribed Medication on discharge

The unadjusted analysis of both prescription of beta-blocker on discharge and prescription of ACE-I on discharge indicated significantly increased odds for those admitted at the weekend (**Table 2**). However, these findings were not significant after adjusting for PS. The crude proportions of statins on discharge were similar across the two groups, at 95.5% for both weekday and weekend admission (adjusted OR 0.96, 95% CI: 0.68, 1.24).

Discussion

The findings of the current study can be summarised as follows: 1) in current UK practice, patients admitted to 3 tertiary cardiac centres during weekends do not present with a higher baseline risk than weekday patients; 2) there was no difference in short- or long-term survival between weekends and weekday groups; 3) for NSTEMI patients there was a delay in angiography for patients admitted at a weekend; 4) other metrics of care quality such as admission under a cardiologist, performance of echocardiography and discharge medications were not adversely affected by a weekend admission.

For peer review only - http://bmjopen.bmj.com/site/about/guidelines.xhtml

The lack of a weekend effect in the treatment of an ST elevation myocardial infarction in a contemporary UK cohort is not surprising. The most recently published MINAP data for 2013/14 demonstrates that for the whole of the UK, 98.5% of patients with ST elevation received primary PCI, with few patients receiving thrombolysis [22]. Amongst its many benefits, with respect to day of the week, a primary PCI pathway is a consultant-led and consultant-delivered service, which is undertaken regardless of the time of day, or day of the week. Therefore, STEMI treatment in the UK is treated on a 24/7 basis and unless the patients admitted at a weekend are of a higher-risk profile, then it would not be expected that a weekend admission would be disadvantageous to their outcomes.

However, pathways for the treatment of NSTEMI/ UA are markedly different in the UK to those for STEMI. The findings of the current study identify differences in patient treatment with respect to their timings, but not regarding patient survival. The lack of a mortality difference for a weekend NSTEMI admission may, in part, be due to the high percentage of cases admitted under the care of a cardiologist regardless of day of admission (>96% for both cohorts). Consequently, the patients may well have received optimal care in a timely fashion. Crucially, review by a cardiologist early in a patient's admission can identify patients who are unstable or deemed to be high-risk NSTEMI patients and these can be prioritised and fast-tracked to the catheter-lab along a pathway similar to primary PCI. Early identification of high-risk NSTEMI patients by the admitting cardiologist and performing coronary angiography within 2 hours, regardless of the day of the week, can ensure that this risk is ameliorated by invasive management [23]. Indeed, several UK centres have developed direct transfer protocols to reduce time-to-angiography for patients with NSTEMI (the so-called HAC-X group) [24]. The optimal timing of angiography is less clear for intermediate-risk NSTEMI cases, with a lack of consistency in published trials [25–27]. In a recent meta-analysis of 10 time-to-angiography trials, there was no difference in mortality between

For peer review only - http://bmjopen.bmj.com/site/about/guidelines.xhtml

patients receiving angiography early (within 24-hours) or late (24-86 hours) [28]. Therefore, although the European Society of Cardiology guidelines suggest that the high-risk cohort receive angiography within 24 hours, they recommend that the intermediate-risk cohort receive angiography within 72 hours [5]. In the current study, the proportion of NSTEMI/ UA patients receiving angiography within both 24 and 72 hours was significantly lower for weekend admissions compared with weekday admissions.

Although the current study does not demonstrate a weekend-effect on patient outcomes, it does demonstrate an effect on length of stay, which is increasingly used as an endpoint to monitor quality of service. The HAC-X group demonstrated that a direct transfer protocol for angiography in NSTEMI patients reduced the length of stay by six days per admission [24]. Therefore, although 7 day working (and with it angiography) may not improve patient survival after an ACS, it is likely to significantly reduce their length of stay. A prolonged hospital admission has been closely associated with iatrogenic complications such as infection and venous thrombosis [29,30]. Therefore, as well as having significant implications on bed utilisation, efficiency and costs, performing angiography on NSTEMI patients at the weekend is likely to improve patients' experience and reduce their potential hospital acquired morbidity. Speculatively, the longer length of stay for weekend admissions might explain the finding of higher rates of echocardiograms performed at weekends (or *vice versa*).

Several factors might explain the differences in the results of previous studies with the current one. Firstly, the data analysed in the current study is derived from the period 2010-2016 and hence reflects contemporary ACS management. Current medical practice has evolved greatly over the last decade. Therefore, the findings of older studies will not necessarily be expected to be relevant or similar to a more contemporary cohort [6–8]. Secondly, the three centres involved in the current study are large university teaching

For peer review only - http://bmjopen.bmj.com/site/about/guidelines.xhtml

For peer review only

hospitals with a daily review of patients by a senior cardiologist, and 24/7 access to echocardiography and cardiac catheterisation. Therefore, data that includes significant numbers of admissions to smaller hospitals without 24/7 cardiology services might lead to differing outcomes for patients admitted during the weekends compared to a weekday. Finally, differing service provision, patient populations, demographics and healthcare systems might also lead to conflicting results of previous studies with the current study.

The strengths of the current study are that it is an analysis of contemporary practice, the data is derived from a robust national database, and the three centres involved are large tertiary interventional centres with high numbers of patients. However, as with any retrospective analysis there are limitations and the robustness of the conclusions are directly related to the quality of data entered. Unmeasured confounders inherent in observational studies of this nature potentially influence any conclusions. Additionally, this study included only those patients with a discharge diagnosis of STEMI or NSTEMI/ UA; consequently, outcomes might differ if the cohort was defined based solely on admission diagnosis. Finally, our findings might not generalise to other hospitals or healthcare systems due to heterogeneity in patient demographics, the healthcare system, and the hospitals involved.

Conclusions

In regional, university cardiac centres, with specialist cardiology services in the UK, ACS patients had similar mortality and process of care when admitted at a weekend compared with a weekday. Delays to time-to-angiography occurred in NSTEMI/ UA patients admitted at the weekend compared to weekdays, but this did not influence survival.

For peer review only - http://bmjopen.bmj.com/site/about/guidelines.xhtml

For peer review only

Funding Statement

This work was funded by the Medical Research Council through the Health e-Research Centre, University of Manchester [MR/K006665/1] and a grant through the North Staffordshire Heart Committee.

Competing Interests Statement

The authors have no conflict of interest.

Author Contributions

TM, AGZ, MAM contributed to the acquisition of the data for the analysis. GPM, TK, MS and MAM made substantial contributions to the concept of the work in addition to performing the analysis. GPM and TK drafted the initial version of the manuscript. GPM, TK, MS, RA, AG, AJ, CSK, DB, GH, AGZ and MAM interpreted the results, revised the paper critically for important intellectual content and approved the final version of the paper. All authors agreed to be accountable for all aspects of the work.

Data sharing statement

The statistical code used to analyse the data is available upon reasonable request by emailing MAM (corresponding author).

Ethics

The National Institute for Cardiovascular Outcomes Research (NICOR) which includes the MINAP database (Ref: NIGB: ECC 1-06 (d)/2011) has support under section 251 of the National Health Service Act 2006 to use patient information for medical research without informed consent. Further ethical approval was not required under current National Health Service research governance arrangements, as all data analysed in the study was pseudonymised and contained no patient identifiable information.

For peer review only - http://bmjopen.bmj.com/site/about/guidelines.xhtml

Figure Legends

Figure 1: In-hospital mortality rates by the day of arrival decomposed into cardiac and non-cardiac related mortality.

Figure 2: Long-term survival following weekend versus weekday admission as a whole cohort and across STEMI and NSTEMI/ UA sub-groups.

Figure 3: Cumulative event rate for time to coronary angiography following weekend versus weekday admission across STEMI and NSTEMI/ UA sub-groups.

For peer review only

For peer review only - http://bmjopen.bmj.com/site/about/guidelines.xhtml

Tables

Table 1: Baseline Summary

Variable	Whole Cohort [n=17705]	Weekday Admission [n=13378]	Weekend Admission [n=4327]	p-value	Missing (% of whole cohort)
Age at Admission, mean (SD)	66.0 (13.0)	66.2 (13.0)	65.5 (13.1)	0.001	7 (0.04)
Male, n (%)	12530 (70.8)	9410 (70.3)	3120 (72.1)	0.028	0 (0.00)
Caucasian, n (%)	12283 (69.4)	9360 (70.0)	2923 (67.6)	0.003	4988 (28.2)
Admission Diagnosis					0 (0.00)
Acute coronary syndrome, n (%)	15427 (87.1)	11689 (87.4)	3738 (86.4)	0.097	
Chest pain unknown cause, n (%)	1759 (9.94)	1316 (9.84)	443 (10.2)	0.461	
Other, n (%)	519 (2.93)	373 (2.79)	146 (3.37)	0.053	
Previous AMI, n (%)	3768 (21.3)	2907 (21.7)	861 (19.9)	0.011	109 (0.62)
Previous Angina, n (%)	3968 (22.4)	3073 (23.0)	895 (20.7)	0.002	282 (1.59)

For peer review only - http://bmjopen.bmj.com/site/about/guidelines.xhtml

Hypertension, n (%)	9392 (53.0)	7153 (53.5)	2239 (51.7)	0.050	255 (1.44)
Hypercholesterolemia, n (%)	7166 (40.5)	5458 (40.8)	1708 (39.5)	0.127	574 (3.24)
PVD, n (%)	809 (4.57)	626 (4.68)	183 (4.23)	0.234	85 (0.48)
Cerebrovascular Disease, n (%)	1270 (7.17)	998 (7.46)	272 (6.29)	0.010	83 (0.47)
Asthma or COPD, n (%)	2717 (15.3)	2095 (15.7)	622 (14.4)	0.044	114 (0.64)
Chronic Renal Failure, n (%)	691 (3.90)	527 (3.94)	164 (3.79)	0.693	143 (0.81)
Heart Failure, n (%)	508 (2.87)	379 (2.83)	129 (2.98)	0.649	159 (0.90)
Enzymes Elevated, n (%)	16378 (92.5)	12304 (92.0)	4074 (94.2)	<0.001	634 (3.58)
Previous/ Current Smoker, n (%)	11892 (67.2)	9021 (67.4)	2871 (66.4)	0.195	550 (3.11)
Cholesterol, mean (SD)	4.77 (1.35)	4.76 (1.35)	4.82 (1.38)	0.015	4590 (25.9)
Diabetes					126 (0.71)
Dietary control, n (%)	609 (3.44)	464 (3.47)	145 (3.35)	0.749	
Oral medicine, n (%)	1870 (10.6)	1394 (10.4)	476 (11.0)	0.293	
Insulin, n (%)	721 (4.07)	571 (4.27)	150 (3.47)	0.023	
Insulin and medication, n (%)	297 (1.68)	224 (1.67)	73 (1.69)	0.999	
Previous PCI, n (%)	2124 (12.0)	1651 (12.3)	473 (10.9)	0.014	17 (0.10)

For peer review only - http://bmjopen.bmj.com/site/about/guidelines.xhtml

Previous CABG, n (%)	959 (5.42)	732 (5.47)	227 (5.25)	0.595	4 (0.02)
Systolic BP, mean (SD)	135.3 (27.7)	135.1 (27.5)	135.8 (28.3)	0.219	2574 (14.5)
Heart Rate, mean (SD)	76.8 (19.3)	76.5 (19.1)	77.5 (19.8)	0.006	2257 (12.7)
Admitting Consultant					52 (0.29)
Cardiologist, n (%)	17093 (96.5)	12929 (96.6)	4164 (96.2)	0.216	
Other general physician, n (%)	445 (2.51)	325 (2.43)	120 (2.77)	0.230	
Other, n (%)	115 (0.65)	88 (0.66)	27 (0.62)	0.895	
Beta Blocker, n (%)	4670 (26.4)	3555 (26.6)	1115 (25.8)	0.306	617 (3.48)
Statin, n (%)	6997 (39.5)	5360 (40.1)	1637 (37.8)	0.009	630 (3.56)
Glucose, mean (SD)	8.60 (4.60)	8.55 (4.61)	8.76 (4.58)	0.015	3044 (17.2)
Height, mean (SD)	170.1 (9.76)	170.0 (9.81)	170.5 (9.57)	0.011	3514 (19.8)
Weight, mean (SD)	81.9 (18.7)	81.8 (18.8)	82.0 (18.3)	0.624	2571 (14.5)
Family History of CHD, n (%)	8287 (46.8)	6273 (46.9)	2014 (46.5)	0.705	1939 (11.0)
Creatinine, mean (SD)	95.7 (64.6)	95.5 (63.3)	96.3 (68.4)	0.502	266 (1.50)
Haemoglobin, mean (SD)	136.6 (19.1)	136.3 (19.2)	137.5 (18.8)	<0.001	195 (1.10)

For peer review only

For peer review only - http://bmjopen.bmj.com/site/about/guidelines.xhtml

Killip class					6919 (39.1)
No evidence of Heart Failure, n (%)	9103 (51.4)	6918 (51.7)	2185 (50.5)	0.170	
Basal Crepitations, n (%)	811 (4.58)	590 (4.41)	221 (5.11)	0.062	
Pulmonary Oedema, n (%)	600 (3.39)	453 (3.39)	147 (3.40)	0.999	
Cardiogenic Shock, n (%)	266 (1.50)	195 (1.46)	71 (1.64)	0.430	
Cardiac Arrest, n (%)	1493 (8.43)	1106 (8.27)	387 (8.94)	0.174	0 (0.00)
Thienopyridene, n (%)	15632 (88.3)	11774 (88.0)	3858 (89.2)	0.043	109 (0.62)
STEMI, n (%)	9322 (52.7)	6781 (50.7)	2541 (58.7)	<0.001	496 (2.80)

AMI: Acute Myocardial Infarction; BP: Blood Pressure; CABG: Coronary Artery Bypass Graft; CHD: Coronary Heart Disease; COPD: Chronic Obstructive Pulmonary Disease; LVEF: Left Ventricular Ejection Fraction; MI: Myocardial Infarction; PCI: Percutaneous Coronary Intervention; PVD: Peripheral Vascular Disease; STEMI: ST Segment Elevation Myocardial Infarction

For peer review only

Table 2: Event numbers, unadjusted odds ratios (OR) and pooled OR across PS strata. Bold item indicate significant effects at the 5% level.

**Outcome**	**Weekday Admission [n=13378]**	**Weekend Admission [n=4327]**	**Unadjusted OR (95% CI)**	**PS Adjusted OR (95% CI)**
Hospital Mortality	529/13354 (3.96%)	147/4312 (3.41%)	0.86 (0.71, 1.03)	0.82 (0.58, 1.09)
30-day Mortality	553/10874 (5.09%)	182/3545 (5.13%)	1.01 (0.85, 1.20)	0.96 (0.72, 1.28)
Admission to Cardiology Ward	12548/13345 (94.0%)	4045/4318 (93.7)	0.94 (0.82, 1.09)	0.97 (0.76, 1.23)
Coronary Angiography	12401/13265 (93.5%)	3987/4288 (93.0%)	0.92 (0.81, 1.06)	0.91 (0.71, 1.13)
Echocardiography	5772/11262 (51.3%)	2014/3666 (54.9%)	**1.16 (1.08, 1.25)**	**1.16 (1.01, 1.31)**
Discharged on Beta-blocker	10320/12181 (84.7%)	3404/3943 (86.3%)	**1.14 (1.03, 1.26)**	1.06 (0.88, 1.26)
Discharged on ACE-I	10801/12109 (89.2%)	3552/3930 (90.4%)	**1.14 (1.01, 1.29)**	1.09 (0.88, 1.34)
Discharged on Statins	11590/12131 (95.5%)	3764/3942 (95.5%)	0.99 (0.83, 1.18)	0.96 (0.68, 1.24)

For peer review only

Table 3: Event numbers and pooled odds ratios (OR) across PS strata by STEMI and NSTEMI/ UA sub-groups.

	STEMI (n=9322)			NSTEMI/ UA (n=7887)		
**Outcome**	**Weekday Admission [n=6781]**	**Weekend Admission [n=2541]**	**PS Adjusted OR (95% CI)**	**Weekday Admission [n=6190]**	**Weekend Admission [n=1697]**	**PS Adjusted OR (95% CI)**
Hospital Mortality	379/6774 (5.59%)	110/2533 (4.34%)	0.83 (0.58, 1.15)	137/6175 (2.22%)	36/1691 (2.13%)	0.82 (0.45, 1.38)
30-day Mortality	379/5633 (6.73%)	132/2123 (6.22%)	0.99 (0.69, 1.35)	157/4913 (3.20%)	45/1352 (3.33%)	0.88 (0.53, 1.41)
Admission to Cardiology Ward	6486/6763 (95.9%)	2442/2533 (96.4%)	1.08 (0.67, 1.52)	5673/6176 (91.9%)	1518/1696 (89.5%)	0.93 (0.71, 1.20)
Coronary Angiography	6512/6749 (96.5%)	2445/2530 (96.6%)	0.95 (0.62, 1.36)	5532/6113 (90.5%)	1465/1669 (87.8%)	0.92 (0.70, 1.15)
Echocardiography	3241/6085 (53.3%)	1267/2210 (57.3%)	1.17 (0.98, 1.38)	2377/4796 (49.6%)	714/1374 (52.0%)	1.16 (0.93, 1.42)
Discharged on Beta-blocker	5226/5976 (87.4%)	2018/2261 (89.3%)	1.08 (0.83, 1.39)	4838/5846 (82.8%)	1328/1600 (83.0%)	1.03 (0.80, 1.32)

For peer review only - http://bmjopen.bmj.com/site/about/guidelines.xhtml

Discharged on ACE-I	5529/5940 (93.1%)	2109/2254 (93.6%)	1.04 (0.72, 1.41)	4998/5815 (86.0%)	1374/1594 (86.2%)	1.13 (0.83, 1.41)
Discharged on Statins	5772/5951 (97.0%)	2187/2260 (96.8%)	0.89 (0.50, 1.32)	5481/5824 (94.1%)	1498/1600 (93.6%)	1.01 (0.68, 1.41)

For peer review only

Table 4: Number of patients discharged within each time-window and PS adjusted hazard ratios (HR) from the time-dependent Cox proportional hazards models for length of stay, as a whole cohort. Bold items indicate significant effects at the 5% level.

Length of stay	Discharged, n (%) Weekday Cohort	Discharged, n (%) Weekend Cohort	PS adjusted HR (95% CI)
0 – 1 day	2888 (21.7%)	636 (14.7%)	**0.72 (0.66, 0.78)**
1 - 4 days	6731 (64.5%)	2595 (70.5%)	**1.13 (1.08, 1.18)**
4 - 50 days	3639 (98.2%)	1063 (98.1%)	0.97 (0.90, 1.04)

For peer review only

For peer review only - http://bmjopen.bmj.com/site/about/guidelines.xhtml

Table 5: PS adjusted hazard ratios (HR) from time-dependent Cox proportional hazards models for time to coronary angiography in STEMI and NSTEMI/ UA patients. Bold items indicate significant effects at the 5% level.

	**STEMI**		
**Time to coronary angiography***	**Mean time-to-angiography Weekday, (hours)**	**Mean time-to-angiography Weekend, (hours)**	**PS adjusted HR (95% CI)**
0 - 1 hour	0.55	0.57	**0.89 (0.84, 0.94)**
1 - 2 hours	1.52	1.53	0.99 (0.87, 1.11)
2 – 5 hours	3.27	3.11	1.25 (1.00, 1.56)
	**NSTEMI/ UA**		
0 - 2 hours	1.89	1.94	**0.54 (0.42, 0.69)**
2 - 24 hours	15.7	18.3	**0.71 (0.65, 0.77)**
24 hours - 4 days	56.2	53.9	**1.22 (1.09, 1.36)**
4 - 7 days	139.9	137.0	0.94 (0.68, 1.29)

**: The time-windows for the time-dependent Cox proportional hazard model were made to satisfy the proportionality assumption throughout time. In the case of NSTEMI/ UA, we selected 24 hours as one of the time cut-offs to reflect the ESC guidelines for high-risk patients.*

For peer review only

For peer review only - http://bmjopen.bmj.com/site/about/guidelines.xhtml

References

1 Berger PB, Ellis SG, Holmes DRJ, *et al.* Relationship between delay in performing direct coronary angioplasty and early clinical outcome in patients with acute myocardial infarction: results from the global use of strategies to open occluded arteries in Acute Coronary Syndromes (GUSTO-IIb) trial. *Circulation* 1999;**100**:14–20.

2 McNamara RL, Wang Y, Herrin J, *et al.* Effect of Door-to-Balloon Time on Mortality in Patients With ST-Segment Elevation Myocardial Infarction. *J Am Coll Cardiol* 2006;**47**:2180–6. doi:10.1016/j.jacc.2005.12.072

3 O'Gara PT, Kushner FG, Ascheim DD, *et al.* 2013 ACCF/AHA Guideline for the Management of ST-Elevation Myocardial Infarction. *J Am Coll Cardiol* 2013;**61**:e78–140. doi:10.1016/j.jacc.2012.11.019

4 Amsterdam EA, Wenger NK, Brindis RG, *et al.* 2014 AHA/acc guideline for the management of patients with Non-ST-Elevation acute coronary syndromes: A report of the American College of Cardiology/American Heart Association Task Force on Practice Guidelines. *J Am Coll Cardiol* 2014;**64**:e139–228. doi:10.1016/j.jacc.2014.09.017

5 Roffi M, Patrono C, Collet J-P, *et al.* 2015 ESC Guidelines for the management of acute coronary syndromes in patients presenting without persistent ST-segment elevation. *Eur Heart J* 2016;**37**:267–315. doi:10.1093/eurheartj/ehv320

6 Bell CM, Redelmeier DA. Mortality among Patients Admitted to Hospitals on Weekends as Compared with Weekdays. *N Engl J Med* 2001;**345**:663–8. doi:10.1056/NEJMsa003376

7 Kostis WJ, Demissie K, Marcella SW, *et al.* Weekend versus Weekday Admission and Mortality from Myocardial Infarction. *N Engl J Med* 2007;**356**:1099–109.

For peer review only

For peer review only - http://bmjopen.bmj.com/site/about/guidelines.xhtml

doi:10.1056/NEJMoa063355

8 Cram P, Hillis SL, Barnett M, *et al.* Effects of weekend admission and hospital teaching status on in-hospital mortality. *Am J Med* 2004;**117**:151–7. doi:10.1016/j.amjmed.2004.02.035

9 Khoshchehreh M, Groves EM, Tehrani D, *et al.* Changes in mortality on weekend versus weekday admissions for Acute Coronary Syndrome in the United States over the past decade. *Int J Cardiol* 2016;**210**:164–72. doi:10.1016/j.ijcard.2016.02.087

10 Sorita A, Ahmed A, Starr SR, *et al.* Off-hour presentation and outcomes in patients with acute myocardial infarction: systematic review and meta-analysis. *BMJ* 2014;**348**:f7393–f7393. doi:10.1136/bmj.f7393

11 Jneid H, Fonarow GC, Cannon CP, *et al.* Impact of Time of Presentation on the Care and Outcomes of Acute Myocardial Infarction. *Circulation* 2008;**117**:2502–9. doi:10.1161/CIRCULATIONAHA.107.752113

12 Berger A, Stauffer J-C, Radovanovic D, *et al.* Comparison of In-Hospital Mortality for Acute Myocardial Infarction in Switzerland With Admission During Routine Duty Hours Versus Admission During Out of Hours (Insight Into the AMIS Plus Registry). *Am J Cardiol* 2008;**101**:422–7. doi:10.1016/j.amjcard.2007.09.092

13 Ryan JW, Peterson ED, Chen AY, *et al.* Optimal timing of intervention in non-ST-segment elevation acute coronary syndromes: Insights from the CRUSADE (Can Rapid risk stratification of Unstable angina patients Suppress ADverse outcomes with Early implementation of the ACC/AHA guidelines) regist. *Circulation* 2005;**112**:3049–57. doi:10.1161/CIRCULATIONAHA.105.582346

14 Mamas MA, Kwok CS, Kontopantelis E, *et al.* Relationship Between Anemia and Mortality Outcomes in a National Acute Coronary Syndrome Cohort: Insights From the UK Myocardial Ischemia National Audit Project Registry. *J Am Heart Assoc*

For peer review only

2016;**5**:e003348. doi:10.1161/JAHA.116.003348

15 Rosenbaum PR, Rubin DB. The Central Role of the Propensity Score in Observational Studies for Causal Effects. *Biometrika* 1983;**70**:41–55.

16 Rosenbaum PR, Rubin DB. Reducing Bias in Observational Studies Using Subclassification on the Propensity Score. *J Am Stat Assoc* 1984;**79**:516–24.

17 Mantel N, Haenszel W. Statistical aspects of the analysis of data from retrospective studies of disease. *J Natl Cancer Inst* 1959;**22**:719–48.

18 R Core Team R. R: A Language and Environment for Statistical Computing. R Found. Stat. Comput. 2017. doi:10.1007/978-3-540-74686-7

19 Wickham H. *ggplot2: elegant graphics for data analysis*. Springer New York 2009.

20 Therneau T. A Package for Survival Analysis in S. Published Online First: 2015.http://cran.r-project.org/package=survival

21 Therneau T, Grambsch P. *Modeling Survival Data: Extending the Cox Model*. New York: : Springer 2000.

22 Weston C, Gavalova L, Whittaker T, *et al.* Myocardial Ischaemia National Audit Project: How the NHS cares for patients with heart attack. 2014.https://www.ucl.ac.uk/nicor/audits/minap/documents/annual_reports/minap-public-report-2014

23 de Araujo Goncalves P. TIMI, PURSUIT, and GRACE risk scores: sustained prognostic value and interaction with revascularization in NSTE-ACS. *Eur Heart J* 2005;**26**:865–72. doi:10.1093/eurheartj/ehi187

24 Gallagher SM, Lovell MJ, Jones DA, *et al.* Does a ‘direct’ transfer protocol reduce time to coronary angiography for patients with non-ST-elevation acute coronary syndromes? A prospective observational study. *BMJ Open* 2014;**4**:e005525. doi:10.1136/bmjopen-2014-005525

For peer review only - http://bmjopen.bmj.com/site/about/guidelines.xhtml

25 Neumann F-J, Kastrati A, Pogatsa-Murray G, *et al.* Evaluation of Prolonged Antithrombotic Pretreatment (‘Cooling-Off’ Strategy) Before Intervention in Patients With Unstable Coronary Syndromes. *JAMA* 2003;**290**:1593–9. doi:10.1001/jama.290.12.1593

26 Montalescot G, Cayla G, Collet J-P, *et al.* Immediate vs Delayed Intervention for Acute Coronary Syndromes. *JAMA* 2009;**302**:947. doi:10.1001/jama.2009.1267

27 van ’t Hof AWJ, de Vries ST, Dambrink J-HE, *et al.* A comparison of two invasive strategies in patients with non-ST elevation acute coronary syndromes: results of the Early or Late Intervention in unStable Angina (ELISA) pilot study. 2b/3a upstream therapy and acute coronary syndromes. *Eur Heart J* 2003;**24**:1401–5. doi:10.1016/S0195-668X(03)00259-8

28 Milasinovic D, Milosevic A, Marinkovic J, *et al.* Timing of invasive strategy in NSTE-ACS patients and effect on clinical outcomes: A systematic review and meta-analysis of randomized controlled trials. *Atherosclerosis* 2015;**241**:48–54. doi:10.1016/j.atherosclerosis.2015.04.808

29 Sbrojavacca R, Pietrangelo A, Fenoglio L, *et al.* Reducing the risk of hospital admission: A call to action from the Italian Society of Internal Medicine. *Eur J Intern Med* 2015;**26**:476–7. doi:10.1016/j.ejim.2015.06.003

30 Lilly CM, Cody S, Zhao H, *et al.* Hospital mortality, length of stay, and preventable complications among critically ill patients before and after tele-ICU reengineering of critical care processes. *JAMA* 2011;**305**:2175–83. doi:10.1001/jama.2011.697

For peer review only

For peer review only - http://bmjopen.bmj.com/site/about/guidelines.xhtml

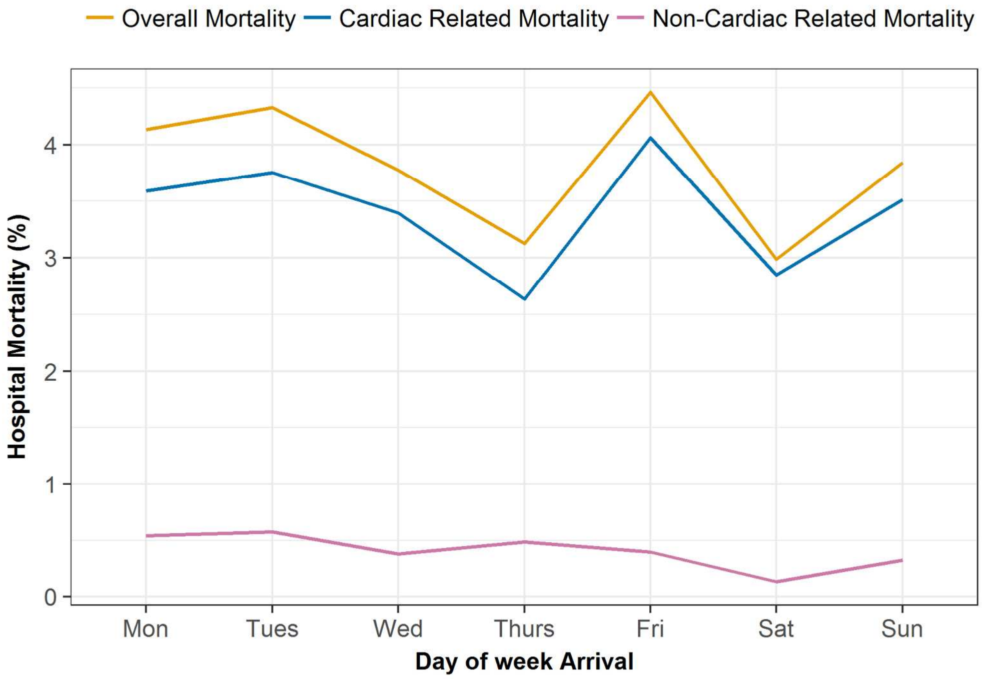

Figure 1: In-hospital mortality rates by the day of arrival decomposed into cardiac and non-cardiac related mortality.

177x127mm (300 x 300 DPI)

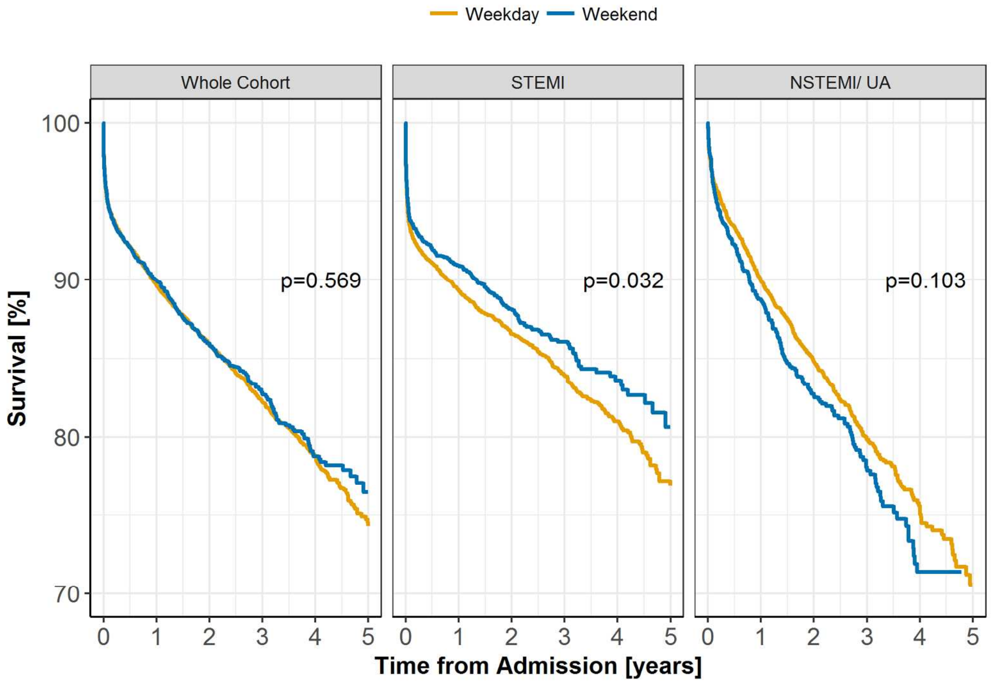

Figure 2: Long-term survival following weekend versus weekday admission as a whole cohort and across STEMI and NSTEMI/ UA sub-groups.

177x127mm (300 x 300 DPI)

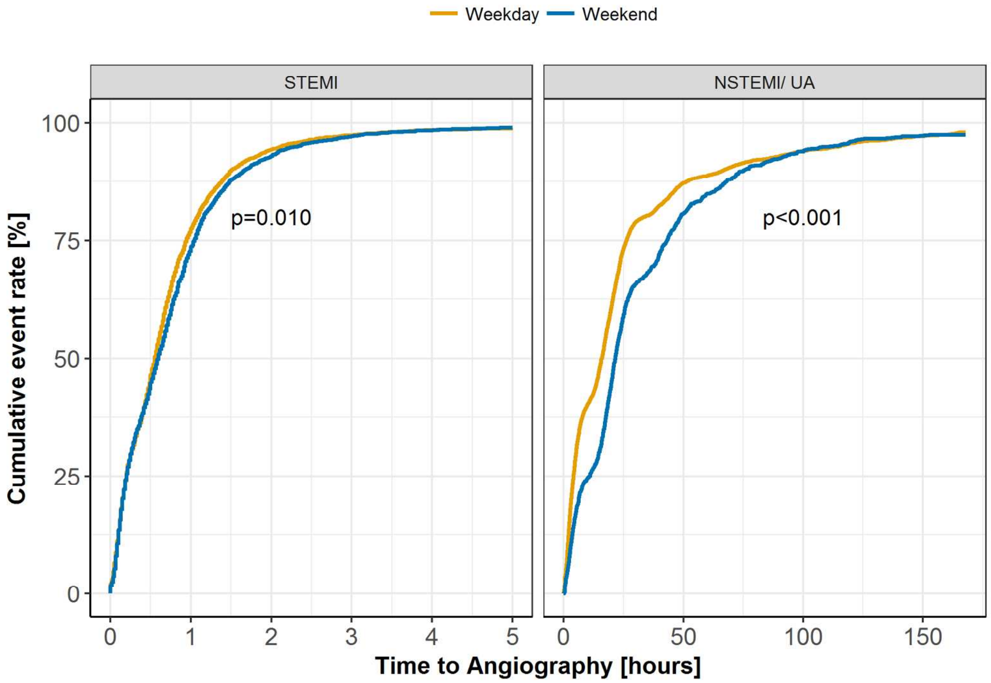

Figure 3: Cumulative event rate for time to coronary angiography following weekend versus weekday admission across STEMI and NSTEMI/ UA sub-groups.

177x127mm (300 x 300 DPI)

For peer review only - http://bmjopen.bmj.com/site/about/guidelines.xhtml

Supplementary Material

Effect of Weekend Admission on Process of Care and Clinical Outcomes for the Management of Acute Coronary Syndromes

Glen P. Martin*; Tim Kinnaird*; Matthew Sperrin; Richard Anderson; Amr Gamal; Avais Jabbar; Chun Shing Kwok; Diane Barker; Grant Heatlie; Azfar G. Zaman; Mamas A. Mamas

* Joint first authors: these authors contributed equally to manuscript

Supplementary Figures

Supplementary Figure 1: Cumulative event rate for length of stay following weekend versus weekday admission across STEMI and NSTEMI/ UA subgroups.

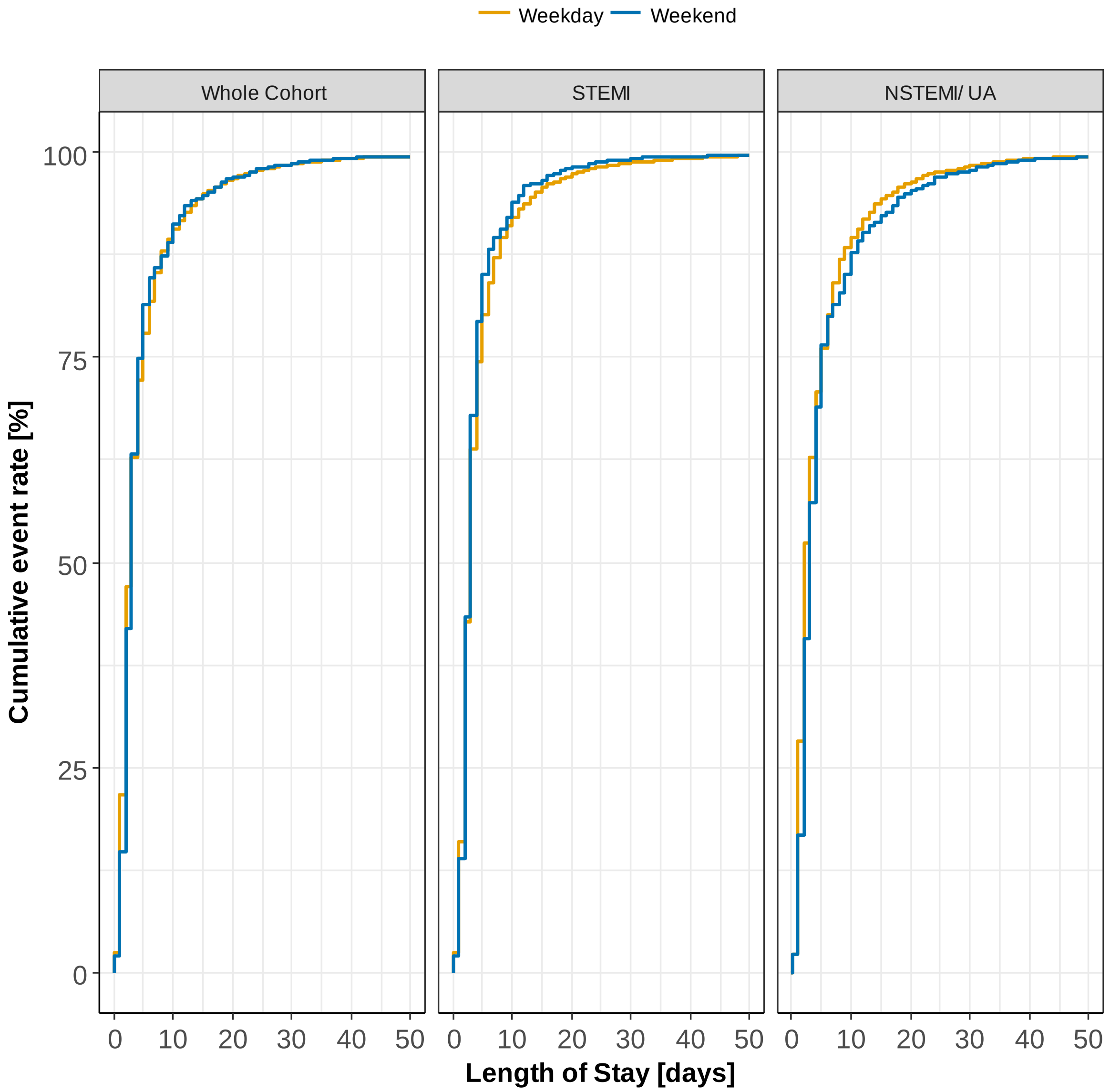

Supplementary Tables

Supplementary Table 1: Number of patients discharged within each time-window and PS adjusted hazard ratios (HR) from the time-dependent Cox proportional hazards models for length of stay, across STEMI and NSTEMI/ UA subgroups. Bold items indicate significant effects at the 5% level.

	**STEMI**		
**Length of stay**	**Discharged, n (%) Weekday Cohort**	**Discharged, n (%) Weekend Cohort**	**HR (95% CI)**
0 – 1 day	1074 (15.9%)	352 (13.9%)	**0.85 (0.75, 0.96)**
1 - 4 days	3953 (69.5%)	1659 (76.0%)	**1.13 (1.06, 1.19)**
4 - 50 days	1702 (98.3%)	513 (98.1%)	1.04 (0.94, 1.15)
	**NSTEMI/ UA**		
0 – 1 day	1733 (28.1%)	283 (16.7%)	**0.65 (0.57, 0.74)**
1 - 4 days	2628 (59.3%)	884 (62.7%)	**1.12 (1.04, 1.21)**
4 - 50 days	1770 (98.1%)	515 (97.9%)	0.93 (0.84, 1.03)

For peer review only

Supplementary Table 2: Proportions of NSTEMI/ UA patients receiving angiography within 24 and 72 hours across weekend admission groups.

Time-to-angiography	Weekday NSTEMI/ UA	Weekend NSTEMI/ UA	p-value
Within 24 hours	3242/ 4529 (71.6%)	662/ 1176 (56.3%)	<0.001
Within 72 hours	4125/ 4529 (91.1%)	1046/ 1176 (88.9%)	0.028

For peer review only

Supplementary Table 3: NSTEMI/UA patient characteristics across those that underwent coronary angiography within 24 hours compared with those after 24 hours.

Variable	Angiography≤24 hours [n=3902]	Angiography>24 hours [n=1802]	p-value
Age at Admission, mean (SD)	66.2 (12.4)	67.2 (12.0)	0.004
Male, n (%)	2807 (71.9)	1258 (69.8)	0.109
Caucasian, n (%)	3286 (84.2)	1101 (61.1)	<0.001
Admission Diagnosis			
Acute coronary syndrome, n (%)	3627 (93.0)	1459 (81.0)	<0.001
Chest pain unknown cause, n (%)	253 (6.48)	271 (15.0)	<0.001
Other, n (%)	23 (0.59)	72 (4.00)	<0.001
Previous AMI, n (%)	1169 (30.0)	551 (30.6)	0.659
Previous Angina, n (%)	1555 (39.9)	661 (36.7)	0.024
Hypertension, n (%)	2505 (64.2)	1142 (63.4)	0.567
Hypercholesterolemia, n (%)	2167 (55.5)	877 (48.7)	<0.001
PVD, n (%)	297 (7.61)	164 (9.10)	0.062
Cerebrovascular Disease, n (%)	339 (8.69)	161 (8.93)	0.798
Asthma or COPD, n (%)	647 (16.6)	311 (17.3)	0.550
Chronic Renal Failure, n (%)	126 (3.23)	129 (7.16)	<0.001
Heart Failure, n (%)	147 (3.77)	111 (6.16)	<0.001
Enzymes Elevated, n (%)	3382 (86.7)	1611 (89.4)	0.004
Previous/ Current Smoker, n (%)	2600 (66.6)	1210 (67.1)	0.724
Cholesterol, mean (SD)	4.53 (1.38)	4.48 (1.33)	0.379
Diabetes			
Dietary control, n (%)	157 (4.02)	81 (4.50)	0.449
Oral medicine, n (%)	473 (12.1)	248 (13.8)	0.091
Insulin, n (%)	223 (5.72)	123 (6.83)	0.116
Insulin and medication, n (%)	59 (1.51)	62 (3.44)	<0.001
Previous PCI, n (%)	783 (20.1)	343 (19.0)	0.382
Previous CABG, n (%)	274 (7.02)	192 (10.7)	<0.001
Systolic BP, mean (SD)	136.6 (24.6)	140.3 (25.6)	<0.001
Heart Rate, mean (SD)	71.4 (15.4)	75.8 (18.0)	<0.001
Admitting Consultant			
Cardiologist, n (%)	3893 (99.8)	1708 (94.8)	<0.001
Other general physician, n (%)	4 (0.10)	65 (3.61)	<0.001
Other, n (%)	3 (0.08)	21 (1.17)	<0.001
Beta Blocker, n (%)	1458 (37.4)	713 (39.6)	0.118
Statin, n (%)	2034 (52.1)	988 (54.8)	0.061

For peer review only

Glucose, mean (SD)	7.45 (3.48)	8.16 (4.13)	<0.001
Height, mean (SD)	169.7 (9.90)	169.3 (10.1)	0.251
Weight, mean (SD)	82.7 (17.7)	82.6 (18.5)	0.890
Family History of CHD, n (%)	1942 (49.8)	774 (43.0)	<0.001
Creatinine, mean (SD)	97.6 (54.9)	103.4 (80.4)	0.006
Haemoglobin, mean (SD)	137.4 (18.0)	135.7 (19.6)	0.002
Killip class			
No evidence of Heart Failure, n (%)	3083 (79.0)	1108 (61.5)	<0.001
Basal Crepitations, n (%)	101 (2.59)	172 (9.54)	<0.001
Pulmonary Oedema, n (%)	139 (3.56)	105 (5.83)	<0.001
Cardiogenic Shock, n (%)	14 (0.36)	7 (0.39)	0.999
Cardiac Arrest, n (%)	100 (2.56)	46 (2.55)	0.999
Thienopyridene, n (%)	3384 (86.7)	1662 (92.2)	<0.001

AMI: Acute Myocardial Infarction; BP: Blood Pressure; CABG: Coronary Artery Bypass Graft; CHD: Coronary Heart Disease; COPD: Chronic Obstructive Pulmonary Disease; LVEF: Left Ventricular Ejection Fraction; MI: Myocardial Infarction; PCI: Percutaneous Coronary Intervention; PVD: Peripheral Vascular Disease

For peer review only

STROBE 2007 (v4) checklist of items to be included in reports of observational studies in epidemiology*
Checklist for cohort, case-control, and cross-sectional studies (combined)

Section/Topic	Item #	Recommendation	Reported on page #
**Title and abstract**	1	(*a*) Indicate the study's design with a commonly used term in the title or the abstract	Title page
		(*b*) Provide in the abstract an informative and balanced summary of what was done and what was found	2
**Introduction**			
Background/rationale	2	Explain the scientific background and rationale for the investigation being reported	5
Objectives	3	State specific objectives, including any pre-specified hypotheses	5
**Methods**			
Study design	4	Present key elements of study design early in the paper	6
Setting	5	Describe the setting, locations, and relevant dates, including periods of recruitment, exposure, follow-up, and data collection	6 and 8
Participants	6	(*a*) *Cohort study*—Give the eligibility criteria, and the sources and methods of selection of participants. Describe methods of follow-up<br>*Case-control study*—Give the eligibility criteria, and the sources and methods of case ascertainment and control selection. Give the rationale for the choice of cases and controls<br>*Cross-sectional study*—Give the eligibility criteria, and the sources and methods of selection of participants	6 and 7
		(*b*) *Cohort study*—For matched studies, give matching criteria and number of exposed and unexposed<br>*Case-control study*—For matched studies, give matching criteria and the number of controls per case	N/A
Variables	7	Clearly define all outcomes, exposures, predictors, potential confounders, and effect modifiers. Give diagnostic criteria, if applicable	6 and 7
Data sources/ measurement	8*	For each variable of interest, give sources of data and details of methods of assessment (measurement). Describe comparability of assessment methods if there is more than one group	*6*
Bias	9	Describe any efforts to address potential sources of bias	7
Study size	10	Explain how the study size was arrived at	N/A – registry data
Quantitative variables	11	Explain how quantitative variables were handled in the analyses. If applicable, describe which groupings were chosen and why	7
Statistical methods	12	(*a*) Describe all statistical methods, including those used to control for confounding	7
		(*b*) Describe any methods used to examine subgroups and interactions	7
		(*c*) Explain how missing data were addressed	7
		(*d*) *Cohort study*—If applicable, explain how loss to follow-up was addressed<br>*Case-control study*—If applicable, explain how matching of cases and controls was addressed	N/A

For peer review only

		Cross-sectional study—If applicable, describe analytical methods taking account of sampling strategy	
		(*e*) Describe any sensitivity analyses	N/A
**Results**			
Participants	13*	(a) Report numbers of individuals at each stage of study—eg numbers potentially eligible, examined for eligibility, confirmed eligible, included in the study, completing follow-up, and analysed	8
		(b) Give reasons for non-participation at each stage	8
		(c) Consider use of a flow diagram	N/A
Descriptive data	14*	(a) Give characteristics of study participants (eg demographic, clinical, social) and information on exposures and potential confounders	Table 1
		(b) Indicate number of participants with missing data for each variable of interest	Table 1
		(c) *Cohort study*—Summarise follow-up time (eg, average and total amount)	N/A
Outcome data	15*	*Cohort study*—Report numbers of outcome events or summary measures over time	Table 2 and throughout results section
		Case-control study—Report numbers in each exposure category, or summary measures of exposure	N/A
		Cross-sectional study—Report numbers of outcome events or summary measures	N/a
Main results	16	(*a*) Give unadjusted estimates and, if applicable, confounder-adjusted estimates and their precision (eg, 95% confidence interval). Make clear which confounders were adjusted for and why they were included	Table 2 and page 7
		(*b*) Report category boundaries when continuous variables were categorized	N/A
		(*c*) If relevant, consider translating estimates of relative risk into absolute risk for a meaningful time period	N/A
Other analyses	17	Report other analyses done—eg analyses of subgroups and interactions, and sensitivity analyses	Table 3 and throughout results section
**Discussion**			
Key results	18	Summarise key results with reference to study objectives	12
Limitations	19	Discuss limitations of the study, taking into account sources of potential bias or imprecision. Discuss both direction and magnitude of any potential bias	14 and 15
Interpretation	20	Give a cautious overall interpretation of results considering objectives, limitations, multiplicity of analyses, results from similar studies, and other relevant evidence	15
Generalisability	21	Discuss the generalisability (external validity) of the study results	15
**Other information**			
Funding	22	Give the source of funding and the role of the funders for the present study and, if applicable, for the original study on which the present article is based	Title page

For peer review only

*Give information separately for cases and controls in case-control studies and, if applicable, for exposed and unexposed groups in cohort and cross-sectional studies.
Note: An Explanation and Elaboration article discusses each checklist item and gives methodological background and published examples of transparent reporting. The STROBE checklist is best used in conjunction with this article (freely available on the Web sites of PLoS Medicine at http://www.plosmedicine.org/, Annals of Internal Medicine at http://www.annals.org/, and Epidemiology at http://www.epidem.com/). Information on the STROBE Initiative is available at www.strobe-statement.org.

For peer review only
